# Combining peak- and chromatogram-based retention time alignment algorithms for multiple chromatography-mass spectrometry datasets

**DOI:** 10.1186/1471-2105-13-214

**Published:** 2012-08-27

**Authors:** Nils Hoffmann, Matthias Keck, Heiko Neuweger, Mathias Wilhelm, Petra Högy, Karsten Niehaus, Jens Stoye

**Affiliations:** 1 Genome Informatics Group, Faculty of Technology, Bielefeld University, Bielefeld, Germany; 2 Department of Proteome and Metabolome Research, Faculty of Biology, Bielefeld University, Bielefeld, Germany; 3 Center for Biotechnology (CeBiTec), Bielefeld University, Bielefeld, Germany; 4 Plant Ecology and Ecotoxicology, Institute for Landscape and Plant Ecology, University of Hohenheim, Stuttgart, Germany; 5 Microbial Drugs, Helmholtz Centre for Infection Research, Braunschweig, Germany; 6 Bruker Daltonik GmbH, Bremen, Germany

## Abstract

**Background:**

Modern analytical methods in biology and chemistry use separation techniques coupled to sensitive detectors, such as gas chromatography-mass spectrometry (GC-MS) and liquid chromatography-mass spectrometry (LC-MS). These hyphenated methods provide high-dimensional data. Comparing such data manually to find corresponding signals is a laborious task, as each experiment usually consists of thousands of individual scans, each containing hundreds or even thousands of distinct signals. In order to allow for successful identification of metabolites or proteins within such data, especially in the context of metabolomics and proteomics, an accurate alignment and matching of corresponding features between two or more experiments is required. Such a matching algorithm should capture fluctuations in the chromatographic system which lead to non-linear distortions on the time axis, as well as systematic changes in recorded intensities. Many different algorithms for the retention time alignment of GC-MS and LC-MS data have been proposed and published, but all of them focus either on aligning previously extracted peak features or on aligning and comparing the complete raw data containing all available features.

**Results:**

In this paper we introduce two algorithms for retention time alignment of multiple GC-MS datasets: multiple alignment by bidirectional best hits peak assignment and cluster extension (BIPACE) and center-star multiple alignment by pairwise partitioned dynamic time warping (CeMAPP-DTW). We show how the similarity-based peak group matching method BIPACE may be used for multiple alignment calculation individually and how it can be used as a preprocessing step for the pairwise alignments performed by CeMAPP-DTW. We evaluate the algorithms individually and in combination on a previously published small GC-MS dataset studying the *Leishmania* parasite and on a larger GC-MS dataset studying grains of wheat (*Triticum aestivum*).

**Conclusions:**

We have shown that BIPACE achieves very high precision and recall and a very low number of false positive peak assignments on both evaluation datasets. CeMAPP-DTW finds a high number of true positives when executed on its own, but achieves even better results when BIPACE is used to constrain its search space. The source code of both algorithms is included in the OpenSource software framework *Maltcms*, which is available from http://maltcms.sf.net. The evaluation scripts of the present study are available from the same source.

## Background

Metabolomics, the study of an organism’s biochemistry, has become increasingly relevant along with other “omics” technologies during the last ten years. Some of the techniques of choice to distinguish the metabolites present in a biological sample of an organism are separation techniques coupled to sensitive detectors, such as gas chromatography-mass spectrometry (GC-MS) and liquid chromatography-mass spectrometry (LC-MS). In contrast to flame ionization detectors, UV absorbance detectors, and other one-dimensional detectors, these hyphenated methods provide high-dimensional data of analyte molecular ions or analyte molecular ion fragments collected over the runtime of the separation. In the context of metabolomics, this usually involves the observation of potentially hundreds of ion signals of different masses simultaneously in every recorded scan. These numbers may be even higher for proteomics, owing to the larger masses of peptides and peptide fragments. Comparing such data manually to find corresponding signals is very labour intensive, as each experiment usually consists of thousands of individual scans. Thus, the goal must be to obtain a high level of automation during data acquisition and data processing, allowing scientists to focus on the informative parts of their data, while still alerting them to potential errors or problems.

Often it is the goal of a metabolomics experiment to detect differences between a treated and a control group of measurements. Therefore, an accurate alignment and matching of corresponding features in all measurements is an extremely important part of data preprocessing. Data matrices representing the detected and aligned features across all measurements may be generated in order to be used for further statistical analysis. It is essential that an alignment algorithm captures fluctuations in the chromatographic system that lead to non-linear distortions of the retention time of individual features [1,2]. Further, it needs to group those features that are most similar to each other and to discover whether features are present or absent. In the end, a matrix of grouped peak features of single or related coeluting analyte ions should be generated to establish relationships in abundance between different experimental conditions. Then, based on other characteristics such as parent ion mass, ion fragments or isotope pattern, an identification of those features for integration with downstream analysis is required. Here we focus on the first few steps of such an analysis pipeline, including the generation of a matrix of grouped features for retention time normalization.

The currently available algorithms for retention time alignment can be distinguished into two general categories: peak-based and raw data-based alignment. The peak-based algorithms require prior peak- or feature-finding and often also peak deconvolution to reduce the effect of overlapping signals, before a score function is applied to establish correspondence between peaks [3-7]. Raw data-based algorithms on the other hand require little or no preprocessing, but are computationally very expensive [8,9]. We will now give a brief characterization of existing algorithms for the two categories before we introduce and categorize the algorithms presented in this paper.

**Peak-based algorithms** are very sensitive to the correctness of the a priori peak detection. A peak may be defined as the time-resolved signal intensity trace of an analyte ion’s corresonding mass matching predefined criteria, such as the goodness-of-fit to a predefined peak model shape, together with a signal-to-noise ratio threshold [7]. If a peak is tagged to be absent during preprocessing, it cannot be aligned by a peak-based algorithm. In order to handle missing peaks in data matrices for statistical analysis, Smith *et al.*[7] then filled the gaps by using estimates based on prior grouping of the data. Such a grouping usually consists of at least two groups, e.g. control and treated group. Then, for a peak missing within a group, where most other peaks are present, the missing value can be estimated from the present members of the group. However, such peak imputation may be erroneous if it is only based on the final peak tables and does not access the original data to ensure that a peak is really present.

To be able to assign peaks that may not have been aligned, Krebs *et al.*[6] proposed an approach based on prior peak detection and grouping, followed by polynomial interpolation to infer warping in between grouped peaks. Prince and Marcotte introduced a similar interpolation scheme for raw data-based alignment with dynamic time warping [8].

A further division of peak-based algorithms may also be applied concerning the use of mass spectra (MS) for peak similarity calculation. Warping based on peaks detected in the total ion chromatogram (TIC) is usually supplemented by using MS, to increase the number of true positive peak assignments [4,6,10]. Some algorithms work on a more complete set of extracted features, e.g. points of retention time, m/z and intensity [11,12], but often resort to linear regression in order to compute a retention time correction, due to the large amount of points that need to be processed. A more exhaustive overview of existing feature-based alignment algorithms to align point sets is given by Lange *et al.*[11], especially for the application to LC-MS data in proteomics and metabolomics. Aberg *et al.*[13] described the peak correspondence problem for NMR, showing that there is a significant amount of overlap considering the algorithms for these, at first sight different, application domains.

**Raw data-based algorithms** operate on the complete collection of (binned) MS data, also termed the *uniform matrix*, such as ObiWarp [8], which is based on dynamic time warping (DTW) with pairwise similarities between binned mass spectra, or the signal maps approach by Prakash *et al.*[9]. Therefore, these algorithms should find more and possibly better correspondences compared to the peak-based algorithms, which only have access to a limited amount of reported peak features. Other approaches use correlation optimized warping (COW) [14] for TIC alignment, or generalizations thereof [15,16], selecting specific mass traces to improve over simple TIC-based alignment. However, using many mass traces increases the computational demand, as well as the amount of data in need of processing, and may also increase the tendency of aligning noise [15]. Possibly owing to that computational demand, most raw data-based algorithms do not consider alignment or matching of individual points of retention time, m/z and intensity, but instead only try to correct the retention time deviation for each mass spectrum as a whole. The advantage of raw data-based methods is that they assign a definite position to each mass spectrum together with its corrected retention time after alignment. They use a pairwise similarity function between either TIC or sequences of mass spectra, finding an optimal global similarity with respect to their objective function [17-19]. The local correspondences between two raw data sets then allow to select the mass spectra with the highest pairwise similarities after the alignment to pinpoint peaks of interest for further investigation [8].

In this paper we introduce two novel methods for retention time alignment of multiple GC-MS and LC-MS experiments, which may be used individually and in combination as a hybrid method. The first method, bidirectional best hits peak assignment and cluster extension (BIPACE), is related to the clique-finding method described by Styczynski [4], but without relying on deconvoluted peaks and choosing a different criterion for peak correspondence and clique coherence, which drastically decreases computation times. It is a peak-based alignment method that automatically finds conserved groups of peaks among an arbitrary collection of chromatograms, based on the bidirectional best hit criterion as introduced by Tatusov *et al.*[20] and later by Overbeek *et al.*[21] for the matching of orthologous genes. Peaks are compared using user-definable similarities based on their mass spectra, for example with the similarity introduced by Robinson *et al.*[10], or by derived similarity functions, that we will introduce in this work, and are successively grouped into clusters of best pairwise correspondence. This method allows to find clusters of arbitrary size, up to the number of chromatograms under consideration. It may be applied to different experimental protocols with more than just two groups of treatment and control, since the algorithm requires no prior knowledge of an existing grouping.

The second method, center-star multiple alignment by pairwise partitioned dynamic time warping (CeMAPP-DTW), involves the application of DTW as in [8], but to all pairs of chromatograms. DTW was first introduced and used in speech recognition for the alignment of time dependent feature traces of speech samples [22-24]. One of the first applications of alignment methods to low-resolution GC-MS data was performed by Reiner *et al.*[25], based on the local squared distance of the TIC. More recent applications have been reported by Christin *et al.*[15], Clifford *et al.*[17], Prince and Marcotte [8], and Ramaker *et al.*[16]. Prince and Marcotte [8] showed that different local score or cost functions can be used in order to align data from LC-MS experiments with good performance. Other methods for the alignment of raw chromatographic data exist, such as aligning the time series data to a latent trace, which is constructed from training series, with an underlying stochastical model [26] or by different means of regression [27]. We use the grouped peaks from BIPACE as anchors to constrain the pairwise DTW alignments, as outlined in a previous publication [28]. This results in faster computation and at the same time considerably less memory usage than in the unconstrained cases through the use of an optimized data structure, while providing comparable alignment results. Building on the pairwise alignments, we choose the chromatogram with the highest sum of pairwise similarities as the reference for the final alignment of all remaining chromatograms to the reference. We use DTW to compute the pairwise alignment, due to its applicability to data with non-linear time scale distortions, its relatedness to classical sequence alignment algorithms [22-24] and its proven power to perform retention time correction and signal alignment [8,15,16].

## Methods

First we describe the peak and raw data-based alignment algorithms BIPACE and CeMAPP-DTW in detail. Then we combine them to create a new hybrid method that benefits from the speed and accuracy in peak matching of the peak-based alignment algorithm, while still providing a profile multiple alignment of all GC-MS datasets in reasonable time and space.

### BIPACE - multiple alignment by Bidirectional best hits peak assignment and cluster extension

Given a chromatogram *C *= {*p*_1_,*p*_2_,…,*p*_*ℓ*_} as an ordered set of peaks, we define a peak *p*=(**m**,**i**,*t*) as a triple of a mass vector **m**, an intensity vector **i**, both with the same dimensions, and a retention time *t*. Peaks can be matched between chromatograms by exhaustive search, if a feasible criterion for their identity exists. Based on GC-MS electron ionization (EI) fragmentation mass spectra alone, such a criterion is hard or even impossible to find especially due to the ambiguity of the mass spectra of isomers. Additionally, we have to deal with inherent noise, introduced by contaminations of the sample from external sources (sample preparation) or internal sources (sample injection, chromatographic system, MS acquisition). Thus, we use a proven similarity function, the modified cosine similarity between mass spectra, represented as (nominal) mass intensity vectors, weighted by an exponentially penalized difference in retention time (RT) (acquisition time) of the spectra [10]. For two peaks *p *= (**m**_*p*,_**i**_*p*,_*t*_*p*_) and *q *= (**m**_*q*,_**i**_*q*,_*t*_*q*_) and a retention time tolerance of *D*, following [10] we define this similarity function as:

(1) $$f(p,q):=s(p,q)\cdot \text{exp}\left(\frac{{\left(t_{p}-t_{q}\right)}^{2}}{2D^{2}}\right),$$

where *s* would typically be the cosine value of the angle between the two peaks’ mass spectral intensity vectors: *s*(*pq*) = cos*∠*(**i**_*p*,_**i**_*q*_). However, *s* could also be realized by any other similarity function defined between two vectors, such as the negative Euclidean distance, the dot product, Pearson’s linear correlation or Spearman’s rank correlation. The similarity function *f * leads to a good prefiltering of candidate peaks for matching throughout our input chromatograms.

In order to assign peaks to their best corresponding counterparts, we calculate all pairwise similarites using the similarity function *f * between all peaks from distinct chromatograms. The time required to calculate all pairwise similarities between peak candidates within the different chromatograms can be reduced by using a cutoff for the maximum allowed time deviation. This is achieved by first calculating the time deviation penalty, whose value ranges between 0, indicating a large RT difference, and 1 for perfect RT correspondence, and then deciding, based on that value, whether the proximity indicates a good candidate to go on and calculate the cosine score. However, the overall complexity for this first step remains quadratic in the number of peaks to be compared.

Apparently, the simplification should only be applied if the retention time deviation between two chromatograms is expected to be within a fixed time tolerance and as long as the order of elution of compounds is roughly preserved locally. Otherwise, potential candidates are pruned too early from the search space. Other similarity functions than *f * may also be applicable for some datasets. However, our experiments show, that *f * gives the best overall performance on undeconvoluted spectra with low mass resolution.

#### Assignment of peak pairs

We calculate the pairwise similarities using *f * as defined above for all possible pairs of peaks from *K* different chromatograms *C*_1_,*C*_2_,…,*C*_*K *_(partitions). This allows us to define a *K*-partite edge-weighted similarity graph *S *= (*V*,*E*), where each vertex in one of the *K* disjoint partitions represents a peak from a distinct chromatogram *C*_*j*_ and each edge represents a similarity value of a peak pair from two different partitions. Ultimately, we want to enumerate all cliques of *S*, a problem that relates to the classic *NP*-complete problem *CLIQUE*[29] with a runtime complexity that is unbearable for realistic problem sizes. We thus prune *S* using different heuristics to create the reduced weighted *K*-partite graph *S’*. *S’* is then used to construct the unweighted *K*-partite bidirectional best hit graph *S”*. On this special graph, the *CLIQUE* problem can be solved by a polynomial time algorithm since the maximum degree of each vertex in *S”* is always smaller or equal to *K*[30].

**Figure 1:**
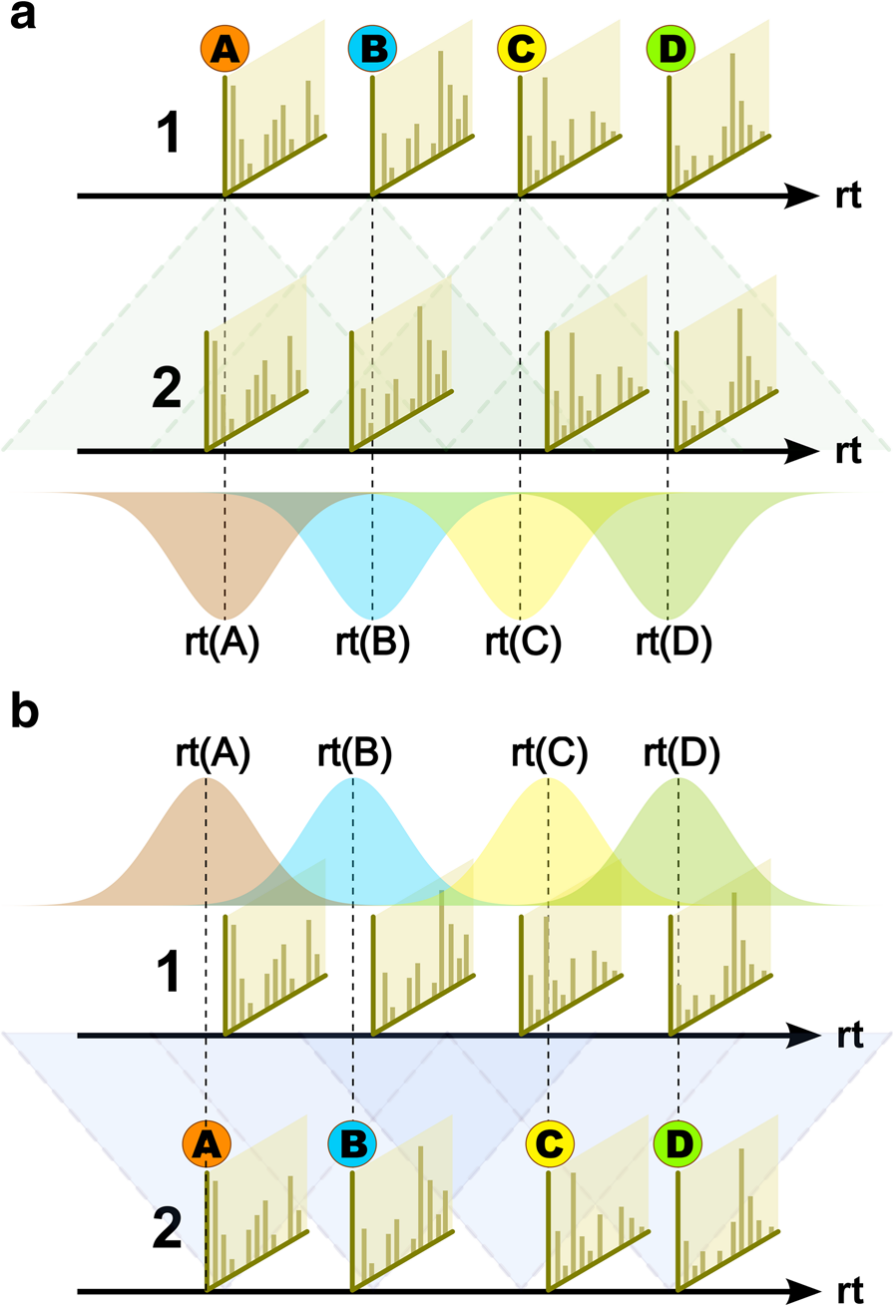
**Schematic of the forward and reverse similarity calculation phase of BIPACE.** The hard retention time difference limit is depicted by shaded cones with dashed outline. Individual Gaussian retention time penalty functions are mean centered on each peak’s apex retention time (rt). (**a**) BIPACE with a Gaussian retention time penalty function for peaks A through D from chromatogram 1 to chromatogram 2. (**b**) BIPACE with a Gaussian retention time penalty function for peaks A through D from chromatogram 2 to chromatogram 1 (reverse direction).

Since only the similarities between peaks of different chromatograms are considered by our algorithm, we do not calculate the self-similarity of peaks from the same chromatogram, which differentiates our method from the method of Styczynski *et al.*[4] and allows us to neglect all edges within partitions. Additionally, we exclude edges from *S* if they are outside the maximum retention time difference window as defined by *D*, which reduces the candidate space for peak matching, but may exclude valid peak assignments. Figures 1(a) and 1(b) show this examplarily for two peak lists. We then define *S’* as the graph with this reduced edge set *E’* and *V * as its vertex set.

#### Bidirectional best hits merging

In order to identify all bidirectional best hits (BBHs), that are all cliques of size 2 of *S’*, we look up for each pair of peaks *p*∈*C* and *q*∈*C*^*′*^ from distinct chromatograms *C* and *C’*, the peak with highest similarity to *p* in *C’*, denoted *q’*, and the peak with highest similarity to *q* in *C*, denoted *p’*. If *p *=* p*^*′*^and *q *=* q*^*′*^, then *p* and *q* are BBHs of each other and all peak similarities of *p* to other peaks in *q*’s parent chromatogram and of *q* to other peaks in *p’s* parent chromatogram are set to a minimum similarity value, while the similarity of the two associated peaks *p* and *q* is retained. We then define *V’* as the set of all vertices that are part of at least one BBH and define *S*^*′′ *^= (*V*^*′*^*E*^*′′*^) as the reduced *K*-partite graph with *V’* as its vertex set and *E”* as its unweighted BBH edge set. We now want to enumerate all maximal cliques of *S”*, a problem that is known to be solvable in polynomial time on graphs with a polynomial bound on the number of maximal cliques contained in the graph [30], as is the case for *S”* by construction.

**Figure 2:**
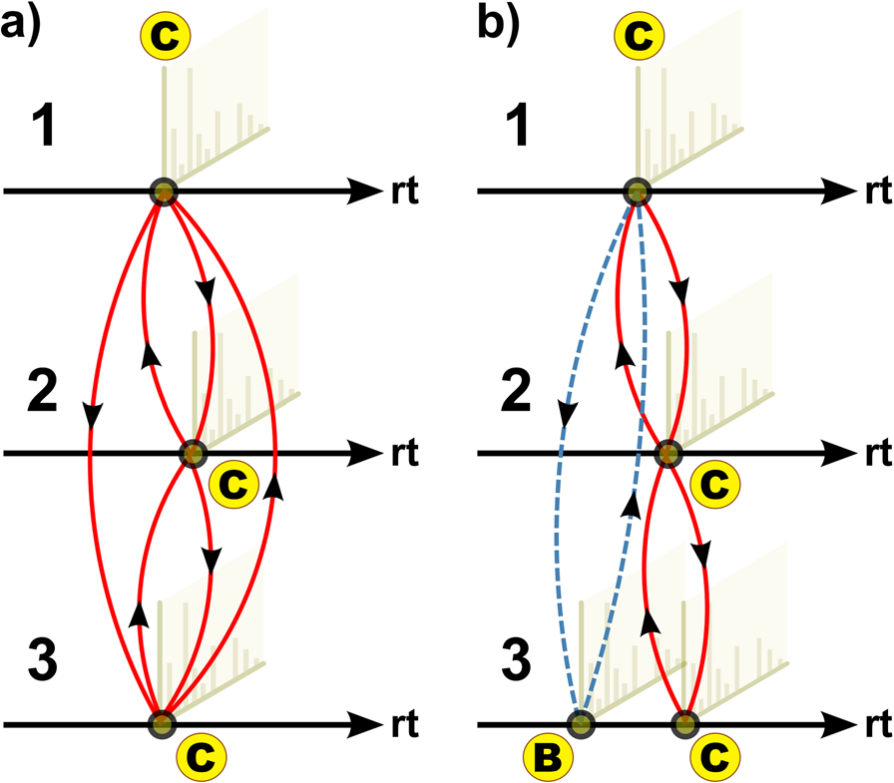
**Cliques after bidirectional-best hits have been evaluated with BIPACE.** Cliques after bidirectional-best hits have been evaluated with BIPACE. Subfigure (**a**) shows a complete clique of bidirectional-best hits of peak C in all three chromatograms. Subfigure (**b**) shows an incomplete case, where peak C in chromatograms 1 and 2, and in chromatograms 2 and 3 has a bidirectional-best hit. However, peak B in chromatogram 3 is only a bidirectional-best hit of peak C in chromatogram 1, destroying the possible complete clique of bidirectional-best hits between peak C in all three chromatograms.

We proceed greedily by trying to merge each pair of BBHs into a clique containing at least *k* and at most *K* peaks, where *k *≥ 2 is the minimal clique size (MCS) parameter. Merging is only performed if the new cluster remains a complete subgraph, which is equivalent to all peaks within the cluster being BBHs of each other. Otherwise, we select the largest common fully connected subgraph and omit all peaks that are not fully connected. We continue merging until all BBHs have been processed. Finally, we report cliques with at least *k* peaks ordered by their median retention time in a multiple alignment table. The clique finding is illustrated for three chromatograms and a limited number of peaks in Figure 2(a) for a maximal bidirectional-best hit clique and for a non-maximal clique with one not completely connected peak in Figure 2(b).

#### Time and space complexity of BIPACE

We need $\left(\begin{array}{c} K\\ 2 \end{array}\right)\ell^{2}$ comparisons to calculate all pairwise peak similarities between *K* chromatograms with *ℓ* peaks each, using a symmetric similarity function *f*(*p*,*q*) =* f*(*q*,*p*). Thus, the calculation of similarities requires $O(K^{2}\ell^{2})$ time and space, if we need to keep all pairwise similarities, e.g. for plotting purposes. However, we can save space by recording for every peak *p* from chromatogram *C*_*i*_ only its best hit set of size *K *− 1, containing the best matching peaks *q*_1_,*q*_2_,…,*q*_*K*_, where each *q*_*j*_ is from a different chromatogram *C*_*j*_,*j *≠* i*. Then, the total size of all best hit sets is proportional to the number of peaks, *Kℓ*, multiplied by the number of partitions a peak can have best hits for, *K *− 1, giving a total space requirement of $O(K^{2}\ell )$ for *S*’.

Finding the bidirectional best hit for each peak *p* of the *Kℓ *peaks in *S”* requires that we retrieve *p*’s best hit *q* and *q*’s best hit *p’*, and test whether *p *=* p*^*′*^. This amounts to O(Kℓ) comparisons for all peaks.

In order to identify all maximal cliques, we employ a greedy, bottom-up approach based on the BBHs of each peak. Storing all BBHs clearly requires O(Kℓ) space. Then, for each pair of peaks (*p*,*q*) from different partitions, we try to merge their corresponding cliques. This requires checking whether all peaks in the candidate cliques *P* and *Q* are fully connected, which takes 2|*P*||*Q*| comparisons per pair. Since |*P*| + |*Q*| ≤ |*K*|, this amounts to $O(K^{2}\ell^{2})$ time.

In total,BIPACE requires $O\;(K^{\;2}\ell^{\;2})$ time and $O\;(K^{\;2}\;\ell )$ space.

#### Multiple alignment projection

Up to now, only the grouped peaks have been aligned, so we have a peak-based multiple alignment. For a full multiple alignment of the complete datasets, all unassigned signals should also be aligned. In this situation, one could choose to implement an approach like the one proposed by Krebs and co-workers [6], selecting a *representative* chromatogram as alignment reference and calculating a cubic spline or other higher order polynomial, to interpolate between the aligned peaks. However, such a method can only work well if the number of aligned peaks is high and there are no large areas of unknown peak assignments in the chromatograms. To circumvent these problems, we will show in the next section how to use dynamic time warping (DTW) to calculate signal assignments in between paired peaks, using the same similarity function as in BIPACE. Additionally, we show how the aligned pairwise peak groups from BIPACE, or any other peak alignment method, can be used as alignment anchors for DTW, before using the pairwise DTW scores to automatically select a reasonable alignment reference using the center-star heuristic.

### CeMAPP-DTW - Center-Star Multiple alignment by Pairwise Partitioned Dynamic Time Warping

In this section, we introduce an improved version of DTW for series of time-resolved feature vectors, as they occur in GC-MS and LC-MS data processing. In [28], we described how to speed up DTW using predefined anchors of features which could be matched a priori with high confidence, while still allowing the alignment flexibility by defining a neighborhood radius *r* around the positions of the anchors. Here, we extend this approach and show how anchors can also be combined with other constraints, such as the Sakoe-Chiba Band constraint [23] to save both execution time and space, using an optimized data structure for alignment matrix storage.

Pairwise DTW is a global alignment of two series *A *= (*a*_1_,*a*_2_,…,*a*_*M*_) and *B *= (*b*_1_,*b*_2_,…,*b*_*N*_) of lengths *M* and *N*, respectively, where *a*_*i*_, $b_{i}\in R^{L}$ are the individual feature vectors of equal dimension *L*. In the context of GC- and LC-MS, a feature vector corresponds to a binned mass spectrum of intensities, a base peak ion intensity or a TIC value. We assume that mass resolution and range are equal for the experiments to align, thus only the intensity distribution over a fixed range of mass channels is used as feature vector.

The common definition of DTW involves a local distance function and a global distance or *objective* function that should be minimized [17]. To be consistent with our previous notation, we use an equivalent formulation using similarities, which then requires maximization of the objective function. Since *A* and *B* are series sampled at discrete intervals, we seek an optimal matching of elements (*ij*) connecting every element in *A* to at least one element in *B* and vice versa, termed a *path* or simply *alignment*. In order to find an optimal alignment of *A* and *B*, an (*M* + 1)×(*N* + 1) alignment matrix *Q* is set up, in which the optimal similarity value for aligning the prefixes (*a*_1_,…,*a*_*i*_) and (*b*_1_,…,*b*_*j*_) is stored at position *Q*(*ij*). A path $P=(p_{1},\ldots ,p_{K})$ thus consists of elements *p*_*k *_= (*ij*), where the path length *K* is bounded by 1 ≤* K *< 2·*max*(*M*,*N*) for non-empty *A* and *B*.

Pairwise DTW usually performs a global alignment of two series of features, requiring that the start and end of both series have to be aligned: *p*_1_ = (1,1) and *p*_*K *_= (*MN*). However, this constraint can be relaxed for subsequence matches to gain the equivalent of a free-end gaps alignment [8]. Note that DTW allows mapping of an element to multiple counterparts, which differentiates it from classical sequence alignment, where an element can only map to at most one counterpart [24]. Additionally, a continuity constraint requires that P must move only to directly adjacent cells of the alignment matrix vertically, horizontally or on the diagonal, such that if *p*_*k *_= (*ij*), and *p*_*k* + 1 _= (*i*^*′*^*j*^*′*^), then *i*^*′ *^−*i *≤ 1 and *j*^*′ *^−*j *≤ 1 must hold. A third constraint requires monotonicity of the path, such that *i*^*′ *^−* i *≥ 0 and *j*^*′ *^−* j *≥ 0 hold, and (*i*^*′ *^−* i*) + (*j*^*′ *^−* j*) > 0.

An optimal alignment path satisfying the above constraints maximizes the sum of pairwise similarities. This allows us to define the optimal DTW alignment between non-empty *A* and *B* through the following expression:

(2) $$\text{DTW}(A,B):=\underset{P\in P(A,B)}{\text{max}}\left(\underset{p_{i}\in P}{\sum }Q(p_{i})\right)$$

where P is the set of all possible global alignment paths of *A* and *B*.

Maximization alone would favor the highest number of steps to align *A* to *B*, given the above constraints, resulting in alternating combinations of vertical and horizontal steps. Hence, additional weighting factors need to be included to treat diagonal (*match*), vertical (*expansion*) and horizontal (*compression*) steps equivalently [24]. *Expansion* and *compression* are similar to *insertion* or *deletion* in classical sequence alignment. We thus define three weight parameters, *w*_*match*_, *w*_*comp*_ and *w*_*exp*_, which allow to vary the degree of flexibility of the alignment between overadaptation and the shortest possible alignment.

Finding an optimal warping path to actually recover the mapping between *A* and *B* can be achieved by applying the dynamic programming principle and tabulating intermediate optimal results. We thus calculate the value of each *Q*(*i*,*j*) by applying Equation 3 recursively, with *f * corresponding to the same similarity function as used in the section about BIPACE. Initialization of row 0 and column 0 with −*∞ *is required to only allow a global alignment, effectively forcing the alignment of (*a*_1_,*b*_1_).

(3) $$Q(i,j):=\left\{\begin{array}{ll} 0 & \text{if}\;i=j=0,\\ -\infty & \text{if}\;i=0\;\;\text{and}\;\;0<j\leq \text{N}\\ -\infty & \text{if}\;j=0\;\;\text{and}\;\;0<i\leq \text{M}\\ \max\left\{\begin{array}{l} Q(i-1,j-1)+w_{\text{match}}f(a_{i},b_{j})\\ Q(i,j-1)+w_{\text{comp}}f(a_{i},b_{j})\\ Q(i-1,j)+w_{\text{exp}}f(a_{i},b_{j}) \end{array}\right\} & \text{for}\;1\leq i\leq N,1\leq j\leq M \end{array}\right)$$

The optimal score can then be found in the bottom-right entry of the alignment matrix *Q*, such that *DTW*(*AB*) =* Q*(*MN*). We finally correct the optimal score for the weights to achieve a score that can be used to compare series of different lengths [8].

#### Postprocessing - obtaining bijective maps

As described in [8], the obtained map from DTW may not be bijective, depending on the similarity function used. The authors of [8] describe a method to select bijective anchors as control points for a polynomial fit, in order to interpolate in between the anchors. In CeMAPP-DTW, however, we choose to define path weights that either boost diagonal moves by user-definable factors, resulting in a less or more adaptive alignment path. For symmetric DTW, these factors can be used to efficiently reduce the problem of overadaptation of the path, when maximizing a similarity function and avoiding the need to predetermine additional gap penalties. CeMAPP-DTW reports a list of the maxima of the similarity function found along the alignment trace, which coincide with aligned, highly similar mass spectra.

#### An efficient datastructure for pairwise DTW alignment with anchors

The unconstrained pairwise DTW algorithm requires $O(N^{2})$ time and space, where *N* is the number of feature vectors to be compared. Additionally, due to the pairwise similarity used, the method requires another factor of *L* for each pairwise similarity calculation. For long feature vectors, *L* may be larger than *N*. However, most regions of the calculated pairwise similarities are never needed in practice, as chromatograms tend to be distorted most around the diagonal of such a pairwise similarity matrix. In practice, the Sakoe-Chiba band [23] or the Itakura parallelogram [22] constraints are often used to prune regions that are too far away from the diagonal.

These constraints still do not capture the chromatographic reality, where retention time distortion is mostly caused by large peaks eluting from the column, shifting all subsequent peaks by a nonlinear factor [1]. We therefore introduced easily identifiable peaks as anchors to DTW [28]. These anchors define regions within which the alignment is calculated exactly, whereas outside of these regions no calculations are performed at all. In order to implement this idea, here we introduce a partitioned array data structure to store only those elements that are contained in the anchor-constrained regions. This requires the previous association of anchors, e.g. by BIPACE or other methods.

#### Efficient storage of partitioned array

We use the row compressed storage (RCS) technique to store all elements of an alignment matrix in a linear array *d*, where each element is accessed via an offset index array *idx* for each row in the virtual matrix and a length *len* for the number of elements stored contiguously in that row. An element of the virtual matrix at row *i* and column *j* can be accessed using the index *k *=* idx*(*i*) + *j* in array *d*. Iteration for virtual row *i* can be performed from *idx*(*i*) to *idx*(*i*) + *j*,*j *<* len*(*i*). Query of elements outside of the defined regions returns a configurable default value, such as positive or negative infinity. Setting of such elements has no effect, since the layout is static and determined before initialization of the matrices.

#### Layout calculation

The layout of the partitioned array is determined by explicit constraints, regarding the elements that require evaluation during the alignment. These constraints are defined by geometric primitives within the 2-dimensional plane, e.g. rectangular regions defined by the alignment anchors, as well as trapezoid or arbitrary other regions. However, the layout needs to satisfy the monotonicity and continuity constraints of DTW. Thus, directly neighbouring adjacent anchors and anchors with inverted order are detected and removed.

The final shape of the partitioned array is determined by the intersection of the set of constraints L, where L consists of all pairs (*i*,*j*) for which the alignment is calculated. This may lead to a less optimal alignment concerning the optimization function, but allows for further speedup and smaller memory footprint. One option here is to include either a global or a local Sakoe-Chiba band constraint between successive anchors. The width *w* for such bands can be defined by the user either for the whole alignment matrix (global) or for every partition (local).

We then define $\hat{Q}$ as the DTW recursion to calculate *Q* using the constraint set L:

(4) $$\hat{Q}(i,j):=\left\{\begin{array}{cl} -\infty & \text{if}\;(i,j)\notin L\\ Q(i,j) & \text{otherwise.} \end{array}\right)$$

**Figure 3:**
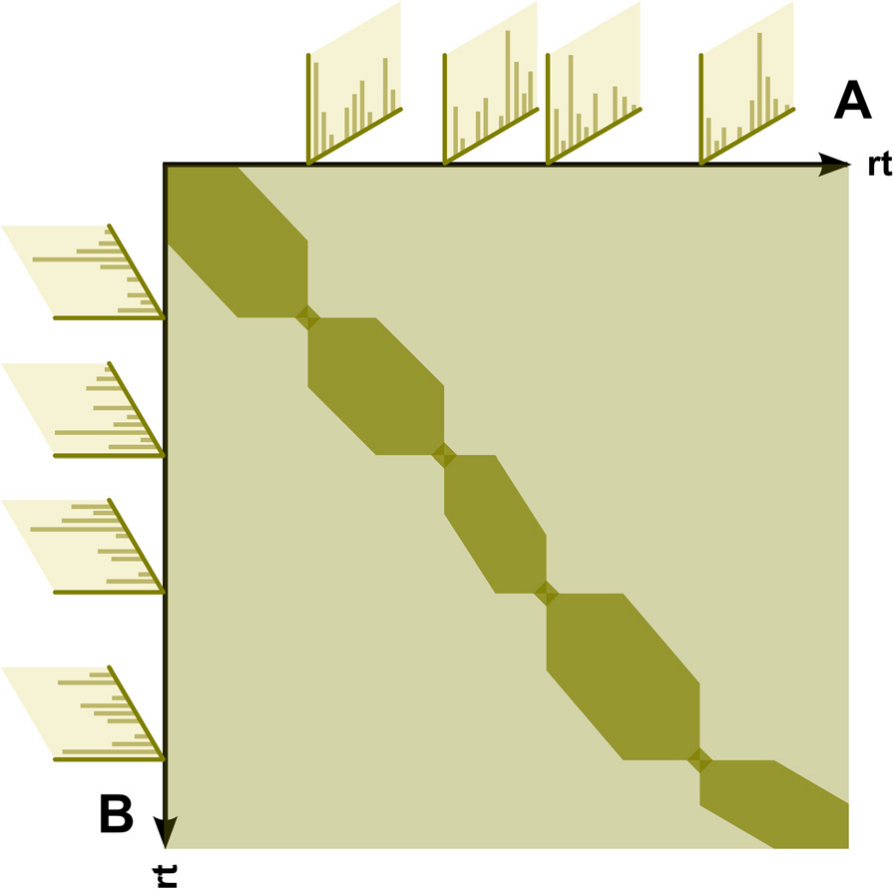
**Schematic alignment matrix of partitioned dynamic time warping.** Schematic of a pairwise alignment matrix of partitioned dynamic time warping for two arbitrary chromatograms ***A*** and **B**. The light shaded region represents the unconstrained alignment matrix, whereas the dark shaded areas represent the constrained partitions. For every pair of predefined anchors, in this case depicted as mass spectra, a small region around the anchor is kept to allow the alignment a higher degree of flexibility. Each partition is additionally constrained by a local Sakoe-Chiba band constraint. The intersection of all constraint sets L defines the final layout of the pairwise alignment matrix and thus the number of elements that are compared and stored.

A schematic of the corresponding partitioned array with a constraint set L using anchors and a local Sakoe-Chiba band constraint is shown in Figure 3.

### Multiple alignment of chromatograms

In order to capture machine dependent fluctuations in retention times and signal intensities, multiple chromatograms are usually measured from the same original sample as technical replicates. These often exhibit notable, but rather small deviations in retention times and intensities, when compared pairwise.

However, biological replicates show larger deviations due to the heterogeneity of the sampled population and corresponding differences in the metabolic state of cells at the time of harvesting [15].

When comparing the metabolic response of an organism under different conditions, deviations are even larger, as some metabolites may not occur at all, and others occur in different quantities, depending on the affected pathways of the organism. Thus, a multiple alignment algorithm needs to handle all of these aspects as good as possible.

#### Reference selection

A general method for multiple alignment of chromatograms does not necessarily require a reference to align to. However, most published algorithms either use a manually selected reference [3], or construct a reference by adding otherwise unassigned peaks [5] or by averaging over total ion chromatograms [17]. Automatic selection of a reference among the available chromatograms is seldomly reported [31] but is beneficial to methods using a manually defined reference [10] that can introduce a bias in the process of alignment early on.

In metabolomics and proteomics applications, the number of measurements typically ranges from dozens to hundreds, such that a multiple alignment algorithm should scale well and be as memory efficient as possible, since file sizes may approach several hundred MBytes or even GBytes per raw data file. To avoid a direct multiple alignment, we calculate pairwise DTW scores between all pairs of chromatograms first. These scores can be obtained from the pairwise DTW scores, but faster methods can also be used to estimate the true scores, e.g. based on peak-matching and scoring as performed by BIPACE, although these may not be as accurate. Then, we select the chromatogram that has the highest sum of scores to all other chromatograms as the alignment reference. All remaining chromatograms are then aligned to this center chromatogram independently of each other [28]. Other authors report to use comparable clustering methods [5,15].

#### Multiple alignment construction

The construction of the multiple alignment differs slightly from the approach taken in standard sum-of-pairs multiple sequence alignment, since we use DTW, which is potentially a non-metric similarity function [32]. Additionally, every pairwise alignment is a global alignment without gaps, so in principle we can not worsen the multiple alignment by introducing gaps. However, since DTW uses compressions and expansions, chromatograms having peaks which are absent in the selected reference may artificially decrease the quality and score of the alignment. Hence, we can not guarantee that the multiple alignment will be within a specific error bound of the optimal multiple alignment. Nontheless, our method performs well in practice, which will be discussed in detail in the Results section.

We finally obtain a dense matrix of aligned feature vector indices, e.g. of the binned mass spectra, or derived figures, such as the retention time of each mass spectrum for all chromatograms. In case of CeMAPP-DTW, and in contrast to BIPACE, there are no missing features within the table, as all features are aligned. These matrices will be used for evaluation of the alignment performance.

#### Time and space complexity of CeMAPP-DTW

Following the notation for time and space complexity of BIPACE, we need $O(K^{2}\ell^{2})$ comparisons to calculate all pairwise alignments between *K* chromatograms with *ℓ*mass spectra each. Using the pairwise DTW alignment similarities, we select the center chromatogram in O(K) time and align all remaining *K*−1 chromatograms to it in O(Kℓ) time. If we store the pairwise alignments, they can be reused at this point, otherwise, they need to be recalculated in $O(K\ell^{2})$ time. Thus, the calculation of all unconstrained pairwise DTW alignments takes $O(K^{2}\ell^{2})$ in time and space.

For partitioned DTW, the runtime and space requirements for each pairwise alignment are a function of the partition length *s* and of *ℓ*. We then need O(ℓs) time and space to calculate each pairwise alignment. Using an additional local Sakoe-Chiba band constraint with width *w*, the space and time requirements for partitioned DTW are O(ℓw). In total CeMAPP-DTW then requires $O(K^{2}\ell w)$ time and space.

## Results

In this section, we first give a short review of existing strategies for the evaluation of peak and profile-based multiple alignment algorithms in the context of metabolomics. We then describe our approach and define useful metrics to compare alignment quality before we evaluate BIPACE and CeMAPP-DTW on two metabolomics datasets. In order to evaluate our methods we need to define what a *good* alignment is. To achieve this, we can use a ground truth of highly conserved and putatively grouped peaks, which are confirmed by MS/MS. For LC-MS in the domain of metabolomics and proteomics, such data sets were prepared and used for the evaluation of alignment algorithms [11]. However, the ground truth defined by these datasets is only well defined for feature-based alignments and also requires a grouping of individual mass-to-charge ratio (m/z), retention time (rt) and intensity features, which are currently not provided by either BIPACE or CeMAPP-DTW. For GC-MS metabolomics data, Robinson *et al.*[10] compare their method against a ground truth defined by a human specialist.

Each alignment evaluation requires ground truth files, containing grouped features, such as triples of m/z, rt and intensity in the case of Lange *et al.*[11], and simply rt in the case of Robinson *et al.*[10]. In the first case one scan may have multiple features, while in the second case a scan is a feature that is only identified by its rt. In order to perform the evaluation, we focused on the correctly assigned rts and the corresponding scan indices, since those will usually have the largest deviation across samples.

The ground truth peak group defines whether a peak is present in a sample or absent. The results of an alignment algorithm are then tested in turn against each ground truth group. If the alignment algorithm reports an aligned peak group, we count all of the group’s peaks that are present in the corresponding ground truth group as true positives (*TP*). Peaks that are absent in the ground truth group and in the reported peak group are counted as true negatives (*TN*). A peak that is reported as absent in the ground truth group, but as present in the alignment algorithm’s reported group, is recorded as a false positive (*FP*). Finally, if a peak is reported as present in the ground truth peak group, but as absent in the reported peak group, it is reported as a false negative (*FN*).

We then use the following commonly applied measures to assess the quality of a multiple alignment:

(5) $$\text{Precision}=\frac{\text{TP}}{\text{TP}+\text{FP}}$$

(6) $$\text{Recall}=\frac{\text{TP}}{\text{TP}+\text{FN}}$$

(7) $$F1=2\cdot \frac{\text{Precision}\cdot \text{Recall}}{\text{Precision}+\text{Recall}}$$

We evaluate the performance of BIPACE and Robinson’s method using precision and recall, as well as the total *TP* and *FP* numbers. For CeMAPP-DTW, however, the *TN* and *FN* values are not available, since CeMAPP-DTW reports an alignment for all peaks, so we will compare CeMAPP-DTW only using absolute *TP* and *FP* numbers.

**Figure 4:**
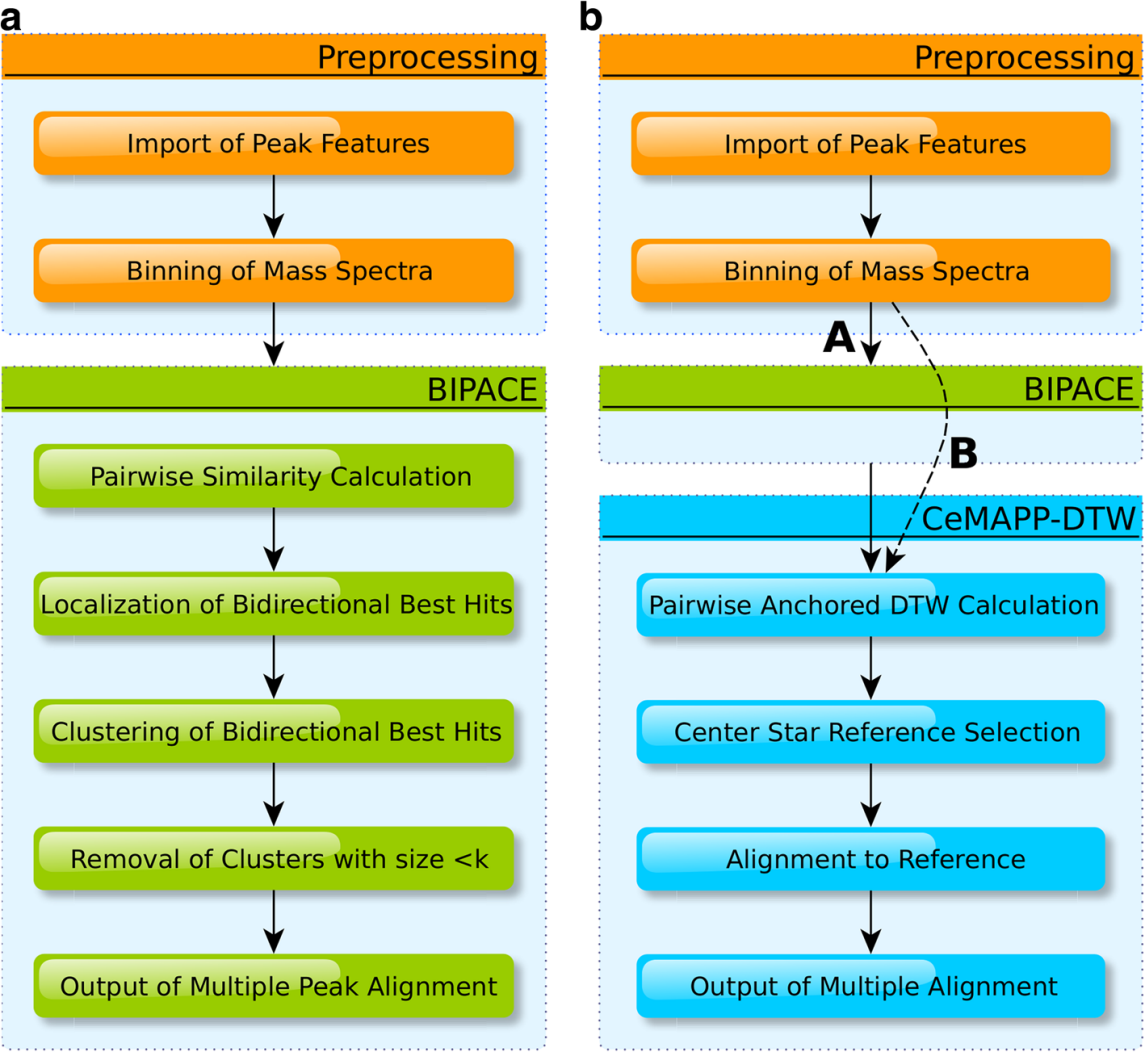
**Workflow for evaluation of BIPACE and CeMAPP-DTW. **(**a**) Sequence of preprocessing commands for evaluation of BIPACE. (**b**) Sequence of preprocessing commands for evaluation of CeMAPP-DTW. Path indicated with (A) with and (B) without anchors.

The three major configurations that we will evaluate are schematically shown in Figure 4. We evaluate each of BIPACE and CeMAPP-DTW individually, before we evaluate CeMAPP-DTW using the BIPACE alignment with the highest *F*1 score as a constraint set. The actual alignment is preceded by a preprocessing phase, in which the peak features are imported and converted for use in our pipeline. Then, BIPACE is applied with its processing steps to calculate a multiple alignment, before CeMAPP-DTW is used first without anchors and then with the anchors as defined by the best multiple alignment of BIPACE. Throughout all evaluations, we used five different local similarities to compare the binned mass spectra, namely the cosine (*cosine*), the dot product (*dot*), the negative Euclidean distance (*euclidean*), Pearson’s linear correlation (*linCorr*), and Spearman’s rank correlation (*rankCorr*), each with and without a retention time penalty, as defined in Equation 1.

### Evaluation of BIPACE and CeMAPP-DTW on a reference dataset

We evaluated the BIPACE method on the *Leishmania* parasite raw data and peak lists published in [10], using as ground truth the manual multiple alignment reference from the same paper.

#### Data preparation and parameter settings

Preprocessing was performed by removing intensites linked to the derivatization agent at masses 73 and 147. Due to lack of access to the manually edited peaks lists, we used the ChemStation (Agilent Technologies) peak data provided as supplementary material directly and imported them as peak annotations into our processing pipeline. The peak data files contained between 169 and 174 peaks and were stored in tab delimited format. A line in such a file reported the apex scan index of the corresponding peak for retrieval of the raw mass spectra from the 8 different ANDI-MS/netCDF chromatogram files. Each of these files contained approximately 2780 centroided mass spectra. The spectra were binned with nominal mass accuracy in a range from 50 to 550 Dalton for further processing.

The reference manual alignment containing 173 aligned peak groups was then used in order to calculate the classification performance numbers, as defined in Equation 5. This was performed for each multiple alignment reported by either BIPACE or CeMAPP-DTW individually, or in conjunction, where CeMAPP-DTW used the multiple alignment of BIPACE as anchors, following Figure 4.

**Figure 5:**
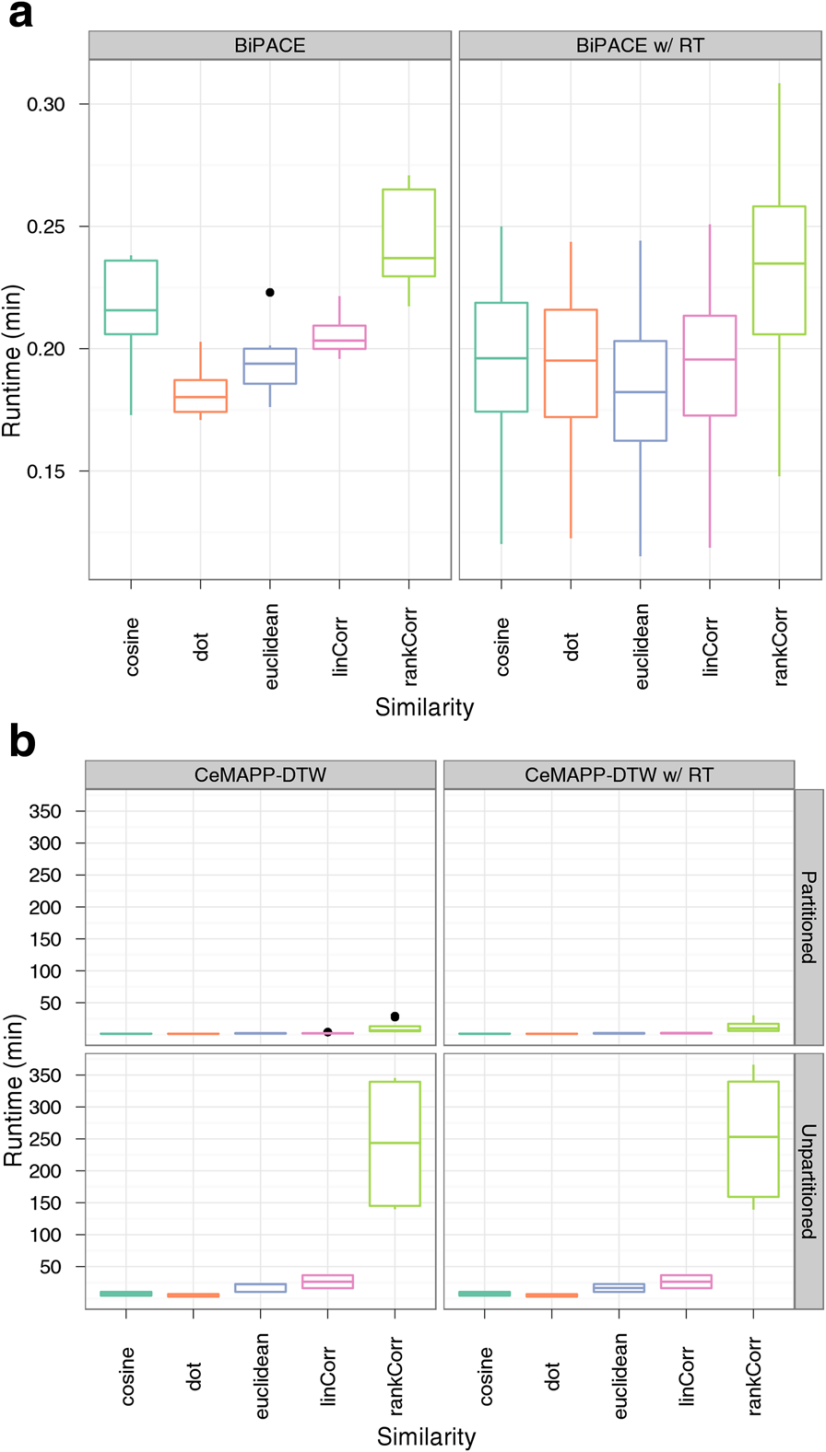
**Boxplots of the runtimes of BIPACE and CeMAPP-DTW for the *Leishmania *dataset.** Boxplots of the runtimes of (**a**) BIPACE and (**b**) CeMAPP-DTW for the *Leishmania* dataset.

We varied the minimum clique size (*MCS*) parameter from 2 to 8 chromatograms in order to control the size of the smallest clique that should be reported by BIPACE. Other varying parameters for the time penalized instances included the width parameter *D* of the retention time penalty function, as defined in Equation 1. We also used a threshold parameter *T* on the value of this function so that the costly pairwise similarity function was only evaluated if the retention time penalty function’s value was greater or equal to *T*. This pruning leads to lower runtimes of BIPACE and CeMAPP-DTW, visualized in Figure 5.

For CeMAPP-DTW, we assessed two different approaches, one without any anchors from BIPACE, and one using the anchors as reported by the best BIPACE instance, as determined by the *F*1 measure. Each CeMAPP-DTW configuration was further parameterized on the weight *W * used for diagonal matches and on the Sakoe-Chiba band constraint *BC*, given as the percentage of scans from a chromatogram. For those CeMAPP-DTW instances which used the best BIPACE anchors, we additionally varied the use of the Sakoe-Chiba band to be applied globally or locally and the size of the radius around anchors. In total, we evaluated 3106 different parameterizations.

The exact configuration and evaluation results for all parameterizations together with memory usage details are available in Additional file 1.

#### Results of BIPACE

**Figure 6:**
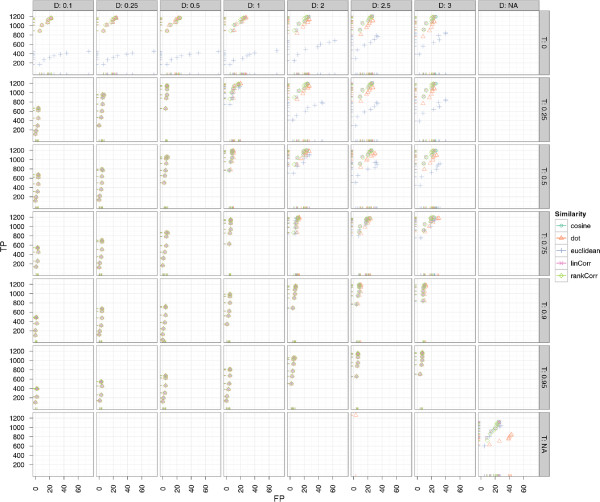
**Scatter plots for BIPACE for the *Leishmania *dataset with alignment false positives and true positives for the *D *and *T *parameters.** Scatter plots for BIPACE for the *Leishmania* dataset with alignment false positives and true positives conditioned on retention time tolerance *D* (columns) and retention time threshold *T* (rows). Instances without retention time penalized similarity function are shown in the *NA* row/column for reference. It is visible that the unpenalized instances perform consistently worse on true positives and false positives.

**Figure 7:**
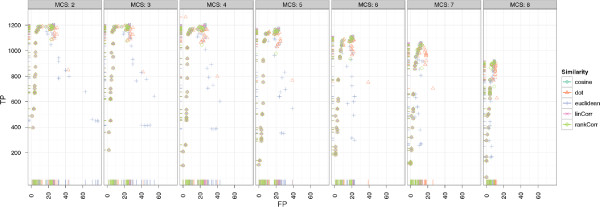
**Scatter plots for BIPACE for the *Leishmania *dataset with alignment false positives and true positives for the *MCS *parameter.** Scatter plots for BIPACE for the *Leishmania* dataset with alignment false positives and true positives conditioned on the minimum clique size (*MCS*) parameter (columns). The highest number of true positives is found for the smallest possible value of *MCS *= 2. Fewer false positives are obtained for higher values of *MCS* losing true positives.

Our results for BIPACE show good performance for the time-penalized dot product, which was also used for Robinson’s method, but also for the time-penalized variants of Pearson’s linear correlation (*linCorr*) and Spearman’s rank correlation (*rankCorr*). All instances using a time-penalized variant of the similarity function are indicated in the *similarityFunction* column of Additional file 1: Table S1 and are shown in Figure 6 for varying *T* and *D* parameters. The impact of the different similarity functions on the runtime of BIPACE can be seen in Figure 5(a), showing that for BIPACE the runtime median was close to 38 seconds, while it was reduced for BIPACE with retention time penalty *D* and threshold *T* to less then 10 seconds. Our best result is achieved for BIPACE with Pearson’s linear correlation as pairwise similarity using the time penalized variant with a minimum clique size of *MCS *= 2, *T* of 0.25 or 0.0 and *D* of 2.5 seconds. The results of the cosine similarity function are equal. For these best cases, we achieve 1206 true positives, 26 false positives, 28 false negatives and 84 true negatives. This results in a precision of 0.98, a recall value of 0.977, and a *F*1 value of 0.978. Figure 7 indicates that, for the best performing similarities, the choice of the *MCS* parameter is not critical, unless a false positive number of 0 is wanted.

**Figure 8:**
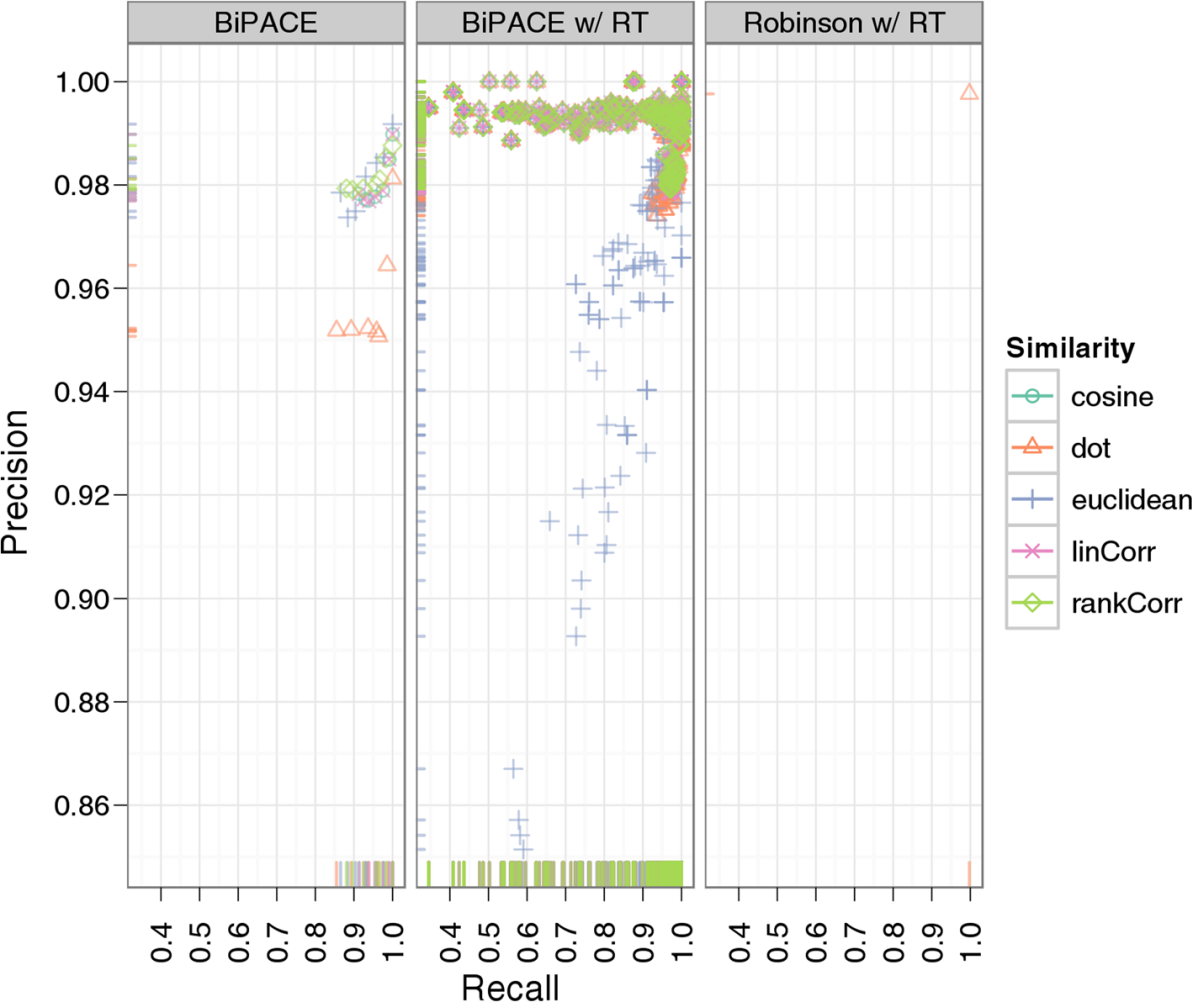
**Scatter plots for BIPACE for the *Leishmania *dataset with alignment precision and recall.** The retention time penalized variant of BIPACE performs better than the plain variant using the rank or linear correlation similarities. The published alignment of Robinson [10] performs best using a time penalized dot product similarity.

Figure 8 shows that Robinson’s [10] result performs better than any of our parameterized instances and achieves 1264 true positives and at the same time only 3 false positives. Additionally, 3 false negatives and 114 true negatives improve the precision to 0.9976 and the recall to 0.9976, giving an *F*1 value of 0.9976. An explanation for this result can be found in our best performing alignments. There we see a larger number of false positives, meaning that our method reports more potential matches, which are scored as false positives against the given reference, but would otherwise be true positive matches. Thus, we suspect that Robinson’s manually defined ground truth that we evaluate against is probably not error-free. Additionally, our best parameterizations report a number of potential aligned peak groups with significant sizes, which are not contained in the reference at all and are thus not assignable for the evaluation. If only the number of false positives is important, for example to retrieve only highly conserved peak groups with as few errors as possible, a number of parameterizations achieve that goal with 488 true positives and only 1 false positive assignment with maximum clique size of 8, a retention time threshold *T* of 0.9 and retention time penalty *D* of 0.1.

#### Results of CeMAPP-DTW

**Figure 9:**
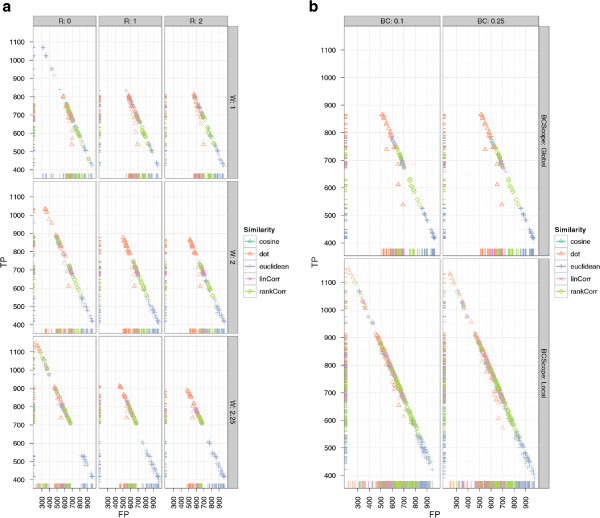
**Scatter plots for CeMAPP-DTW for the *Leishmania *dataset with alignment true positives and false positives.** SubFigure 9(a) shows alignment true positives and false positives conditioned on anchor radius (columns) and alignment match weight (rows). It is clearly visible that an anchor radius of *R *= 0 combined with a match weight of *W *= 2.25 gives the best results for linear correlation and the dot product. SubFigure 9(b) shows alignment true positives and false positives conditioned on Sakoe-Chiba bandwidth constraint as relative number of scans (columns). Rows show whether the constraint was applied globally, indicated as *FALSE*, or locally, indicated as *TRUE*. The best results were obtained for a local window of *SC *= 0.1·max{|*A*|,|*B*|}.

The best scoring CeMAPP-DTW result using the dot product as a pairwise similarity with diagonal match weight *W * of 2.25, a local Sakoe-Chiba band of *BC *= 0.1 and *D *= 3, using the anchors as defined by the best-scoring BIPACE instance with an anchor radius of 0 achieves 1149 true positives and 219 false positives. However, the number of false positives is potentially exaggerated since the manual reference alignment contains absent peaks, which are of course reported by CeMAPP-DTW and are thus counted as false positives. The best CeMAPP-DTW result used the dot product without using anchors and a match weight of 2.25, a global Sakoe-Chiba band of *BC *= 0.1 and *D *= 2.5 and achieved 739 true positives and 549 false positives. The results for CeMAPP-DTW are visualized in Figure 9(a) for varying match weight *W * and anchor radius *R* and in Figure 9(b) for varying global or local (*BCScope*) Sakoe-Chiba band constraint *BC*.

### Evaluation of BIPACE and CeMAPP-DTW on a real world dataset

In order to assess the quality of BIPACE and CeMAPP-DTW with and without BIPACE anchors on a GC-MS dataset of a more realistic size, we used samples from a plant metabolomics experiment [33]. Spring wheat (*Triticum aestivum* L.) was grown under atmospheric and increased *CO*_2_ concentration conditions [34] in a free-air carbon dioxide (*CO*_2_) enrichment (FACE) field experiment. The wheat was grown, harvested, and sampled at maturity in two successive years (2005, 2006), and prepared for analysis with GC-MS according to the protocol published in [33] in order to determine whether the plants showed a metabolic response in their grains evident through *CO*_2_enrichment.

Our evaluation was based on a total of 40 chromatograms and 10 interspersed blank chromatograms. Each year was represented by 20 chromatograms, divided into two groups of 5 chromatograms each, with one technical replicate per chromatogram, summing to 10 chromatograms per condition and year. Blank runs were excluded from this evaluation. The chromatograms contained between 4615 to 4685 centroided mass spectra. The maximal scan difference that we found was around 50 scans which amounts to a maximum retention time deviation of 32 seconds between the groups of 2005 and 2006.

#### Data preparation and parameter settings

The acquired raw data was exported using the ANDI-MS/netCDF export function of the Xcalibur software (Thermo Fisher Scientific Inc.). For all further preprocessing steps, we used our framework *Maltcms*. The data was first binned along the mass axis with nominal mass accuracy by arithmetic rounding to create a dense signal matrix. Then, for each signal matrix individually, the intensities were normalized to length one for each column (binned mass spectrum) to remove linear scaling effects in intensities.

In order to assess the grouping performance, we performed a peak detection with XCMS [7], using the matched filter method with a signal-to-noise ratio of 5 and full-width at half height of 5 in order to find well represented peaks. The peak finding step reported between 410 and 465 peaks per chromatogram. The apex scan indices for each chromatogram’s peaks were stored in one tab separated value file for each chromatogram.

We then chose signals within a retention time window of + /−30 seconds. To be counted as a complete group, the scans corresponding to the tags were required to have a pairwise cosine similarity between their binned mass spectra of >0.99 throughout all chromatograms and a maximum mass deviation of 0.01 Dalton. The selection process lead to 184 peak groups containing peaks appearing in all chromatograms, which defined our ground truth for the evaluation of the multiple alignments produced by our methods. This reference selection and grouping was performed by a profiling method, which was recently added to MeltDB [35].

The evaluation was then performed following the flowchart in Figure 4. BIPACE was run using the raw ANDI-MS/netCDF files as input together with the tab separated value peak lists. Subsequently, the CeMAPP-DTW instances without anchors from BIPACE were run, before finally the CeMAPP-DTW instances using the BIPACE anchors from the best scoring multiple peak alignment were executed.

The reference data was then compared to the alignment results generated by the three separate evaluation workflows for BIPACE, CeMAPP-DTW, and BIPACE+CeMAPP-DTW using the five different similarity functions mentioned at the beginning of the Results section, all of them plain and in combination with a retention time penalty, as described by Robinson *et al.*[10], who only report use of the time penalized dot product. We combined each similarity function with the time penalty function as in Equation 1.

**Figure 10:**
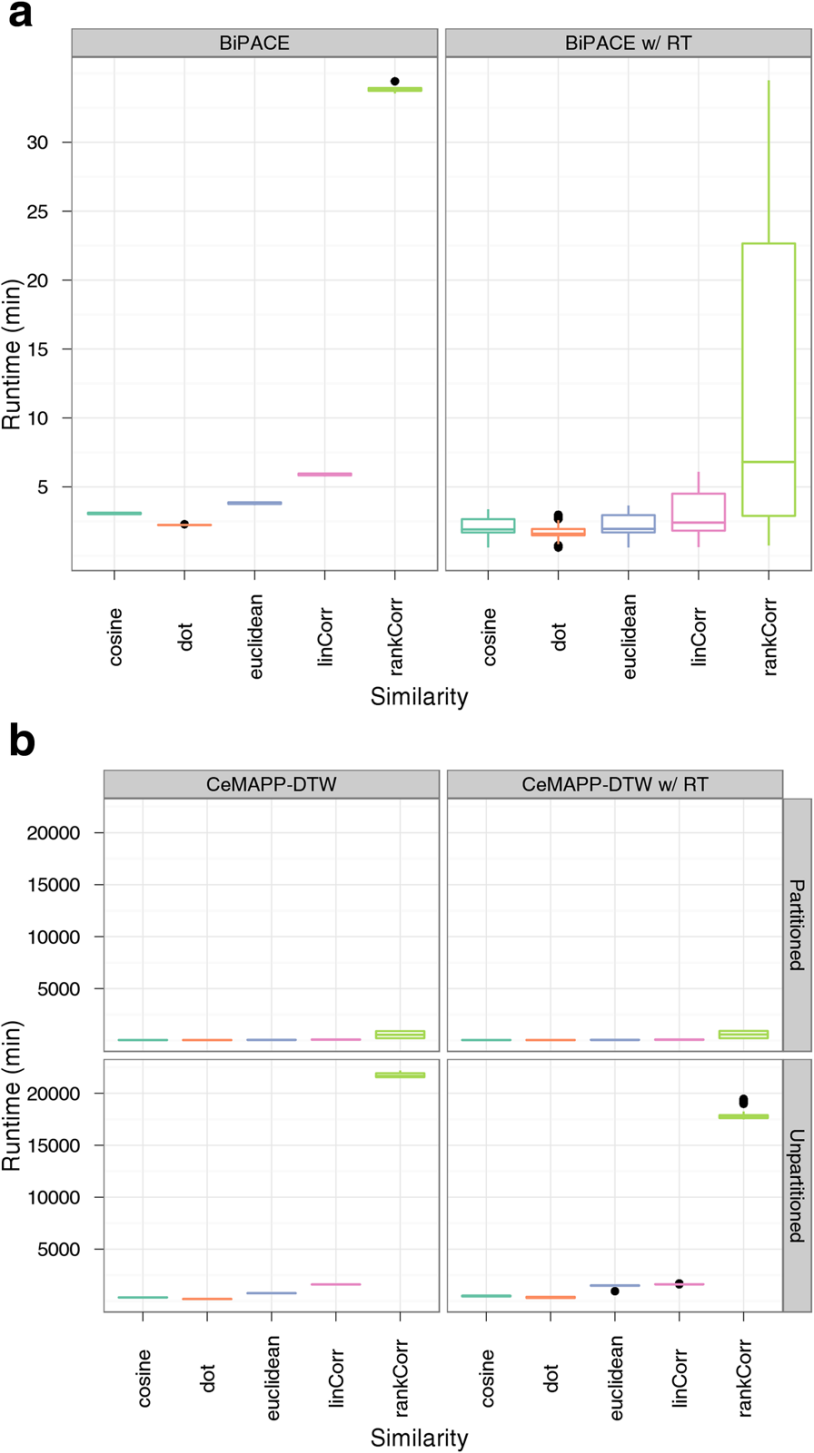
**Boxplots of the runtimes of BIPACE and CeMAPP-DTW for the wheat dataset.** Boxplots of the runtimes of (**a**) BIPACE and (**b**) CeMAPP-DTW for the wheat dataset.

In order to assess the precision of BIPACE, we started with a minimum clique size (*MCS*) parameter value of 40 chromatograms, meaning that only those groups that contained exactly one peak from each file were reported. For the time penalized instances we varied the width parameter *D* of the retention time penalty function. We also used the threshold parameter *T* on the value of this function so that the costly pairwise similarity function was only evaluated if the retention time penalty function’s value was greater than or equal to *T*. The positive effect of this pruning on the runtime of BIPACE and CeMAPP-DTW is visible in Figure 10.

For CeMAPP-DTW, we assessed two different approaches, one without any anchors from BIPACE, and one using the anchors as reported by the best BIPACE instance, as determined by the *F*1 measure. Each CeMAPP-DTW configuration was further parameterized on the weight *W * used for diagonal matches and the Sakoe-Chiba band constraint width *BC*, given as the percentage of scans from a chromatogram. For those CeMAPP-DTW instances which used the best BIPACE anchors, we additionally varied the use of the Sakoe-Chiba band to be applied globally or locally and the size of the radius around anchors.

The exact configuration and evaluation results for all 1641 parameterizations including memory usage are available in Additional file 2.

#### Results of BIPACE

**Figure 11:**
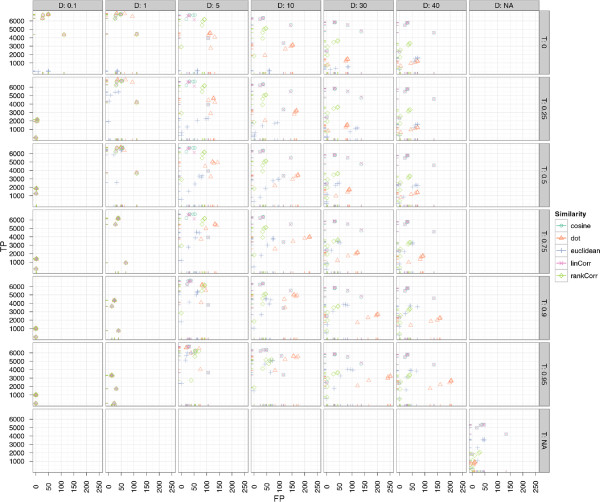
**Scatter plots for BIPACE for the wheat dataset with alignment false positives and true positives. **Scatter plots for BIPACE for the wheat dataset with alignment false positives and true positives conditioned on retention time tolerance *D* (columns) and retention time threshold *T* (rows). Instances without retention time penalized similarity function are shown in the *NA* row/column for reference. It is visible that the unpenalized instances perform consistently worse on true positives, while they perform better with regard to the number of false positives.

The results for BIPACE on the wheat dataset show very good performance in absolute and relative numbers. Figure 11 shows the absolute numbers of true positive versus false positive assignments for varying *T* (rows) and *D* (columns) parameters. The overall best result is achieved using the dot product (*dot*) for instances using the time penalty function, and the cosine (*cosine*) for instances not using the time penalty function. The instances using no additional retention time penalty are visible at the bottom left of Figure 11. These do not achieve as many true positives as the time penalized variants, however, they tend to produce fewer false positives as well. The negative Euclidean distance (*euclidean*) in combination with a time penalty, produces the fewest number of false positives, regardless of the value of *D*.

**Figure 12:**
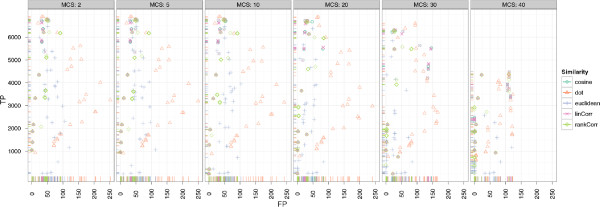
**Scatter plots for BIPACE for the wheat dataset with alignment false positives and true positives. **Scatter plots for BIPACE for the wheat dataset with alignment false positives and true positives conditioned on the minimum clique size (*MCS*) parameter (columns). The highest number of true positives is reported for the smallest possible value of *MCS *= 2. Better false positive numbers are found for higher values of *MCS* at the expense of true positives.

Figure 12 shows the dependency of true and false positives with regard to the *MCS* parameter. This parameter shows the relation of a small *MCS* value to a high number of true positives, but also to more false positives, since a larger number of small cliques with lower individual support are reported. Larger cliques have a high support for each contained peak and are thus more influential for the total number of true positives, but they occur less often, as is visible for *MCS *= 40, where each peak group must contain peaks from all 40 chromatograms. Again, as in Figure 11, dot product and cosine give the best results in absolute numbers of true and false positive assignments.

**Figure 13:**
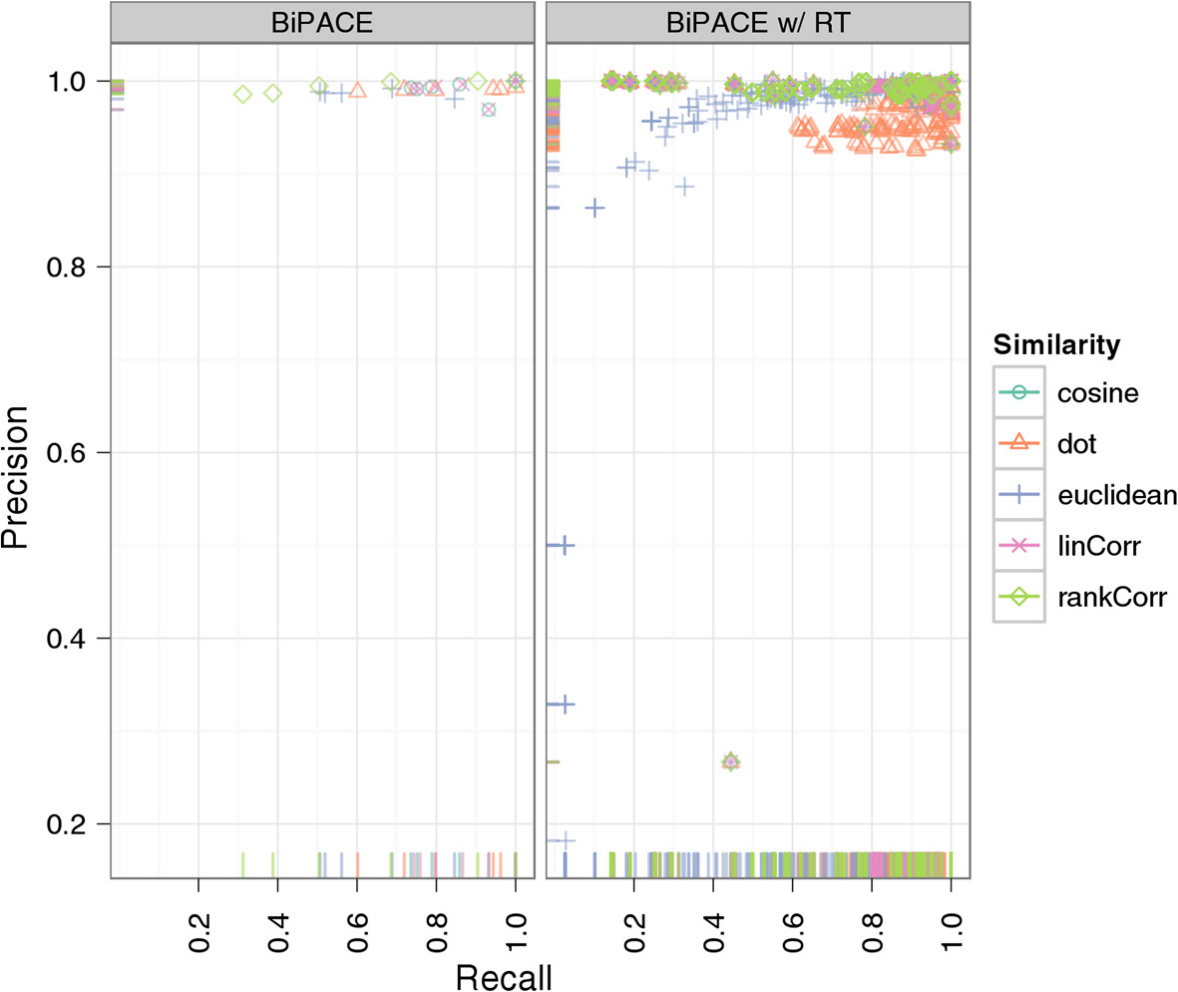
**Scatter plots for BIPACE for the wheat dataset with alignment precision and recall. **The retention time penalized variant of BIPACE using the dot product performs better than the plain variant using the cosine as the similarity function. The evaluation results of some instances report neither false positive nor false negative assignments, leading to precision and recall values of 1. These instances in general report only about one third of the maximum number of true positives reported for other parameterizations.

The precision and recall plot in Figure 13 does not clearly visualize a superior parameterization, but from Additional file 2: Table S2 we see, that the dot product is the best similarity function for retention time penalized instances with *MCS *= 10, 6891 true positives, 36 false positives, and 433 false negatives. The best parameterization without retention time penalty also used the cosine with *MCS *= 2, resulting in 5357 true positives, 39 false positives and 1924 false negatives. However, the retention time penalized variants tend to have a lower runtime, depending on the *T* parameter used.

There are no true negatives reported for the wheat evaluation, as there were no missing peak annotations in the ground truth. This explains the high number of false negatives for BIPACE, due to not completely connected peak groups, which prohibits BIPACE to form larger cliques. The peaks which could not be assigned to any cliques are consequently missing from the reported multiple alignments.

#### Results of CeMAPP-DTW

**Figure 14:**
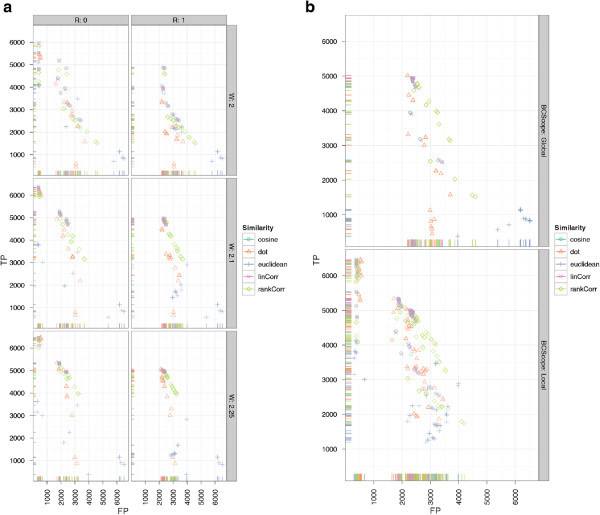
**Scatter plots for CeMAPP-DTW for the wheat dataset with alignment true positives and false positives. **SubFigure 14(a) shows alignment true positives and false positives conditioned on anchor radius (columns) and alignment match weight (rows). It is clearly visible that an anchor radius of *R *= 0 combined with a match weight of *W *= 2.25 gives the best results for linear correlation and the dot product. SubFigure 14(b) shows alignment true positives and false positives conditioned on Sakoe-Chiba bandwidth constraint *BC* as relative number of scans (columns). Rows show whether the constraint was applied globally or locally (*BCScope*). The best results were obtained for a local window of 0.1·max{|*A*|,|*B*|}.

For CeMAPP-DTW, the results are comparable to those obtained for the *Leishmania* dataset. Without the anchors defined by BIPACE, CeMAPP-DTW has fewer true positive results and more false positive results. Here, the time penalized variant of the dot product with *D *= 30 seconds, BIPACE anchors, a local Sakoe-Chiba band constraint of *BC *= 0.1, and a *matchWeight *= 2.25 achieves the best result with 6459 true positives, 387 false positives and 514 false negatives. The best result using no anchors from BIPACE uses the dot product with *D *= 1 seconds retention time penalty, a global Sakoe-Chiba band constraint of 0.1, match weight *W *= 2.25, achieving 5017 true positives, 2194 false positives and 149 false negatives. These results are illustrated in Figure 14, showing the dependencies of true and false positives on the different parameters. Figure 14(a) shows that a small anchor radius *R *= 0 combined with a match weight *W *= 2.25 yields the highest number of true positives. In Figure 14(b), the dependency between the use of a global or local (*BCScope*) Sakoe-Chiba band versus different values for the band width parameter *BC* is shown. In general, a local band of width 0.1 yields the best results.

## Discussion

The results of BIPACE and CeMAPP-DTW presented in the previous sections show the advantage of using a retention time penalty as an additional criterion together with the mass spectral similarity function. The runtime boxplots in Figures 5(a) and 10(a) show the advantage of using the *T* parameter as a threshold on the retention time penalty function. If the value of the retention time penalty function is larger than the *T*, then the costly similarity functions are applied, otherwise, the calculation is stopped immediately for that peak pair.

Therefore, tuning of the *T* parameter is one possible option to speed up the calculation of both BIPACE and CeMAPP-DTW. Since the time penalized similarity variants consistently perform better than the non-penalized ones, it is also advisable to check on the *T* parameter. Our results show that this parameter should initially be set to a rather small number, since it does not directly correspond to the expected retention time deviation. Finally, the minimum clique size *MCS* is an important parameter for BIPACE and influences the number of cliques that are reported in the multiple alignment. Using a high value for *MCS* returns only those cliques whose peaks are all bidirectional best hits of each other and thus support each other as members of the clique. Lower values for *MCS* return more cliques, but at the expense of returning a higher number of smaller cliques with potentially more misaligned peaks.

CeMAPP-DTW on the other hand has a few other parameters to tune. Our results show that the most important ones are the use of anchors and an anchor radius of 0, meaning that the DTW alignment must pass through the anchor positions for example defined by BIPACE . Additionally, the use of a local Sakoe-Chiba band constraint and a match weight of 2.25 are beneficial for the number of true positives CeMAPP-DTW is able to achieve.

Concerning the best similarity function to use, there is no decisive answer possible from our results. In accordance with [8], Pearson’s linear correlation and Spearman’s rank correlation give good results in terms of low false positive numbers, but time penalized dot product and cosine tend to give significantly higher true positive numbers. Using the time penalty function as a pre-filter for the actual similarity function seems to reduce the differences of the individual similarity functions. However, the instances using a correlation-based similarity have a significantly longer runtime (Figures 5 and 10), than the ones using the dot product or cosine similarity.

## Conclusions

We have introduced a fast and accurate method for multiple peak alignment of GC-MS data, BIPACE, that is capable of finding groups of similar peaks between chromatograms from different experimental groups (e.g. treatment and control), achieving a high number of true positive and a very low number of false positive assignments. Our method achieves results comparable to that of Robinson *et al.*[10], while being easily tunable to a very low false positive rate via the minimum clique size parameter.

With the use of the peak groups aligned by BIPACE as anchors within partitioned DTW, we address one major issue of similar profile-alignment algorithms, namely their quadratic time and space complexity by partitioning the pairwise alignment matrix into adjacent regions. Thus, strong peak candidates, such as reference compounds with unique mass traces (LC-MS) or characteristic fragmentation patterns (GC-MS) are definitely aligned, while weaker peaks that were not discovered during peak finding are also aligned, but with more flexibility.

We have shown that the partitioned DTW algorithm used in CeMAPP-DTW on its own is able to calculate a profile-based multiple alignment in less time and with fewer space requirements when compared to unconstrained DTW. Combining CeMAPP-DTW with the aligned peak groups from BIPACE as alignment anchors allowed us to improve both on the runtime, as well as on the number of true positives recovered by the alignment. This combination of the two algorithms is feasible if a definite alignment is not the main requirement, but instead the output of CeMAPP-DTW is used for a subsequent retention time correction of the profile data. For a definite multiple peak alignment BIPACE is the better alternative.

## Competing interests

The authors declare that they have no competing interests.

## Authors’ contributions

NH designed and implemented BIPACE and CeMAPP-DTW and drafted the manuscript. MK, PH and KN provided the biological background and data for the wheat dataset evaluation. MW helped with the implementation and testing of BIPACE and CeMAPP-DTW. HN reviewed and contributed to CeMAPP-DTW and provided the reference data for the wheat dataset evaluation. JS suggested the bidirectional best hits approach and reviewed the BIPACE and CeMAPP-DTW algorithms. All authors read and approved of the final manuscript.

## Supplementary Material

Additional file 1 **Archive containing evaluation tables for the*Leishmania*parasite dataset.** The complete evaluation table giving the parameters and classification results for BIPACE, CeMAPP-DTW and Robinson’s [10] method for the *Leishmania* parasite dataset is contained in a zip-archive along with the corresponding figures. Table S1 in the manuscript corresponds to the file ‘evaluation.csv’ in this archive. Click here for file

Additional file 2 **Archive containing evaluation tables for the wheat dataset.** The complete evaluation table giving the parameters and classification results for BIPACE and CeMAPP-DTW for the Wheat dataset is contained in a zip-archive along with the corresponding figures. Table S2 in the manuscript corresponds to the file ‘evaluation.csv’ in this archive. The corresponding raw dataset together with experimental parameters, peak lists and reference multiple peak alignment is available from the Metabolights database at http://www.ebi.ac.uk/metabolights/MTBLS21. Click here for file
