# Supplementary figures and images for: Combining peak- and chromatogram-based retention time alignment algorithms for multiple chromatography-mass spectrometry datasets
Source: BMC Bioinformatics. 2012 Aug 27;13:214. doi: 10.1186/1471-2105-13-214 (PMC3546004; doi:10.1186/1471-2105-13-214)

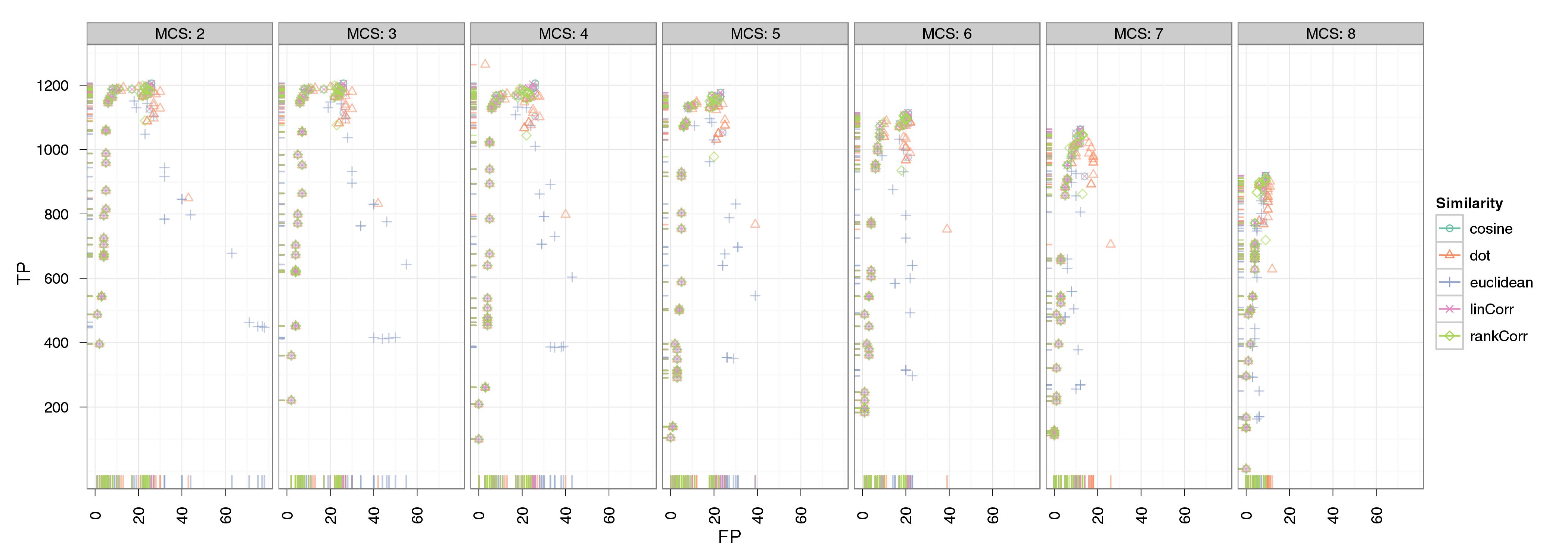

Supplement: Additional file 1 — Archive containing evaluation tables for theLeishmaniaparasite dataset. The complete evaluation table giving the parameters and classification results for BIPACE, CeMAPP-DTW and Robinson’s [10] method for the Leishmania parasite dataset is contained in a zip-archive along with the corresponding figures. Table S1 in the manuscript corresponds to the file ‘evaluation.csv’ in this archive. [file 1471-2105-13-214-S1.zip › leishmania/bipace-fp-vs-tp-mcs.png]

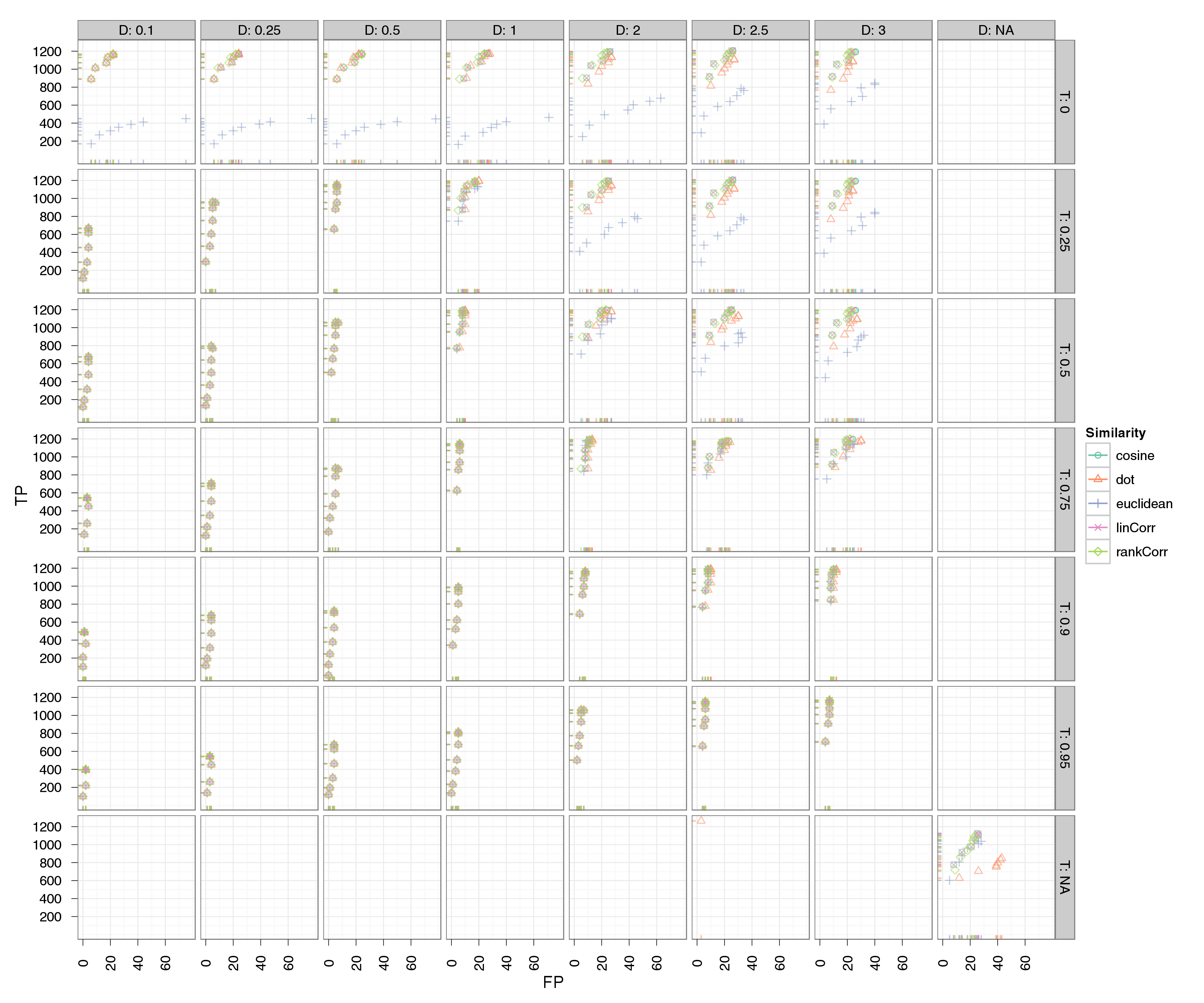

Supplement: Additional file 1 — Archive containing evaluation tables for theLeishmaniaparasite dataset. The complete evaluation table giving the parameters and classification results for BIPACE, CeMAPP-DTW and Robinson’s [10] method for the Leishmania parasite dataset is contained in a zip-archive along with the corresponding figures. Table S1 in the manuscript corresponds to the file ‘evaluation.csv’ in this archive. [file 1471-2105-13-214-S1.zip › leishmania/bipace-fp-vs-tp-rtThres-rtTol.png]

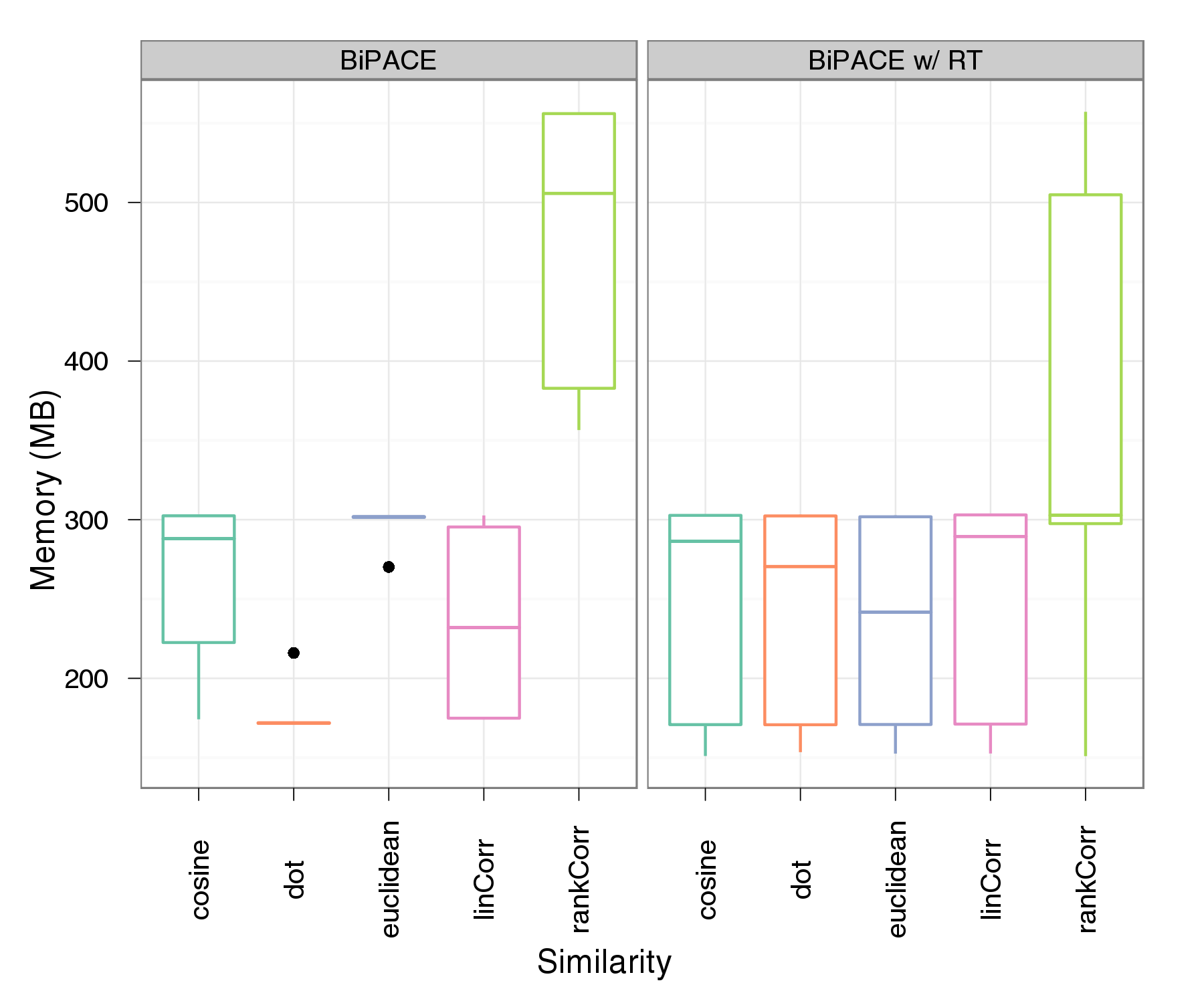

Supplement: Additional file 1 — Archive containing evaluation tables for theLeishmaniaparasite dataset. The complete evaluation table giving the parameters and classification results for BIPACE, CeMAPP-DTW and Robinson’s [10] method for the Leishmania parasite dataset is contained in a zip-archive along with the corresponding figures. Table S1 in the manuscript corresponds to the file ‘evaluation.csv’ in this archive. [file 1471-2105-13-214-S1.zip › leishmania/bipace-memory-histogram.png]

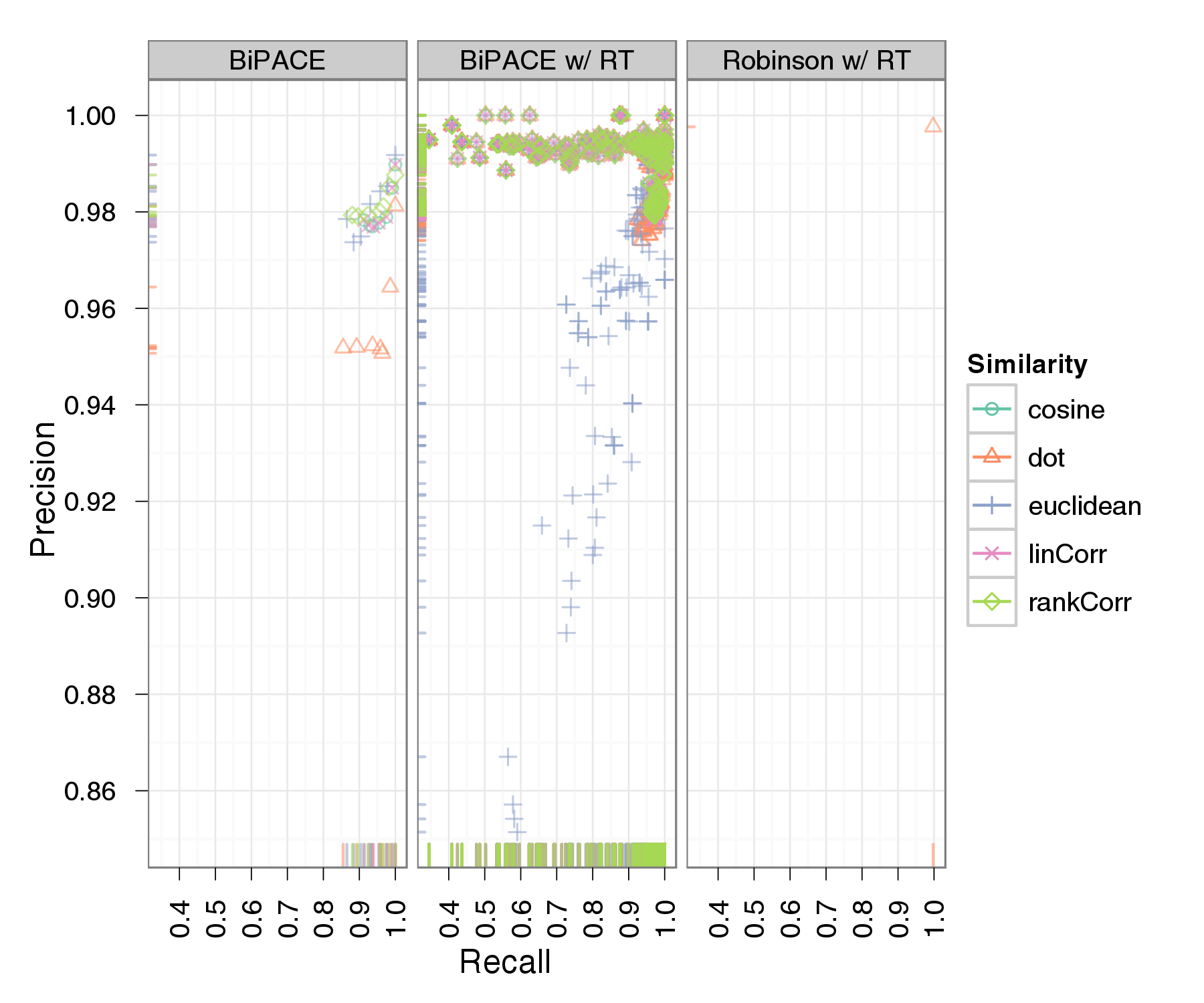

Supplement: Additional file 1 — Archive containing evaluation tables for theLeishmaniaparasite dataset. The complete evaluation table giving the parameters and classification results for BIPACE, CeMAPP-DTW and Robinson’s [10] method for the Leishmania parasite dataset is contained in a zip-archive along with the corresponding figures. Table S1 in the manuscript corresponds to the file ‘evaluation.csv’ in this archive. [file 1471-2105-13-214-S1.zip › leishmania/bipace-recall-vs-precision.png]

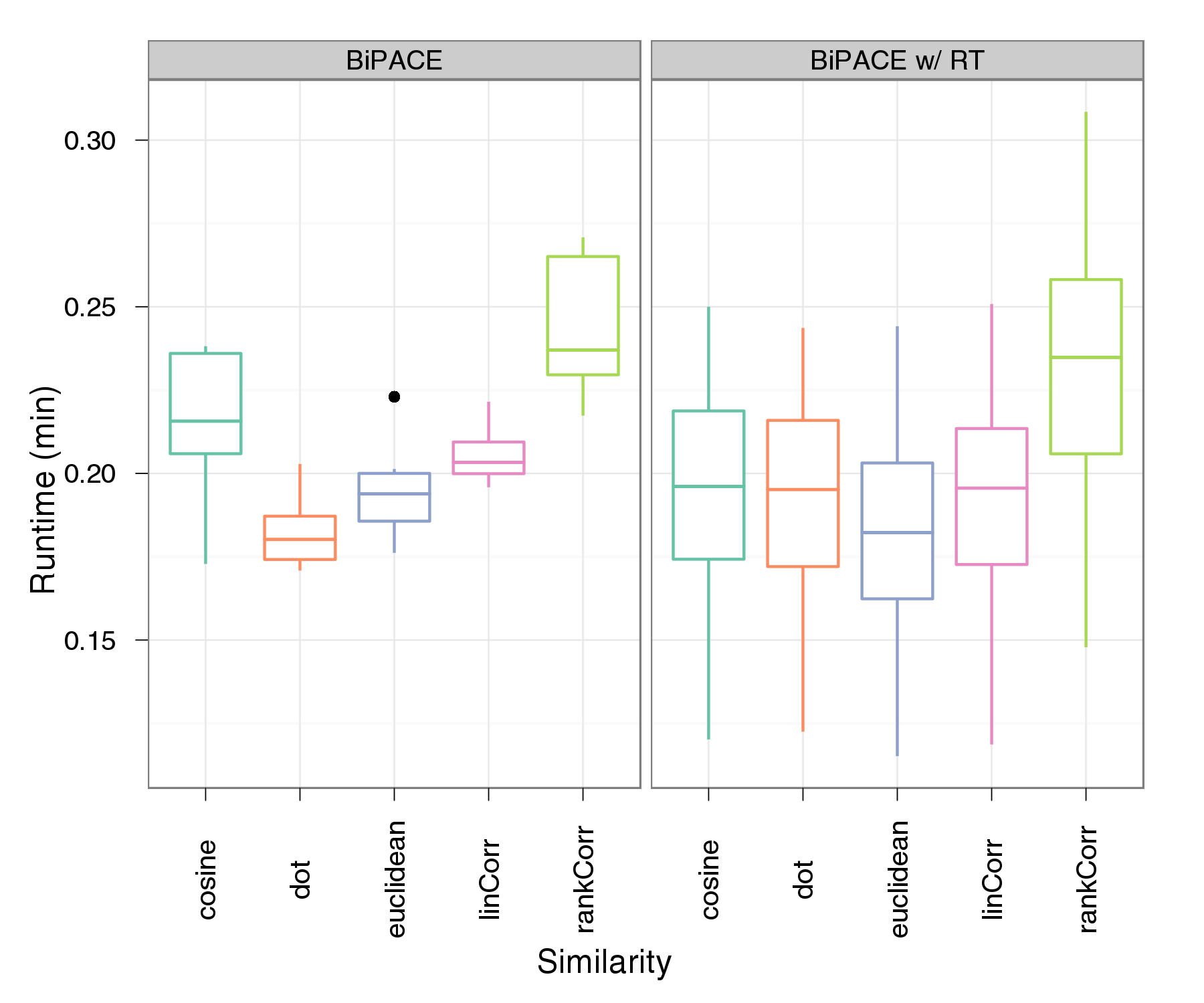

Supplement: Additional file 1 — Archive containing evaluation tables for theLeishmaniaparasite dataset. The complete evaluation table giving the parameters and classification results for BIPACE, CeMAPP-DTW and Robinson’s [10] method for the Leishmania parasite dataset is contained in a zip-archive along with the corresponding figures. Table S1 in the manuscript corresponds to the file ‘evaluation.csv’ in this archive. [file 1471-2105-13-214-S1.zip › leishmania/bipace-runtime-histogram.png]

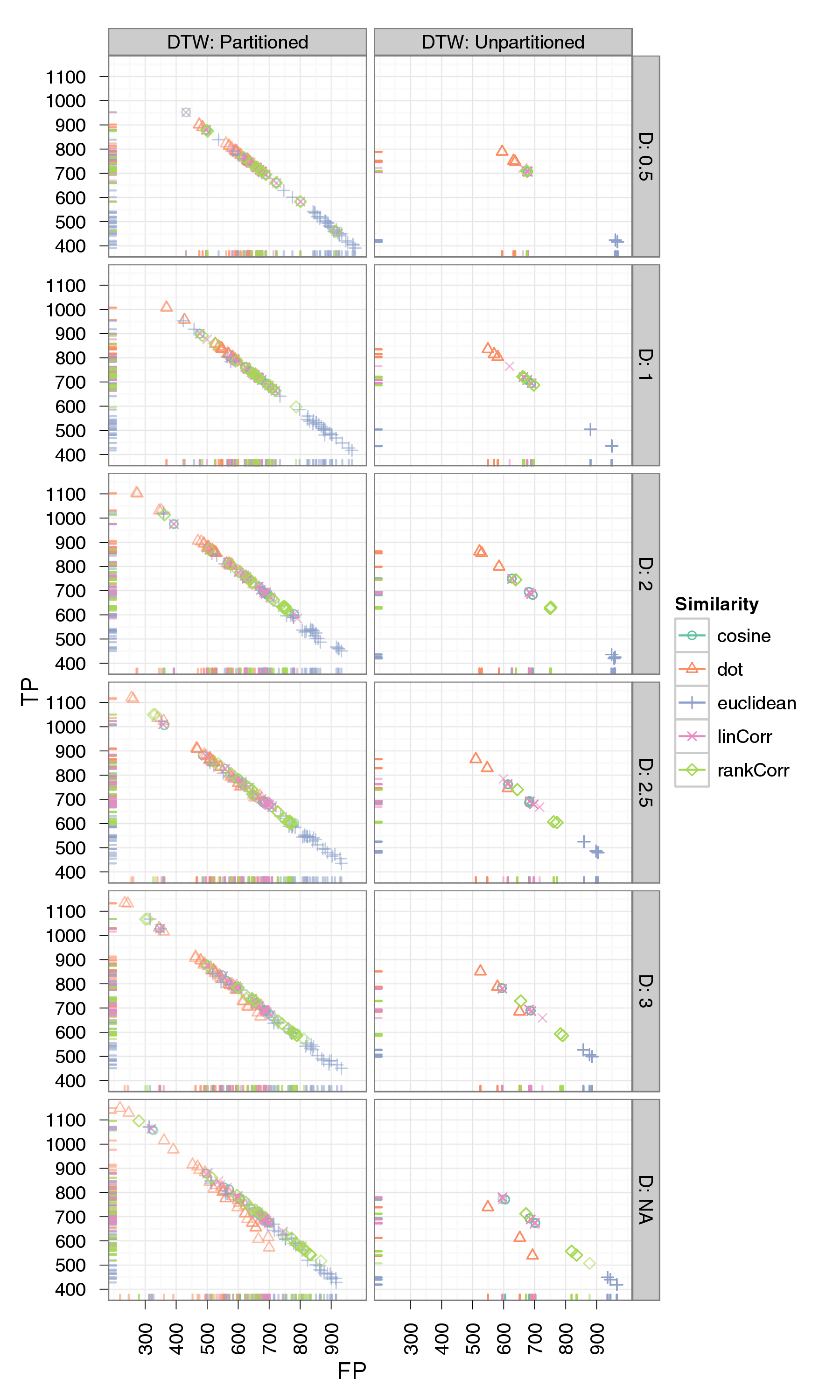

Supplement: Additional file 1 — Archive containing evaluation tables for theLeishmaniaparasite dataset. The complete evaluation table giving the parameters and classification results for BIPACE, CeMAPP-DTW and Robinson’s [10] method for the Leishmania parasite dataset is contained in a zip-archive along with the corresponding figures. Table S1 in the manuscript corresponds to the file ‘evaluation.csv’ in this archive. [file 1471-2105-13-214-S1.zip › leishmania/cemapp-fp-vs-tp-anchor-rtTol.png]

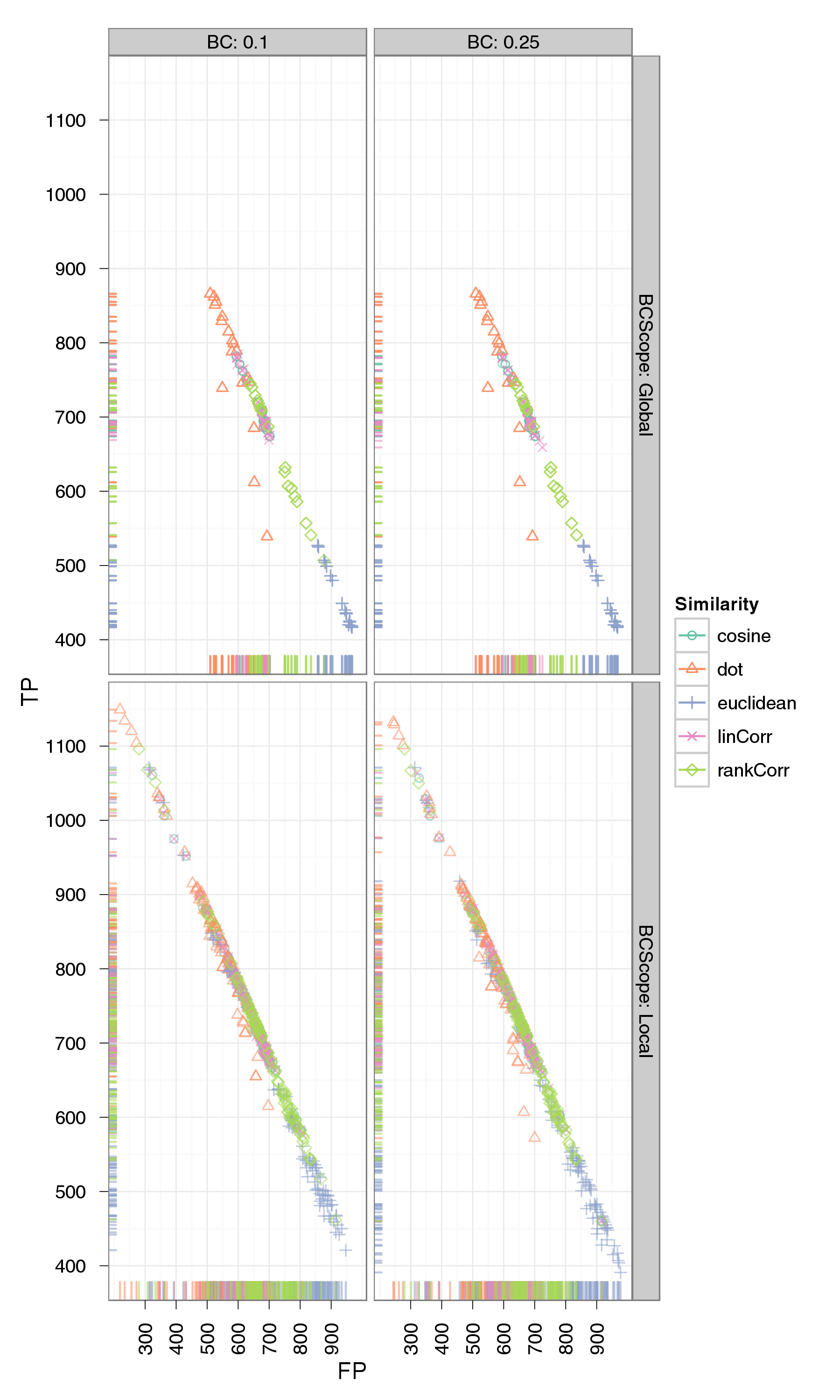

Supplement: Additional file 1 — Archive containing evaluation tables for theLeishmaniaparasite dataset. The complete evaluation table giving the parameters and classification results for BIPACE, CeMAPP-DTW and Robinson’s [10] method for the Leishmania parasite dataset is contained in a zip-archive along with the corresponding figures. Table S1 in the manuscript corresponds to the file ‘evaluation.csv’ in this archive. [file 1471-2105-13-214-S1.zip › leishmania/cemapp-fp-vs-tp-globalBand-BW.png]

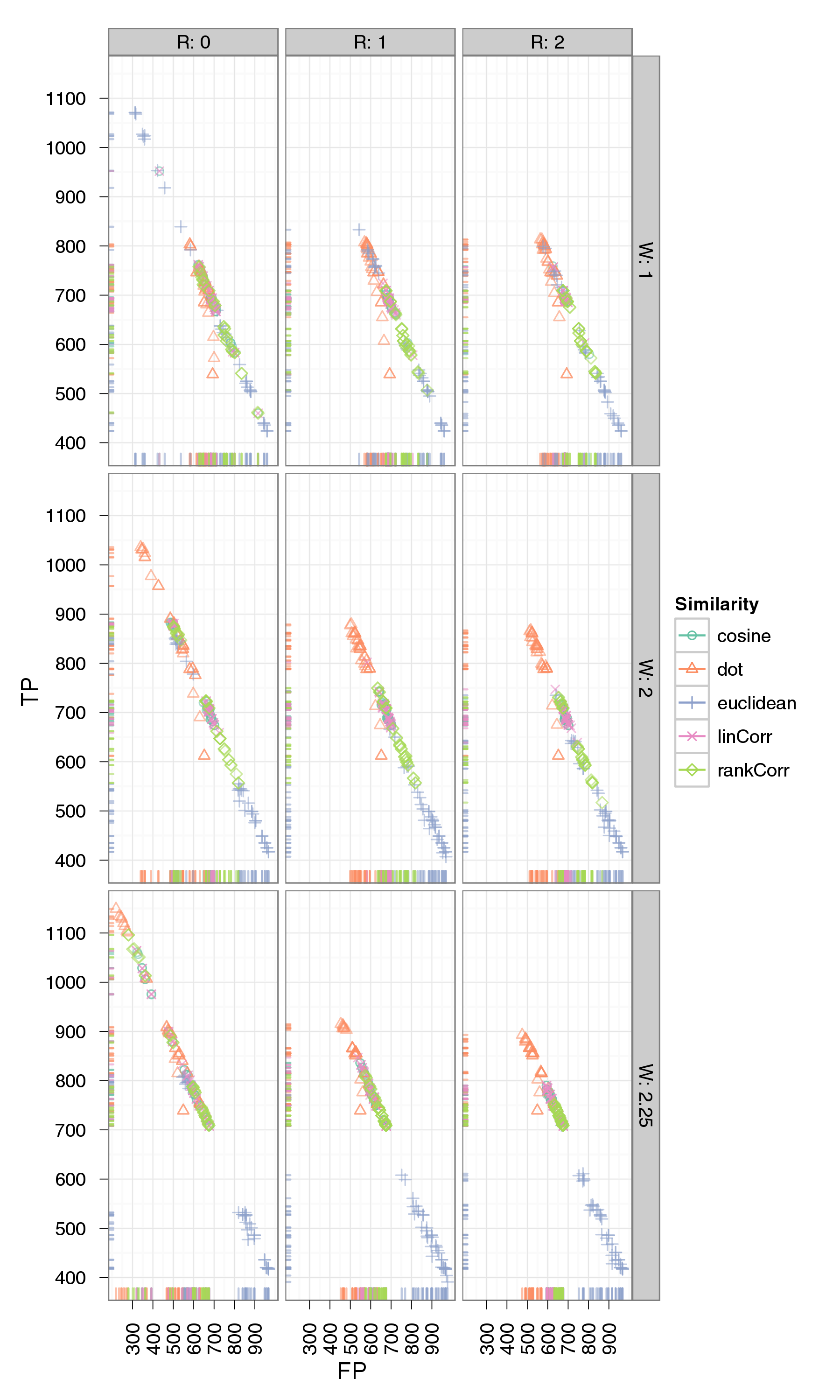

Supplement: Additional file 1 — Archive containing evaluation tables for theLeishmaniaparasite dataset. The complete evaluation table giving the parameters and classification results for BIPACE, CeMAPP-DTW and Robinson’s [10] method for the Leishmania parasite dataset is contained in a zip-archive along with the corresponding figures. Table S1 in the manuscript corresponds to the file ‘evaluation.csv’ in this archive. [file 1471-2105-13-214-S1.zip › leishmania/cemapp-fp-vs-tp-matchWeight-anchorRadius.png]

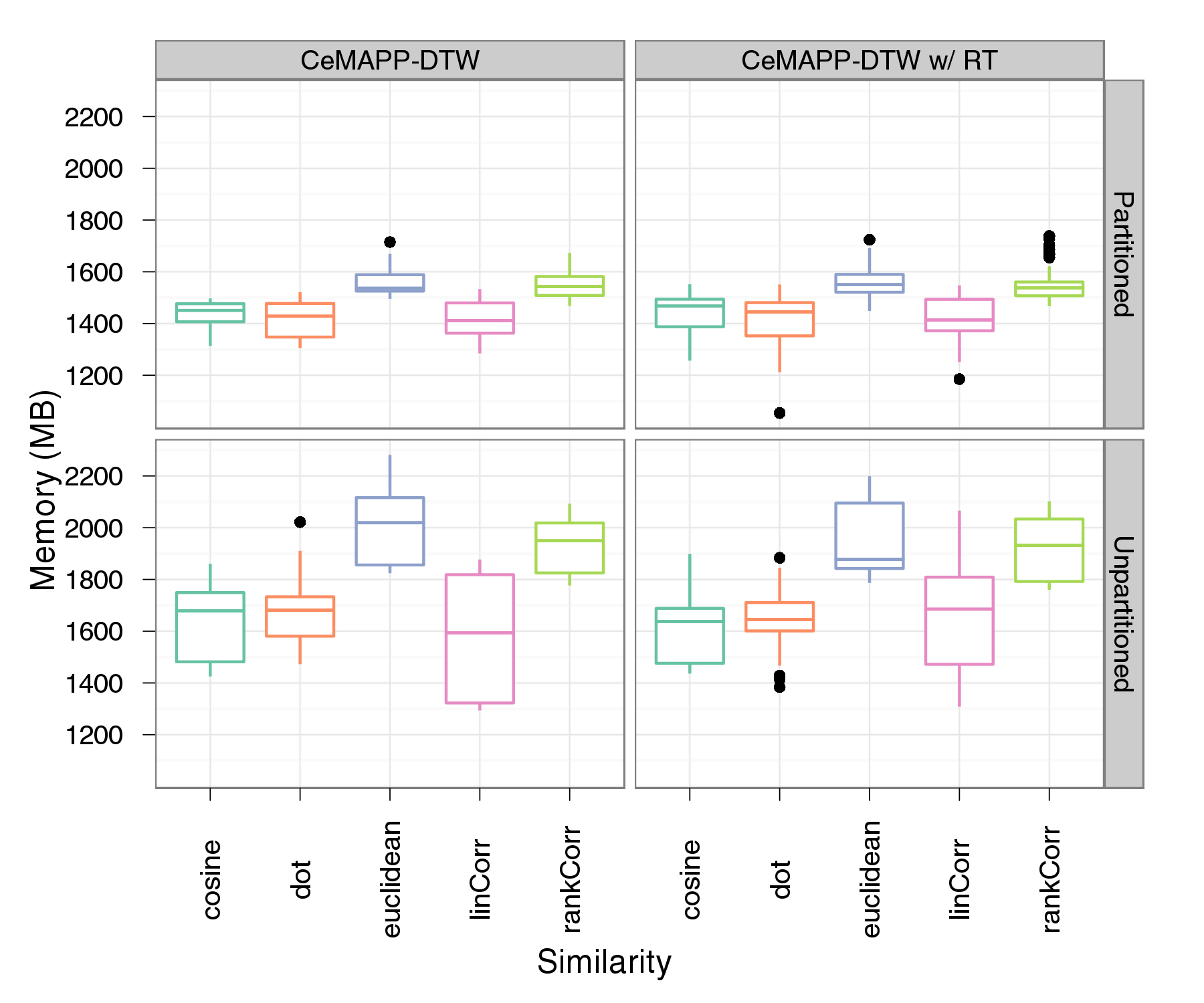

Supplement: Additional file 1 — Archive containing evaluation tables for theLeishmaniaparasite dataset. The complete evaluation table giving the parameters and classification results for BIPACE, CeMAPP-DTW and Robinson’s [10] method for the Leishmania parasite dataset is contained in a zip-archive along with the corresponding figures. Table S1 in the manuscript corresponds to the file ‘evaluation.csv’ in this archive. [file 1471-2105-13-214-S1.zip › leishmania/cemapp-memory-histogram.png]

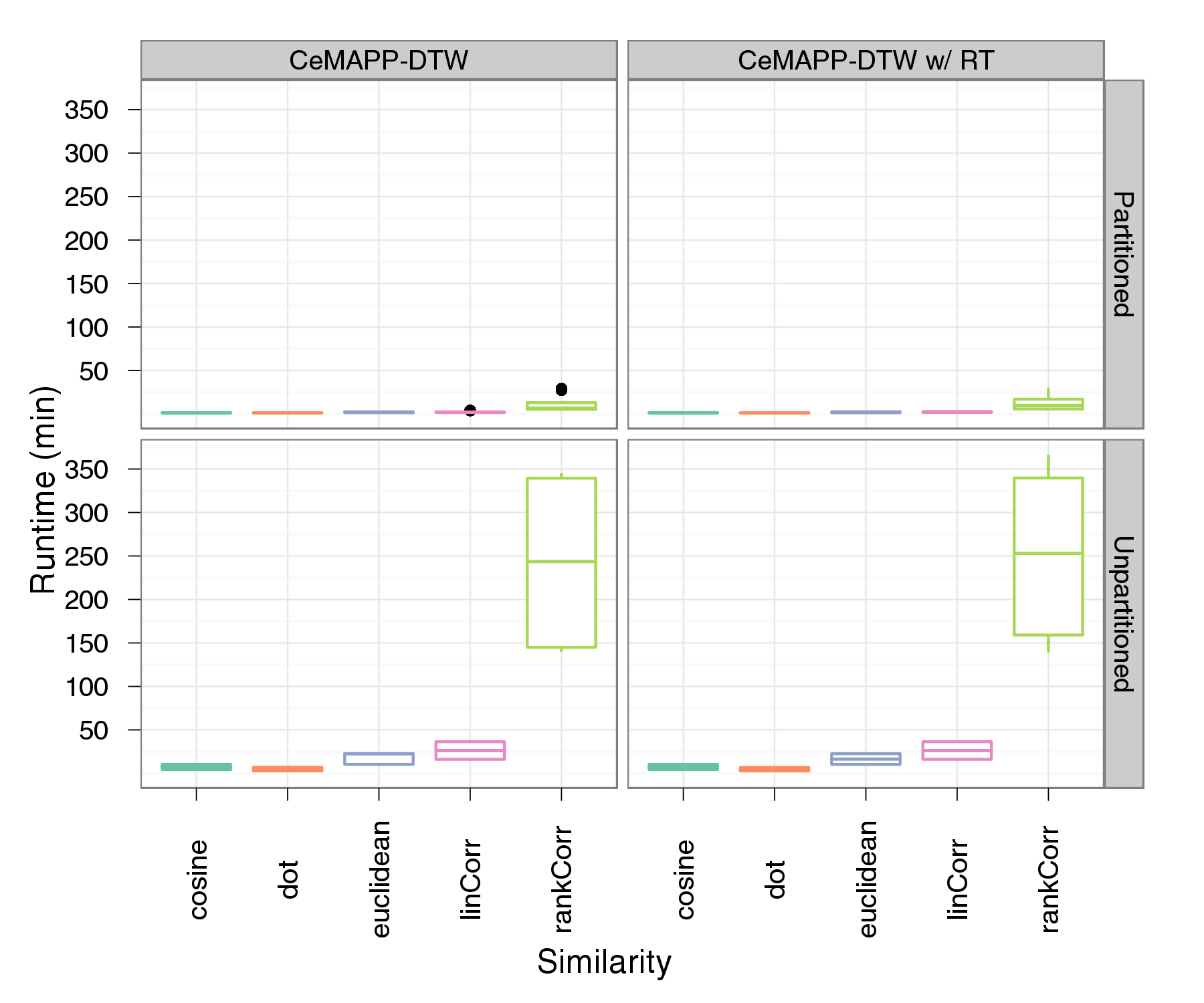

Supplement: Additional file 1 — Archive containing evaluation tables for theLeishmaniaparasite dataset. The complete evaluation table giving the parameters and classification results for BIPACE, CeMAPP-DTW and Robinson’s [10] method for the Leishmania parasite dataset is contained in a zip-archive along with the corresponding figures. Table S1 in the manuscript corresponds to the file ‘evaluation.csv’ in this archive. [file 1471-2105-13-214-S1.zip › leishmania/cemapp-runtime-histogram.png]

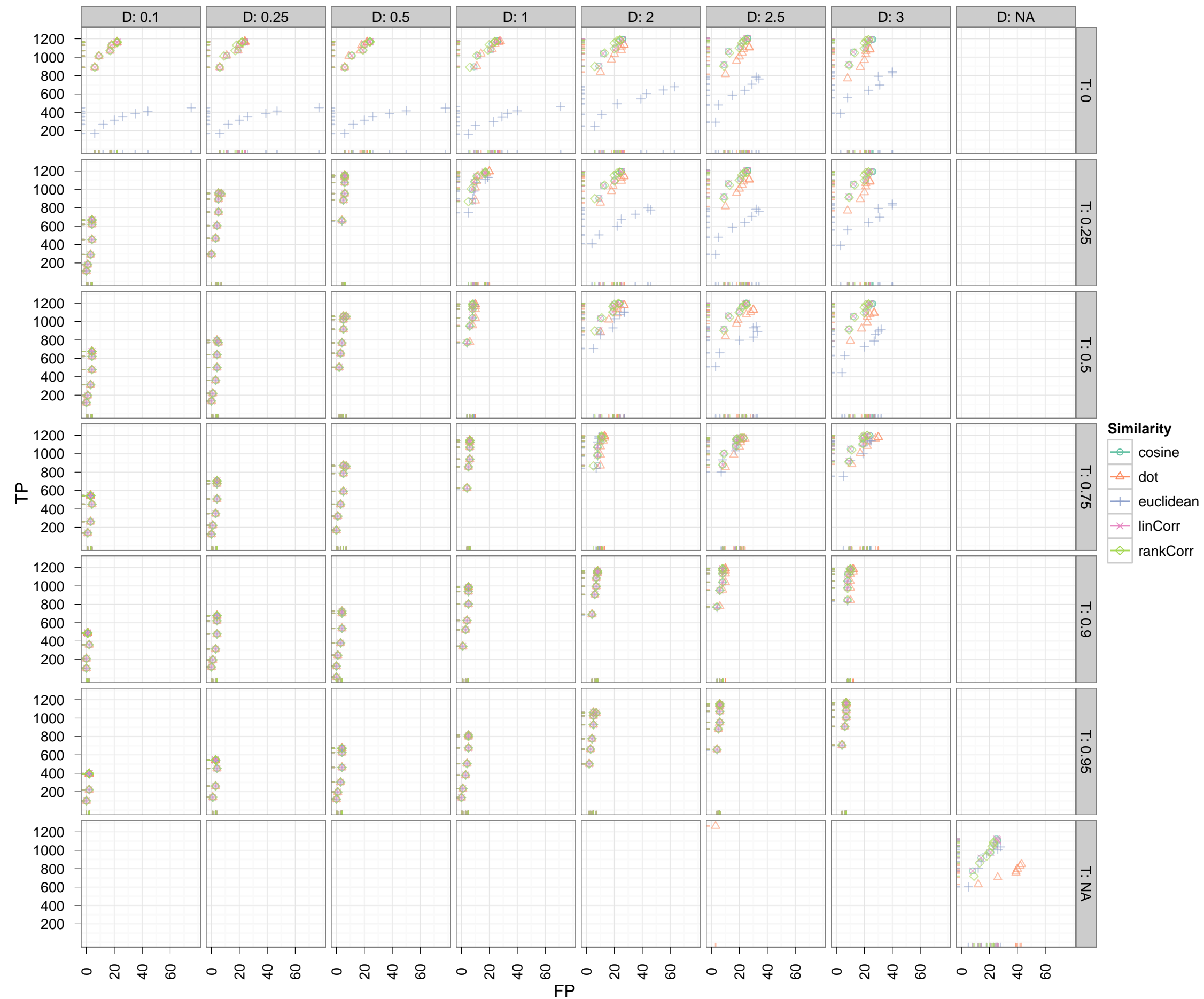

Supplement: Additional file 1 — Archive containing evaluation tables for theLeishmaniaparasite dataset. The complete evaluation table giving the parameters and classification results for BIPACE, CeMAPP-DTW and Robinson’s [10] method for the Leishmania parasite dataset is contained in a zip-archive along with the corresponding figures. Table S1 in the manuscript corresponds to the file ‘evaluation.csv’ in this archive. [file 1471-2105-13-214-S1.zip › leishmania/bipace-fp-vs-tp-rtThres-rtTol.pdf]

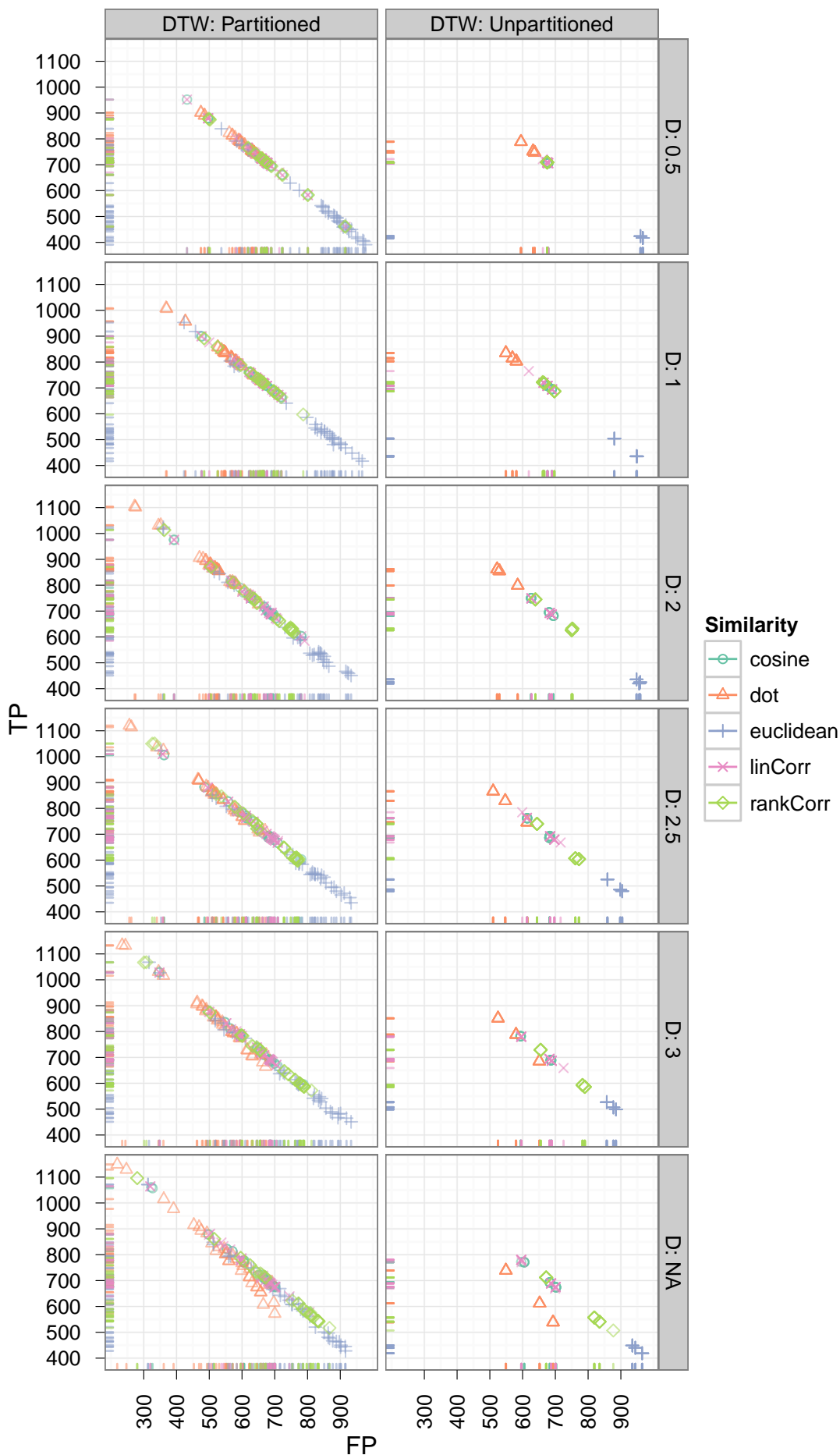

Supplement: Additional file 1 — Archive containing evaluation tables for theLeishmaniaparasite dataset. The complete evaluation table giving the parameters and classification results for BIPACE, CeMAPP-DTW and Robinson’s [10] method for the Leishmania parasite dataset is contained in a zip-archive along with the corresponding figures. Table S1 in the manuscript corresponds to the file ‘evaluation.csv’ in this archive. [file 1471-2105-13-214-S1.zip › leishmania/cemapp-fp-vs-tp-anchor-rtTol.pdf]

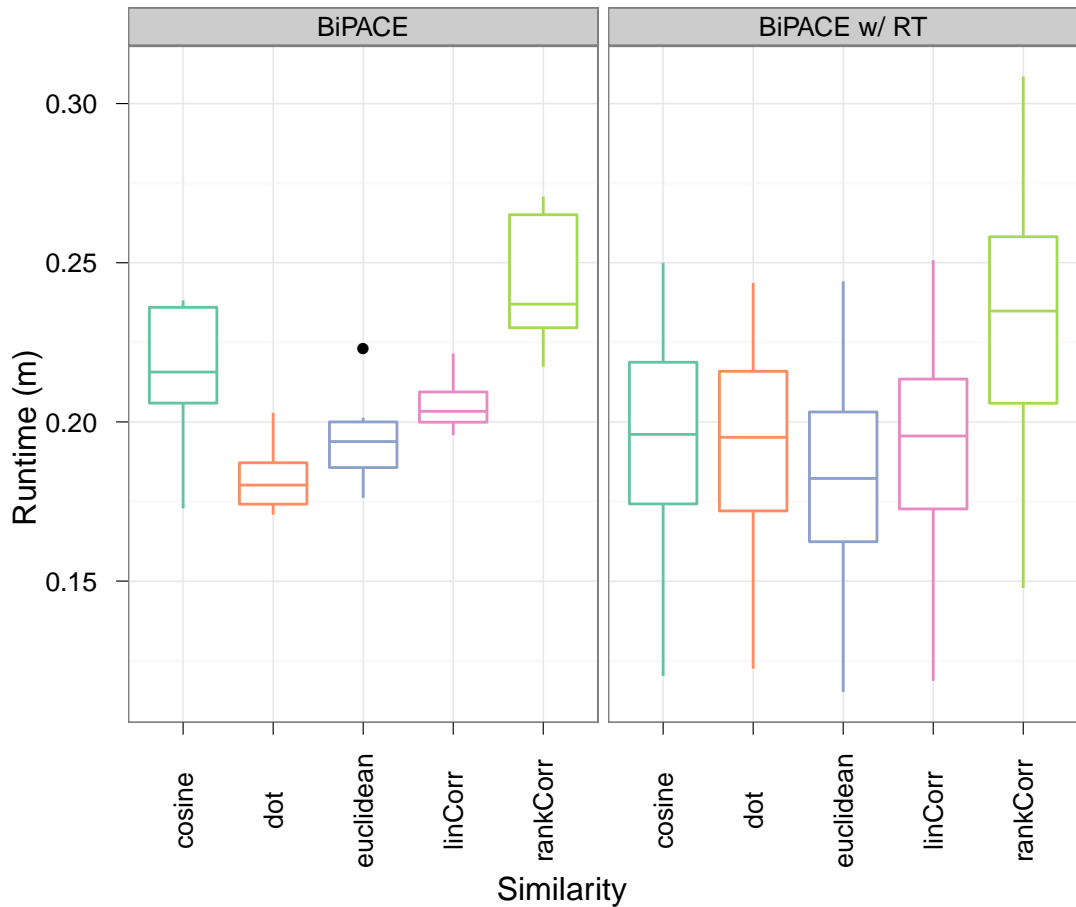

Supplement: Additional file 1 — Archive containing evaluation tables for theLeishmaniaparasite dataset. The complete evaluation table giving the parameters and classification results for BIPACE, CeMAPP-DTW and Robinson’s [10] method for the Leishmania parasite dataset is contained in a zip-archive along with the corresponding figures. Table S1 in the manuscript corresponds to the file ‘evaluation.csv’ in this archive. [file 1471-2105-13-214-S1.zip › leishmania/bipace-runtime-histogram.pdf]

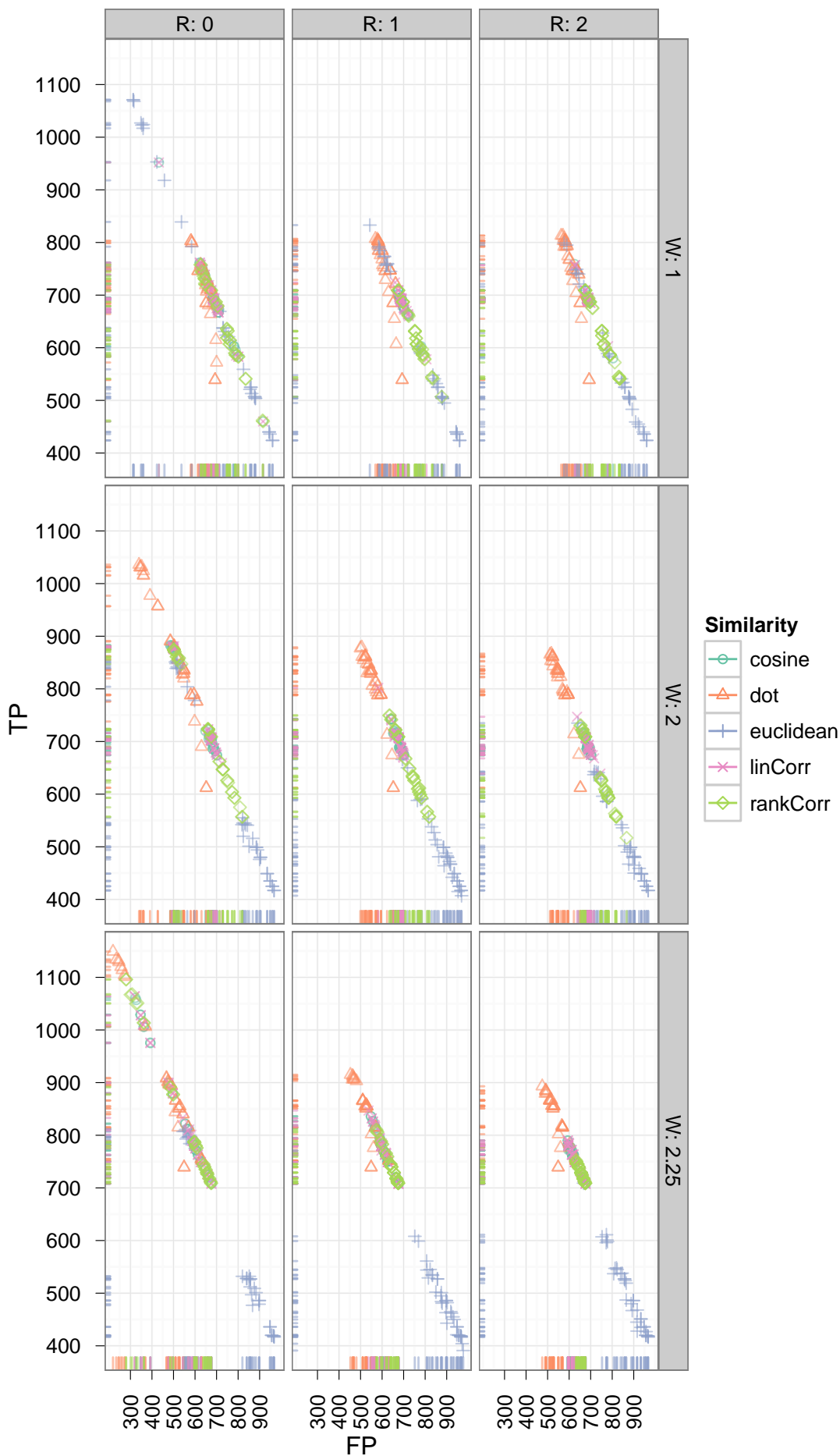

Supplement: Additional file 1 — Archive containing evaluation tables for theLeishmaniaparasite dataset. The complete evaluation table giving the parameters and classification results for BIPACE, CeMAPP-DTW and Robinson’s [10] method for the Leishmania parasite dataset is contained in a zip-archive along with the corresponding figures. Table S1 in the manuscript corresponds to the file ‘evaluation.csv’ in this archive. [file 1471-2105-13-214-S1.zip › leishmania/cemapp-fp-vs-tp-matchWeight-anchorRadius.pdf]

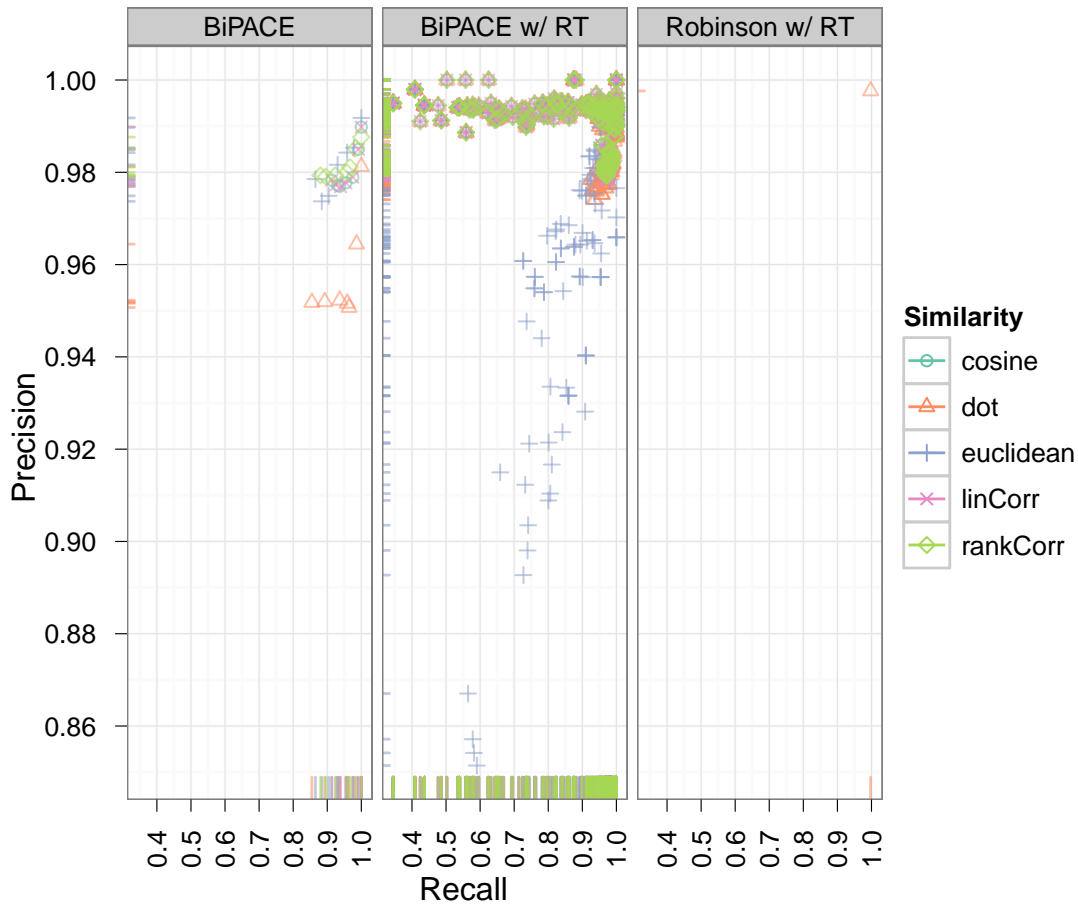

Supplement: Additional file 1 — Archive containing evaluation tables for theLeishmaniaparasite dataset. The complete evaluation table giving the parameters and classification results for BIPACE, CeMAPP-DTW and Robinson’s [10] method for the Leishmania parasite dataset is contained in a zip-archive along with the corresponding figures. Table S1 in the manuscript corresponds to the file ‘evaluation.csv’ in this archive. [file 1471-2105-13-214-S1.zip › leishmania/bipace-recall-vs-precision.pdf]

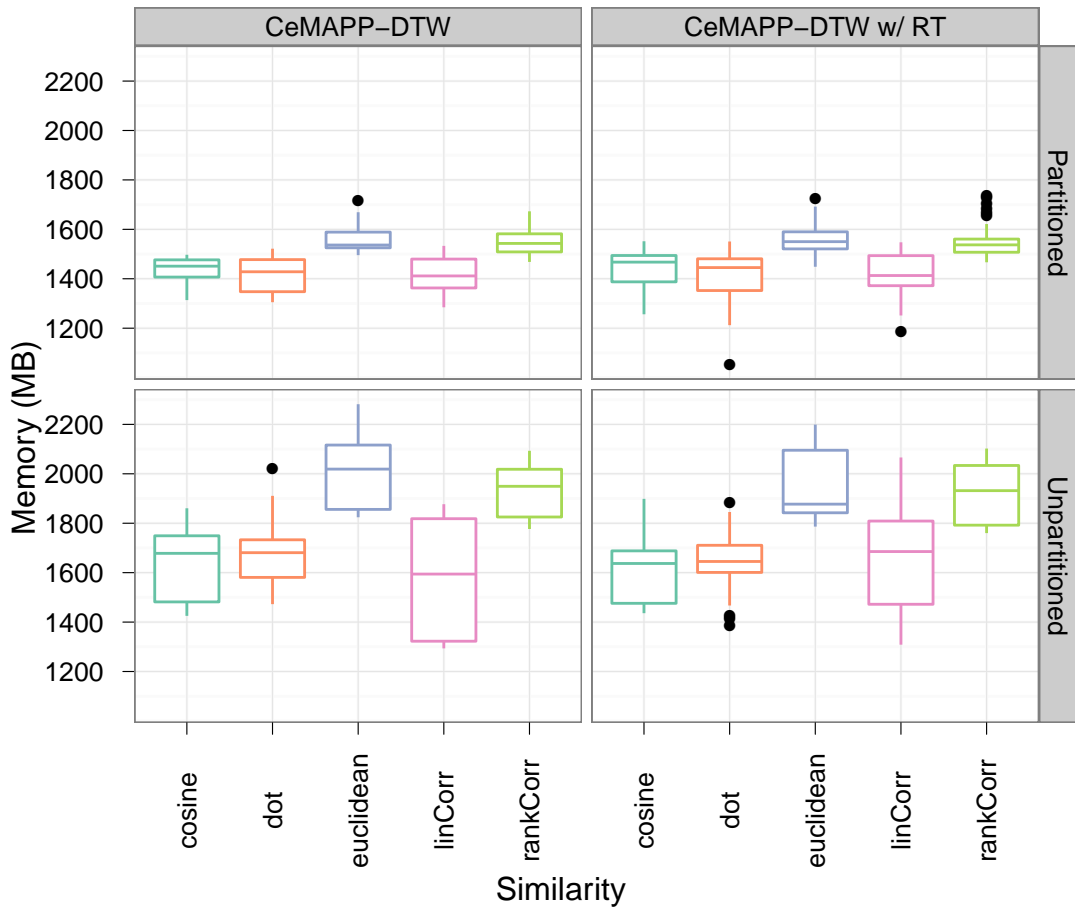

Supplement: Additional file 1 — Archive containing evaluation tables for theLeishmaniaparasite dataset. The complete evaluation table giving the parameters and classification results for BIPACE, CeMAPP-DTW and Robinson’s [10] method for the Leishmania parasite dataset is contained in a zip-archive along with the corresponding figures. Table S1 in the manuscript corresponds to the file ‘evaluation.csv’ in this archive. [file 1471-2105-13-214-S1.zip › leishmania/cemapp-memory-histogram.pdf]

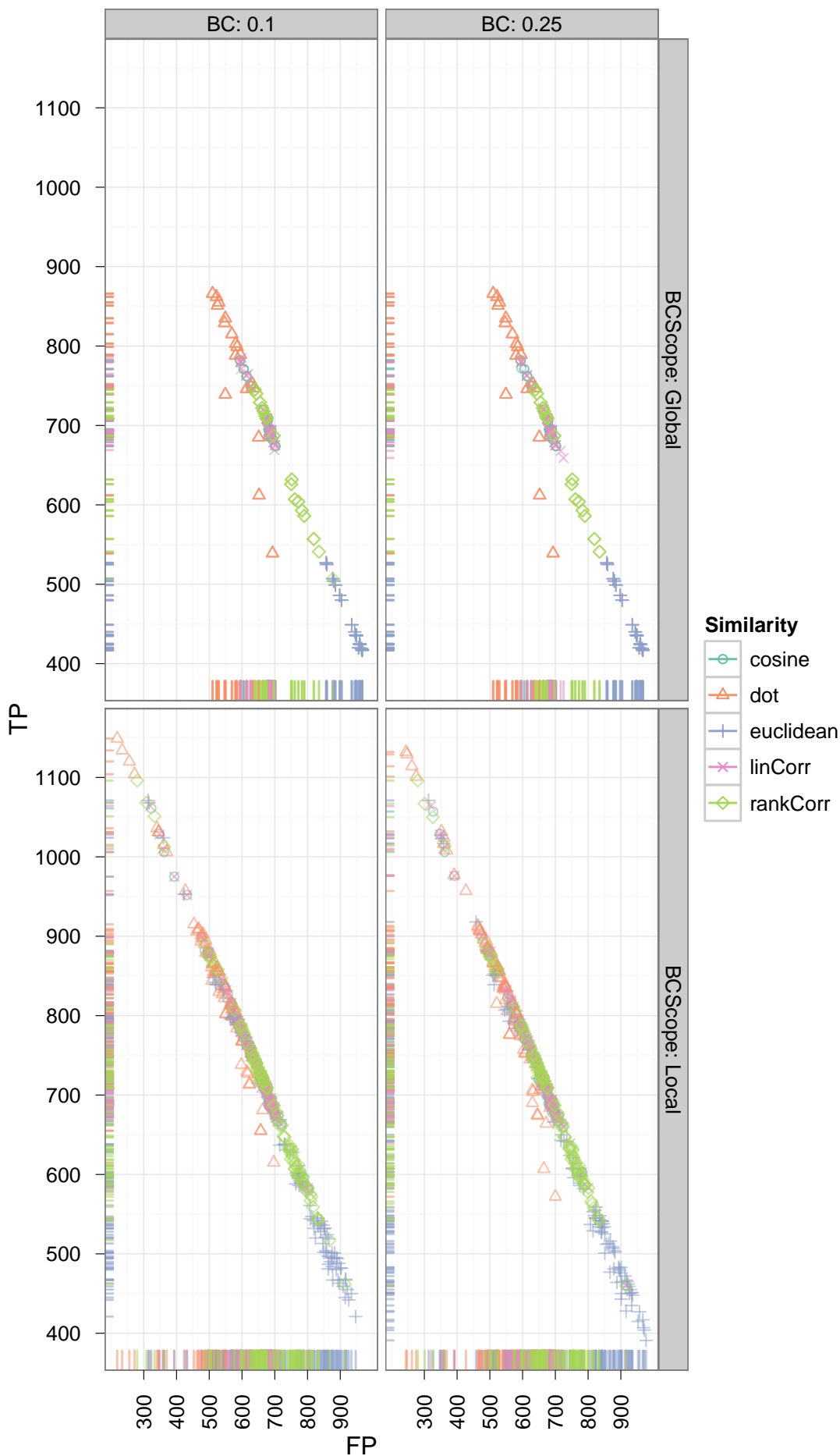

Supplement: Additional file 1 — Archive containing evaluation tables for theLeishmaniaparasite dataset. The complete evaluation table giving the parameters and classification results for BIPACE, CeMAPP-DTW and Robinson’s [10] method for the Leishmania parasite dataset is contained in a zip-archive along with the corresponding figures. Table S1 in the manuscript corresponds to the file ‘evaluation.csv’ in this archive. [file 1471-2105-13-214-S1.zip › leishmania/cemapp-fp-vs-tp-globalBand-BW.pdf]

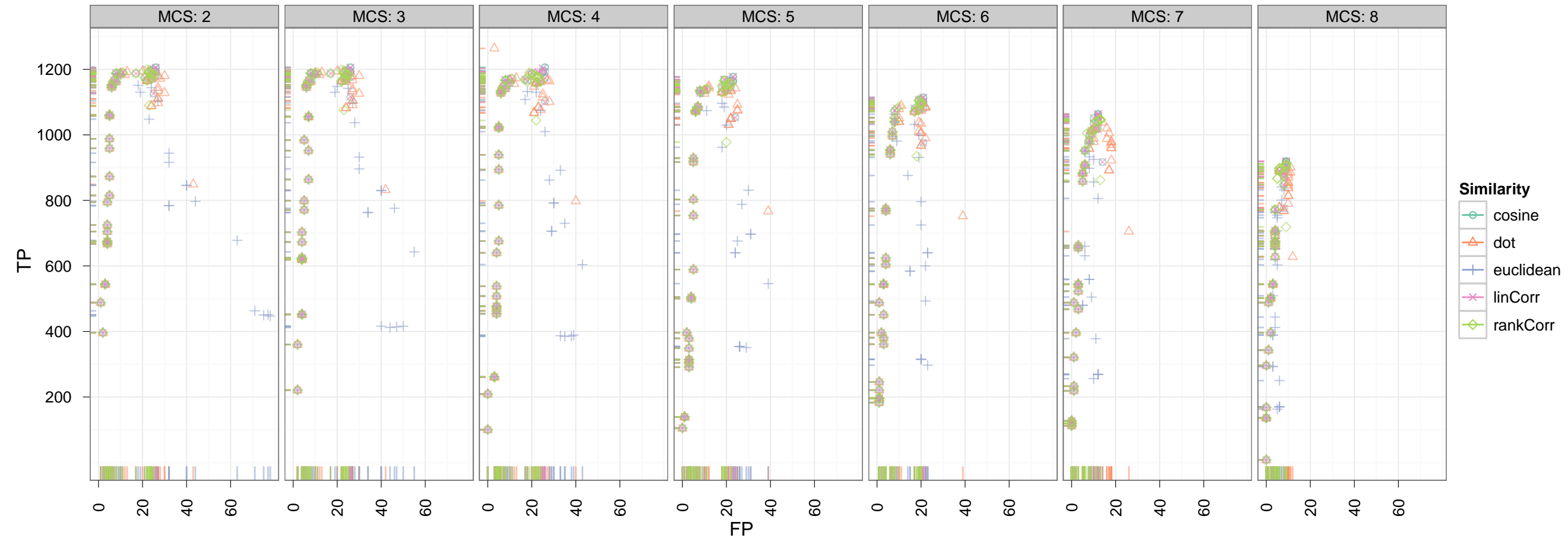

Supplement: Additional file 1 — Archive containing evaluation tables for theLeishmaniaparasite dataset. The complete evaluation table giving the parameters and classification results for BIPACE, CeMAPP-DTW and Robinson’s [10] method for the Leishmania parasite dataset is contained in a zip-archive along with the corresponding figures. Table S1 in the manuscript corresponds to the file ‘evaluation.csv’ in this archive. [file 1471-2105-13-214-S1.zip › leishmania/bipace-fp-vs-tp-mcs.pdf]

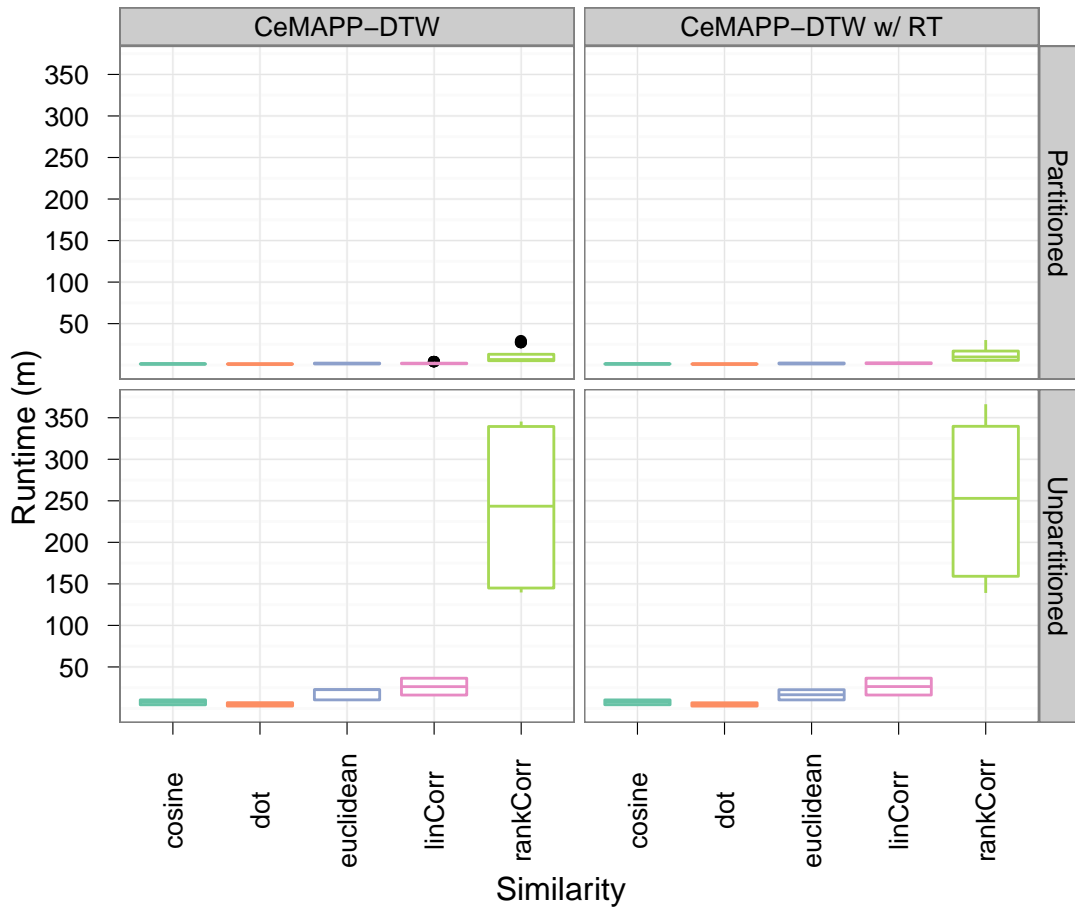

Supplement: Additional file 1 — Archive containing evaluation tables for theLeishmaniaparasite dataset. The complete evaluation table giving the parameters and classification results for BIPACE, CeMAPP-DTW and Robinson’s [10] method for the Leishmania parasite dataset is contained in a zip-archive along with the corresponding figures. Table S1 in the manuscript corresponds to the file ‘evaluation.csv’ in this archive. [file 1471-2105-13-214-S1.zip › leishmania/cemapp-runtime-histogram.pdf]

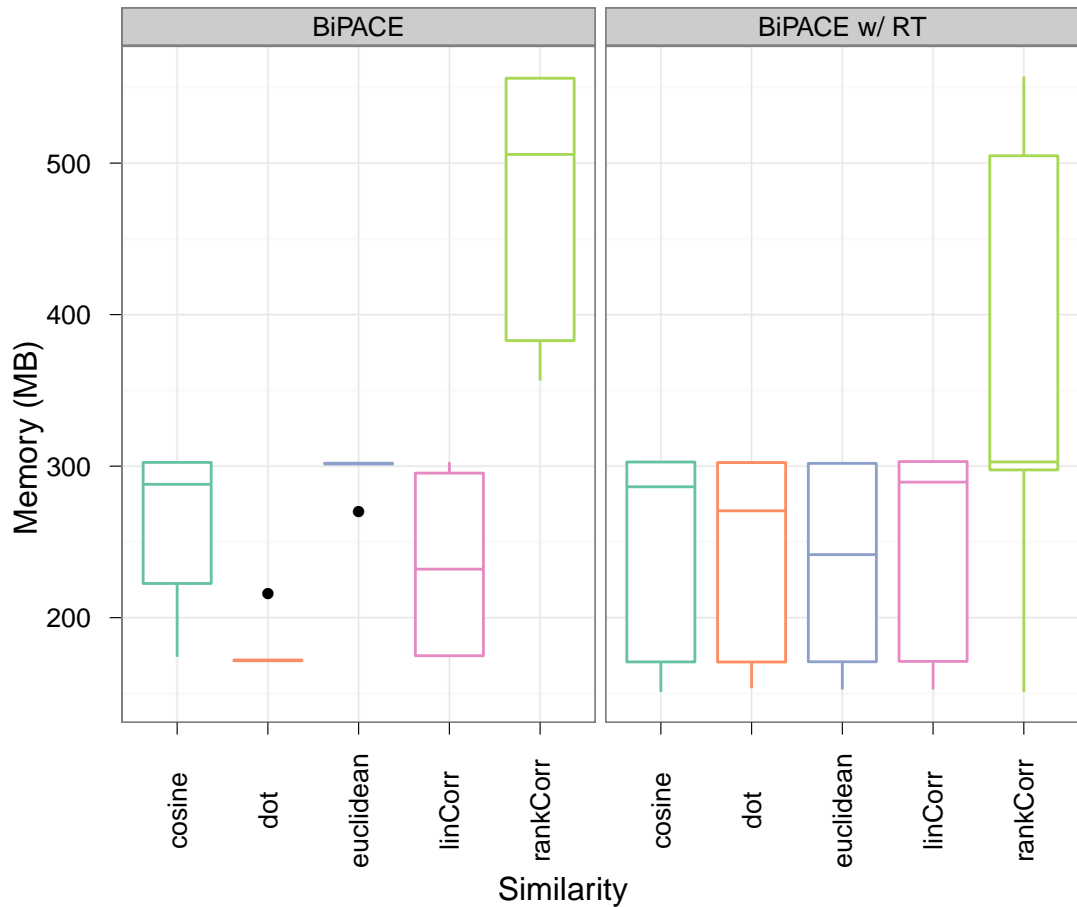

Supplement: Additional file 1 — Archive containing evaluation tables for theLeishmaniaparasite dataset. The complete evaluation table giving the parameters and classification results for BIPACE, CeMAPP-DTW and Robinson’s [10] method for the Leishmania parasite dataset is contained in a zip-archive along with the corresponding figures. Table S1 in the manuscript corresponds to the file ‘evaluation.csv’ in this archive. [file 1471-2105-13-214-S1.zip › leishmania/bipace-memory-histogram.pdf]

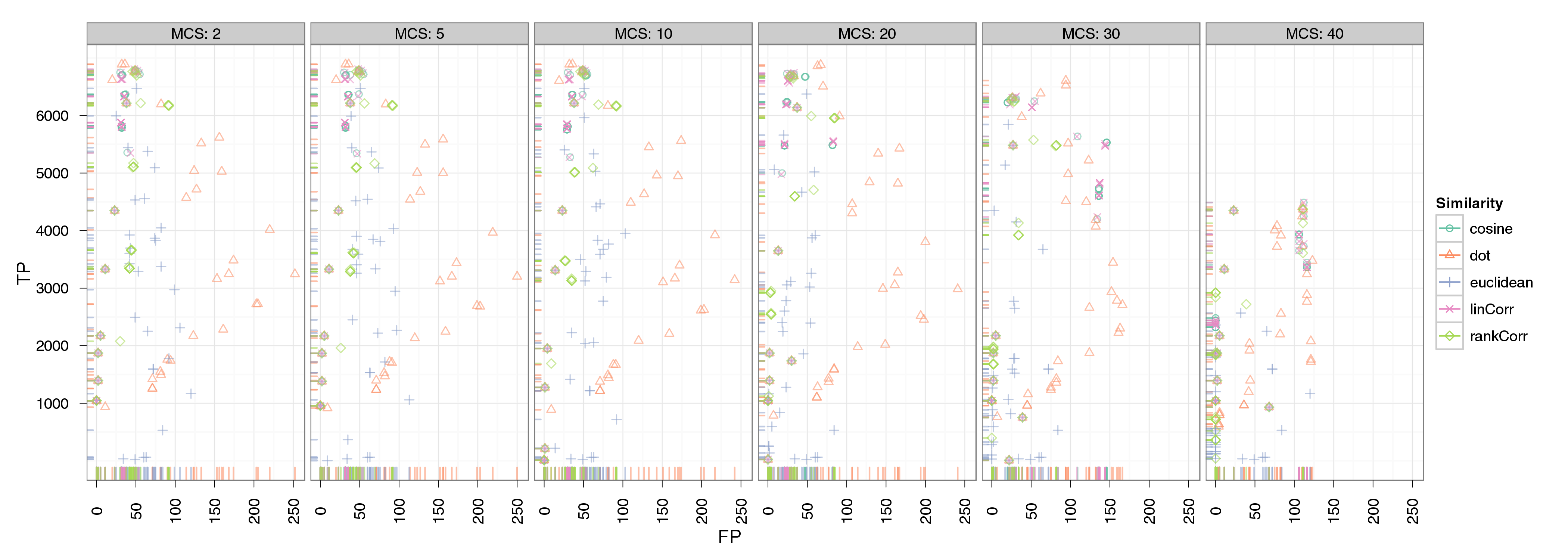

Supplement: Additional file 2 — Archive containing evaluation tables for the wheat dataset. The complete evaluation table giving the parameters and classification results for BIPACE and CeMAPP-DTW for the Wheat dataset is contained in a zip-archive along with the corresponding figures. Table S2 in the manuscript corresponds to the file ‘evaluation.csv’ in this archive. The corresponding raw dataset together with experimental parameters, peak lists and reference multiple peak alignment is available from the Metabolights database at http://www.ebi.ac.uk/metabolights/MTBLS21. [file 1471-2105-13-214-S2.zip › wheat/bipace-fp-vs-tp-mcs.png]

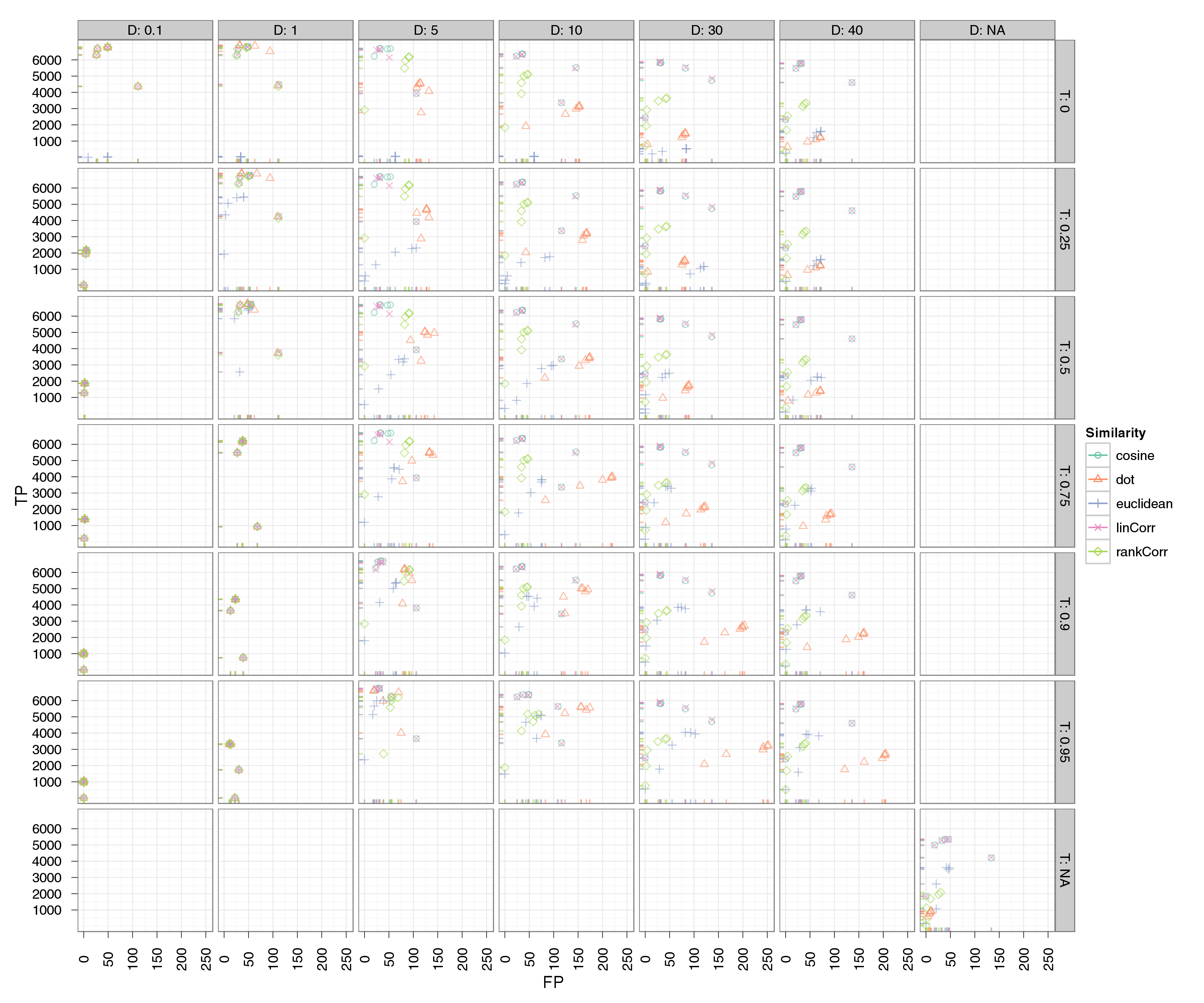

Supplement: Additional file 2 — Archive containing evaluation tables for the wheat dataset. The complete evaluation table giving the parameters and classification results for BIPACE and CeMAPP-DTW for the Wheat dataset is contained in a zip-archive along with the corresponding figures. Table S2 in the manuscript corresponds to the file ‘evaluation.csv’ in this archive. The corresponding raw dataset together with experimental parameters, peak lists and reference multiple peak alignment is available from the Metabolights database at http://www.ebi.ac.uk/metabolights/MTBLS21. [file 1471-2105-13-214-S2.zip › wheat/bipace-fp-vs-tp-rtThres-rtTol.png]

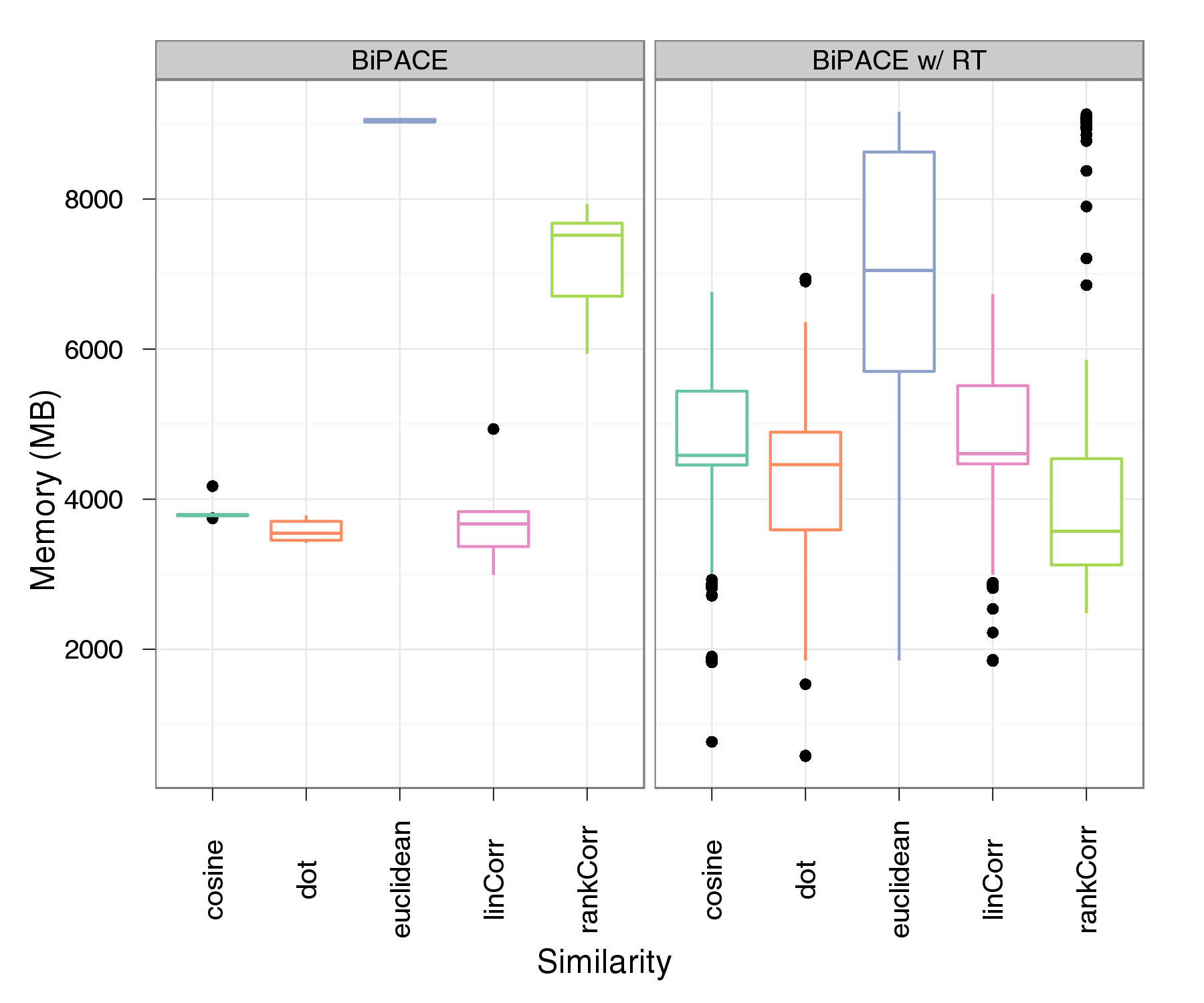

Supplement: Additional file 2 — Archive containing evaluation tables for the wheat dataset. The complete evaluation table giving the parameters and classification results for BIPACE and CeMAPP-DTW for the Wheat dataset is contained in a zip-archive along with the corresponding figures. Table S2 in the manuscript corresponds to the file ‘evaluation.csv’ in this archive. The corresponding raw dataset together with experimental parameters, peak lists and reference multiple peak alignment is available from the Metabolights database at http://www.ebi.ac.uk/metabolights/MTBLS21. [file 1471-2105-13-214-S2.zip › wheat/bipace-memory-histogram.png]

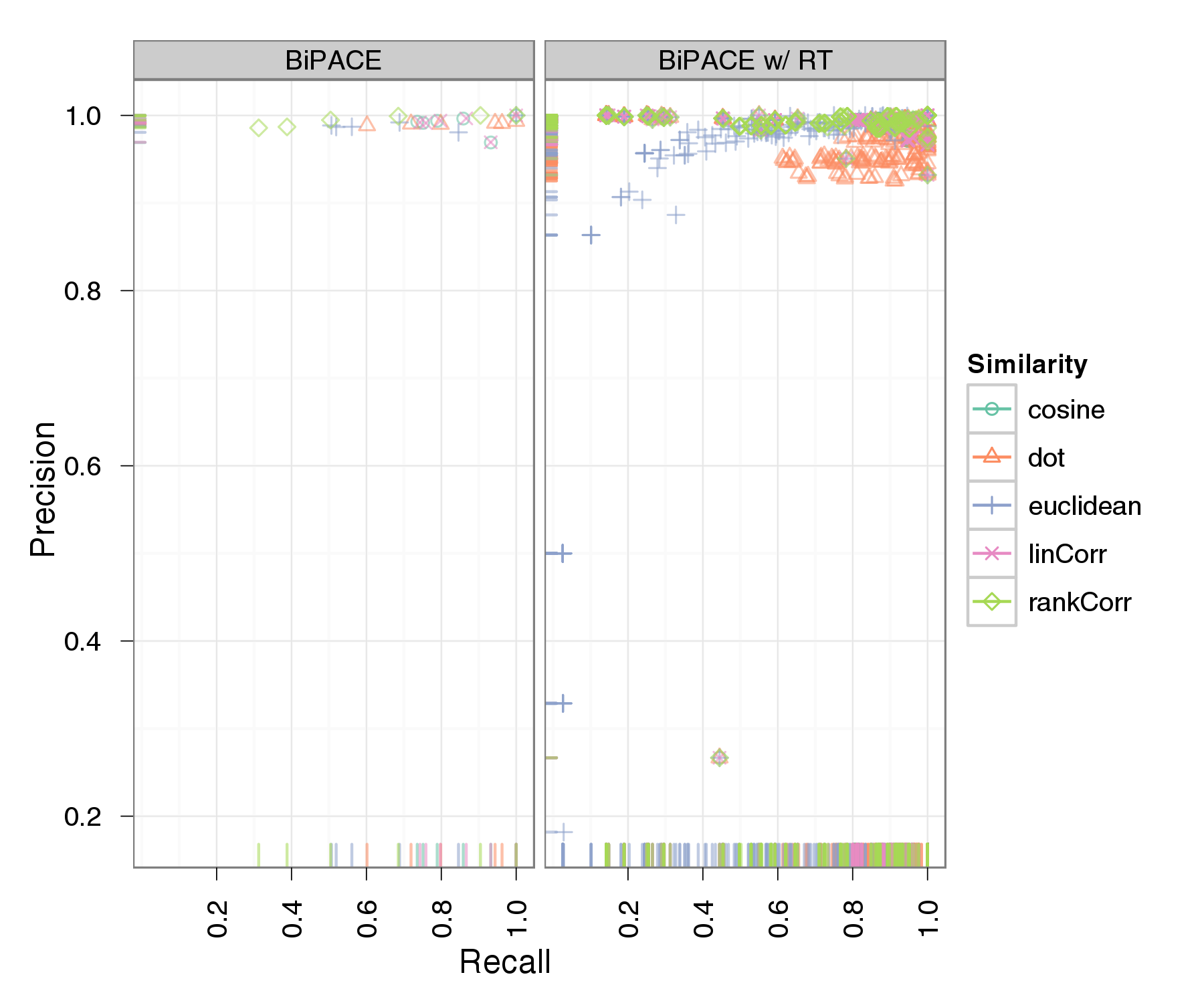

Supplement: Additional file 2 — Archive containing evaluation tables for the wheat dataset. The complete evaluation table giving the parameters and classification results for BIPACE and CeMAPP-DTW for the Wheat dataset is contained in a zip-archive along with the corresponding figures. Table S2 in the manuscript corresponds to the file ‘evaluation.csv’ in this archive. The corresponding raw dataset together with experimental parameters, peak lists and reference multiple peak alignment is available from the Metabolights database at http://www.ebi.ac.uk/metabolights/MTBLS21. [file 1471-2105-13-214-S2.zip › wheat/bipace-recall-vs-precision.png]

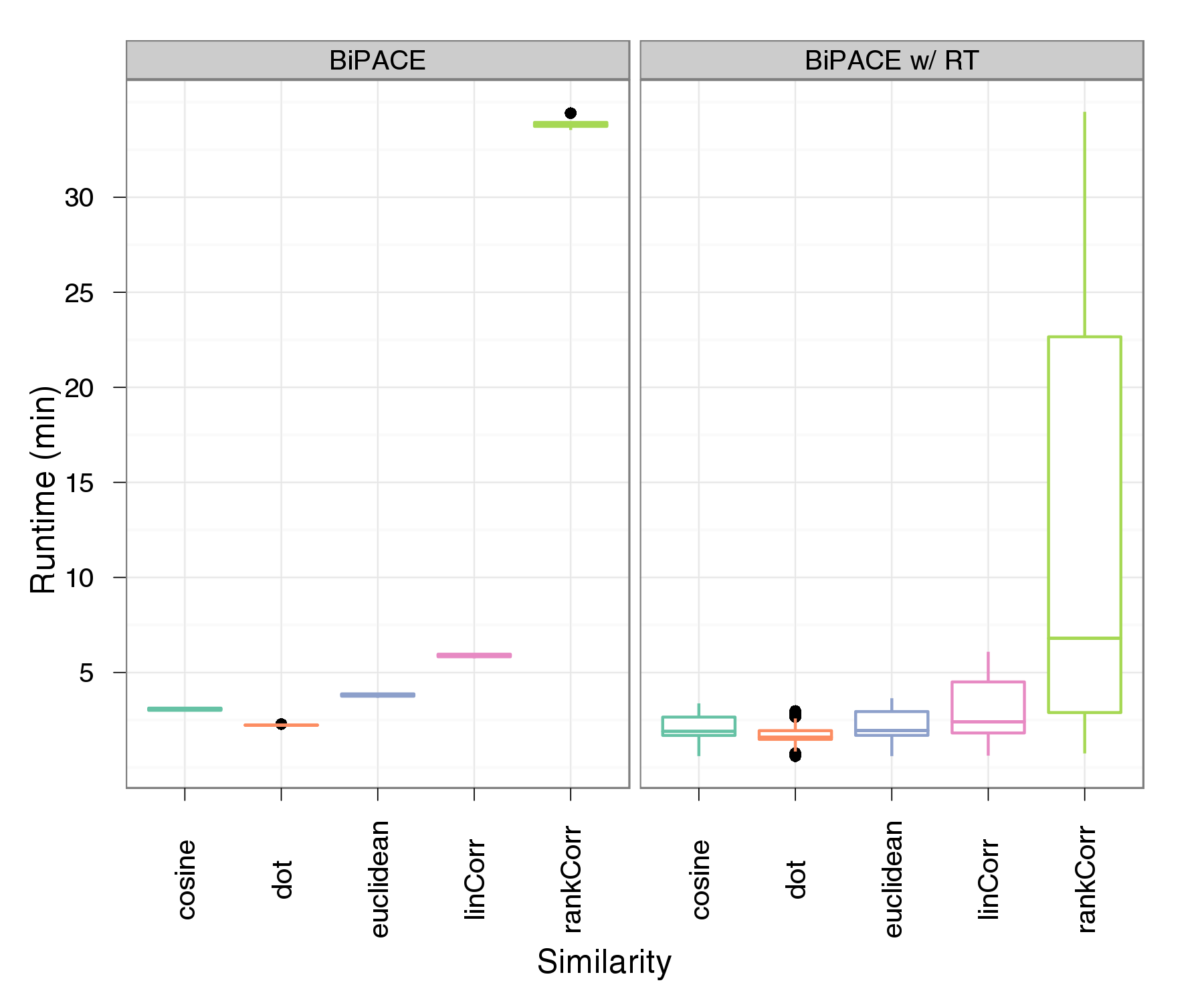

Supplement: Additional file 2 — Archive containing evaluation tables for the wheat dataset. The complete evaluation table giving the parameters and classification results for BIPACE and CeMAPP-DTW for the Wheat dataset is contained in a zip-archive along with the corresponding figures. Table S2 in the manuscript corresponds to the file ‘evaluation.csv’ in this archive. The corresponding raw dataset together with experimental parameters, peak lists and reference multiple peak alignment is available from the Metabolights database at http://www.ebi.ac.uk/metabolights/MTBLS21. [file 1471-2105-13-214-S2.zip › wheat/bipace-runtime-histogram.png]

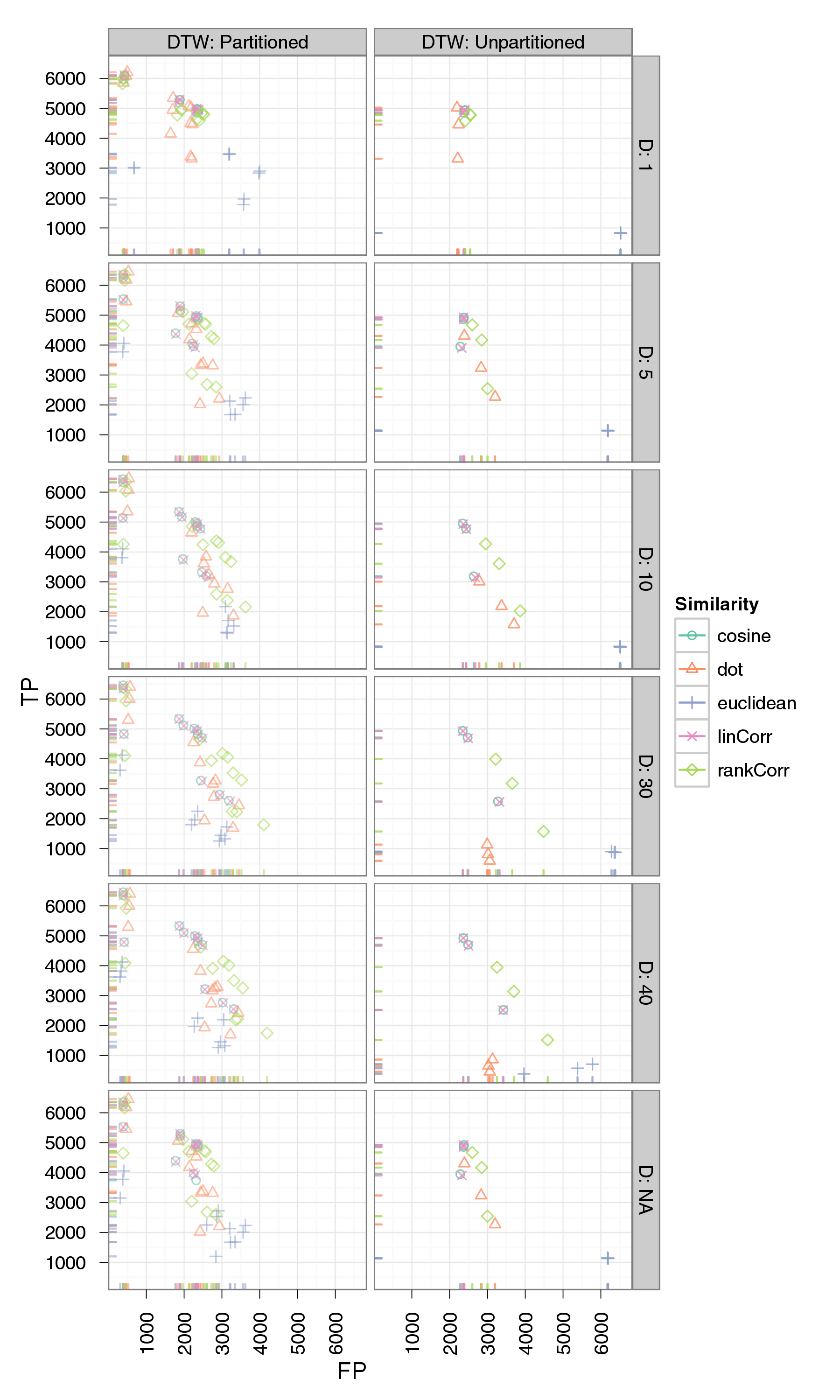

Supplement: Additional file 2 — Archive containing evaluation tables for the wheat dataset. The complete evaluation table giving the parameters and classification results for BIPACE and CeMAPP-DTW for the Wheat dataset is contained in a zip-archive along with the corresponding figures. Table S2 in the manuscript corresponds to the file ‘evaluation.csv’ in this archive. The corresponding raw dataset together with experimental parameters, peak lists and reference multiple peak alignment is available from the Metabolights database at http://www.ebi.ac.uk/metabolights/MTBLS21. [file 1471-2105-13-214-S2.zip › wheat/cemapp-fp-vs-tp-anchor-rtTol.png]

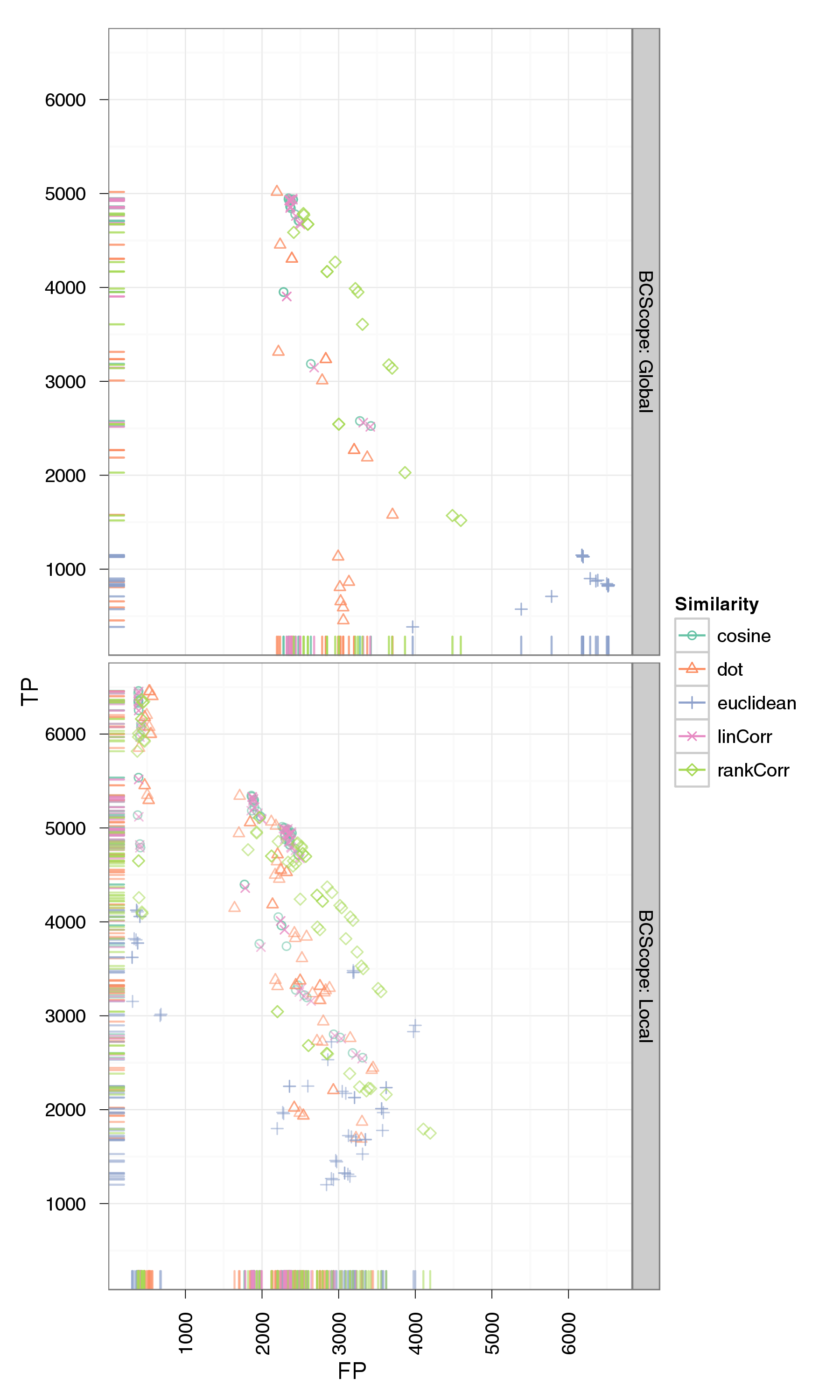

Supplement: Additional file 2 — Archive containing evaluation tables for the wheat dataset. The complete evaluation table giving the parameters and classification results for BIPACE and CeMAPP-DTW for the Wheat dataset is contained in a zip-archive along with the corresponding figures. Table S2 in the manuscript corresponds to the file ‘evaluation.csv’ in this archive. The corresponding raw dataset together with experimental parameters, peak lists and reference multiple peak alignment is available from the Metabolights database at http://www.ebi.ac.uk/metabolights/MTBLS21. [file 1471-2105-13-214-S2.zip › wheat/cemapp-fp-vs-tp-globalBand-BW.png]

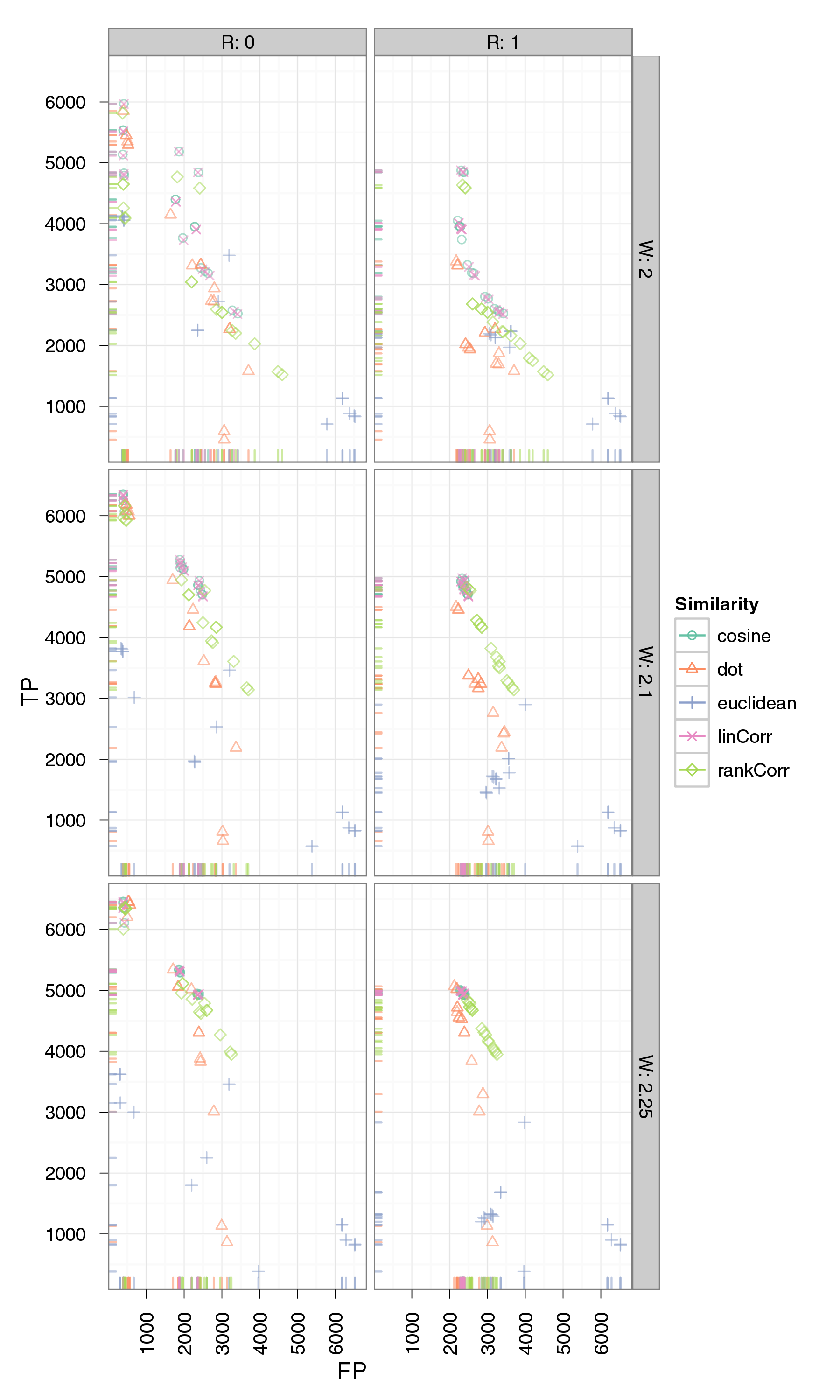

Supplement: Additional file 2 — Archive containing evaluation tables for the wheat dataset. The complete evaluation table giving the parameters and classification results for BIPACE and CeMAPP-DTW for the Wheat dataset is contained in a zip-archive along with the corresponding figures. Table S2 in the manuscript corresponds to the file ‘evaluation.csv’ in this archive. The corresponding raw dataset together with experimental parameters, peak lists and reference multiple peak alignment is available from the Metabolights database at http://www.ebi.ac.uk/metabolights/MTBLS21. [file 1471-2105-13-214-S2.zip › wheat/cemapp-fp-vs-tp-matchWeight-anchorRadius.png]

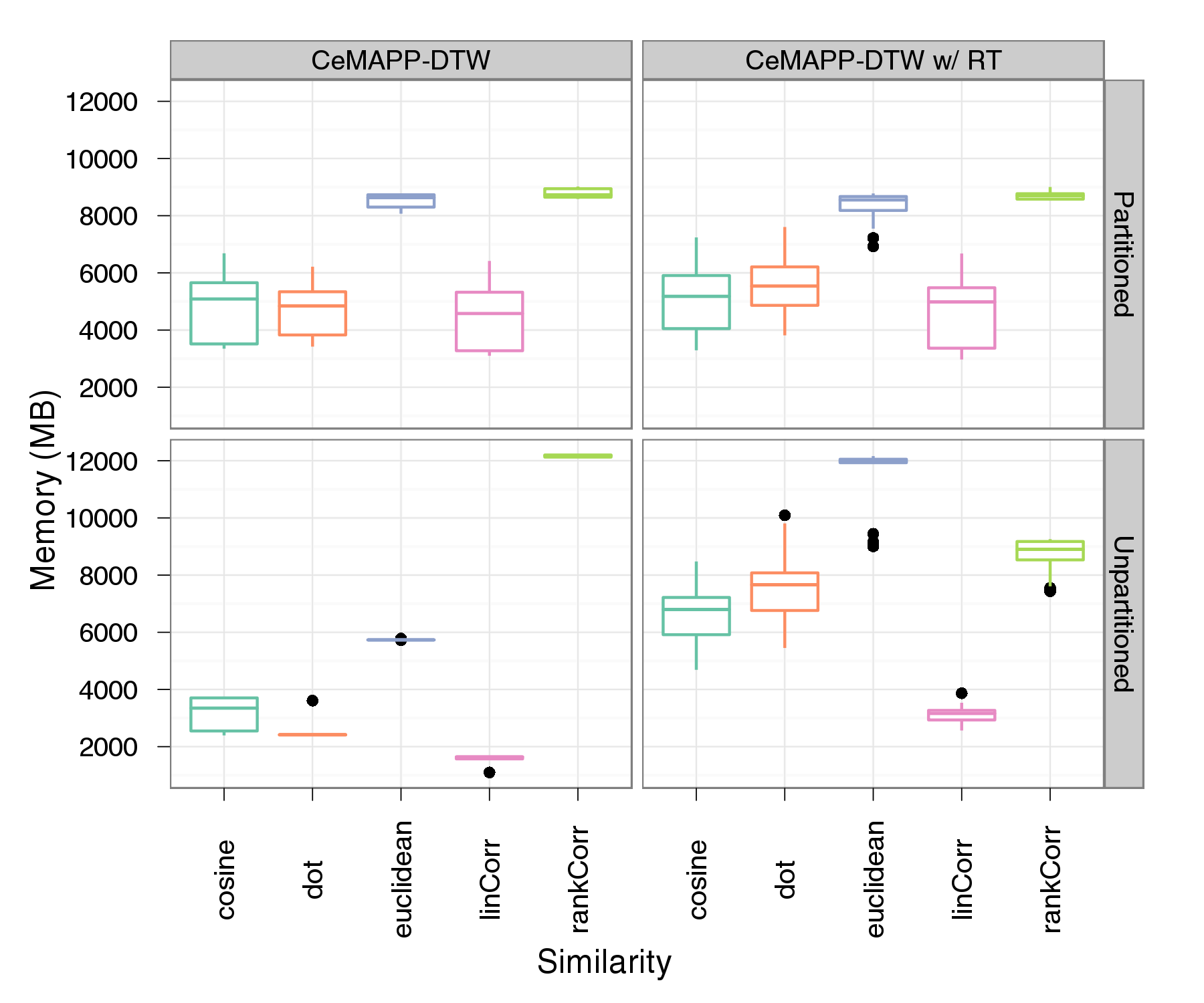

Supplement: Additional file 2 — Archive containing evaluation tables for the wheat dataset. The complete evaluation table giving the parameters and classification results for BIPACE and CeMAPP-DTW for the Wheat dataset is contained in a zip-archive along with the corresponding figures. Table S2 in the manuscript corresponds to the file ‘evaluation.csv’ in this archive. The corresponding raw dataset together with experimental parameters, peak lists and reference multiple peak alignment is available from the Metabolights database at http://www.ebi.ac.uk/metabolights/MTBLS21. [file 1471-2105-13-214-S2.zip › wheat/cemapp-memory-histogram.png]

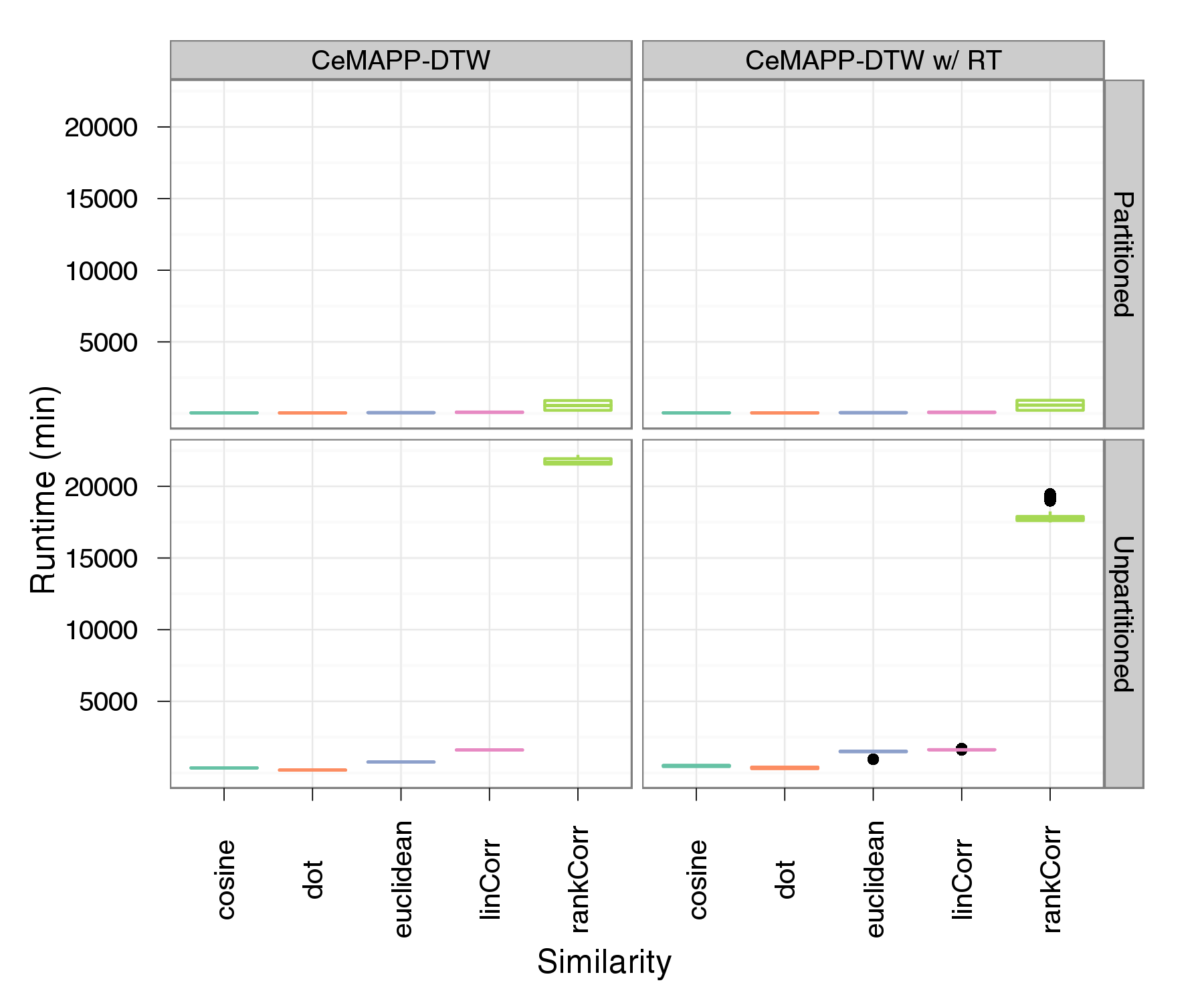

Supplement: Additional file 2 — Archive containing evaluation tables for the wheat dataset. The complete evaluation table giving the parameters and classification results for BIPACE and CeMAPP-DTW for the Wheat dataset is contained in a zip-archive along with the corresponding figures. Table S2 in the manuscript corresponds to the file ‘evaluation.csv’ in this archive. The corresponding raw dataset together with experimental parameters, peak lists and reference multiple peak alignment is available from the Metabolights database at http://www.ebi.ac.uk/metabolights/MTBLS21. [file 1471-2105-13-214-S2.zip › wheat/cemapp-runtime-histogram.png]

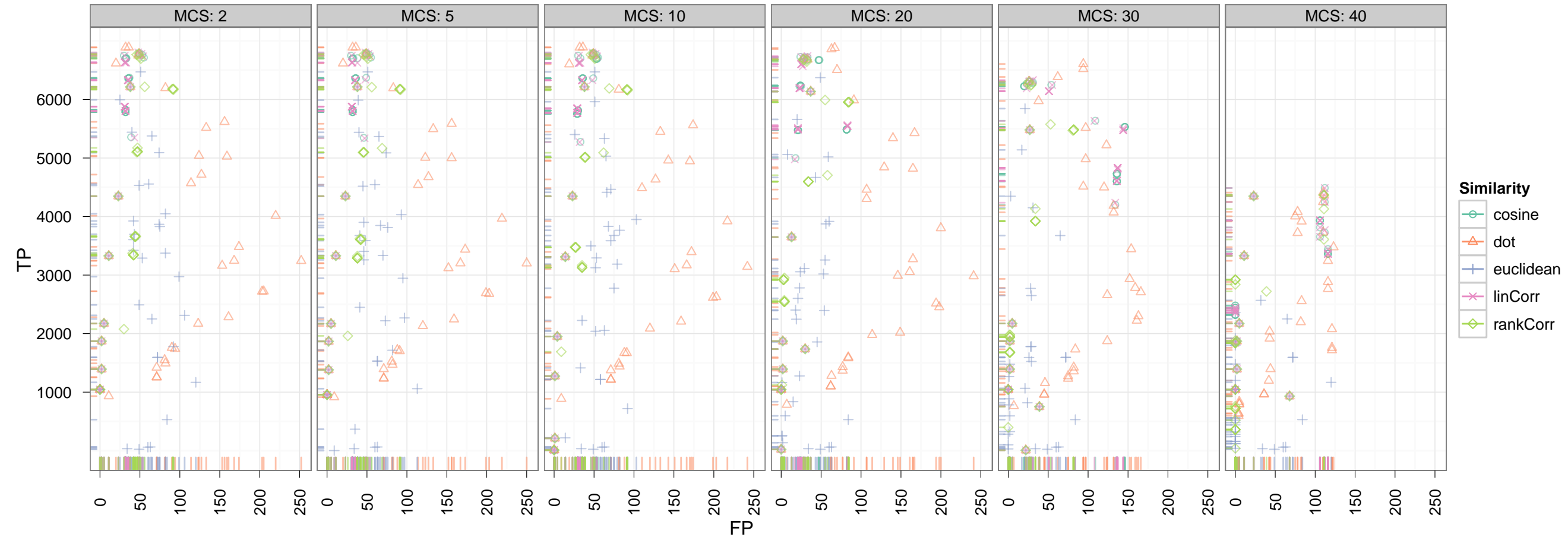

Supplement: Additional file 2 — Archive containing evaluation tables for the wheat dataset. The complete evaluation table giving the parameters and classification results for BIPACE and CeMAPP-DTW for the Wheat dataset is contained in a zip-archive along with the corresponding figures. Table S2 in the manuscript corresponds to the file ‘evaluation.csv’ in this archive. The corresponding raw dataset together with experimental parameters, peak lists and reference multiple peak alignment is available from the Metabolights database at http://www.ebi.ac.uk/metabolights/MTBLS21. [file 1471-2105-13-214-S2.zip › wheat/bipace-fp-vs-tp-mcs.pdf]

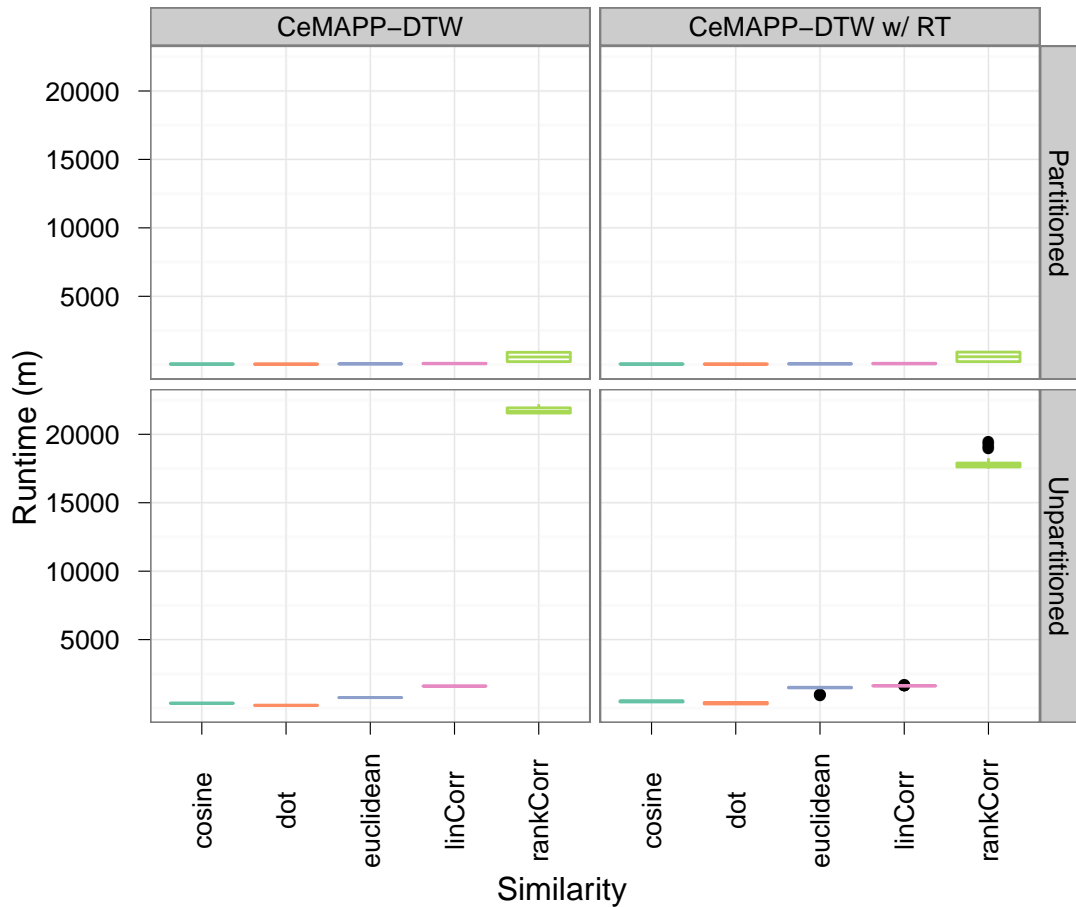

Supplement: Additional file 2 — Archive containing evaluation tables for the wheat dataset. The complete evaluation table giving the parameters and classification results for BIPACE and CeMAPP-DTW for the Wheat dataset is contained in a zip-archive along with the corresponding figures. Table S2 in the manuscript corresponds to the file ‘evaluation.csv’ in this archive. The corresponding raw dataset together with experimental parameters, peak lists and reference multiple peak alignment is available from the Metabolights database at http://www.ebi.ac.uk/metabolights/MTBLS21. [file 1471-2105-13-214-S2.zip › wheat/cemapp-runtime-histogram.pdf]

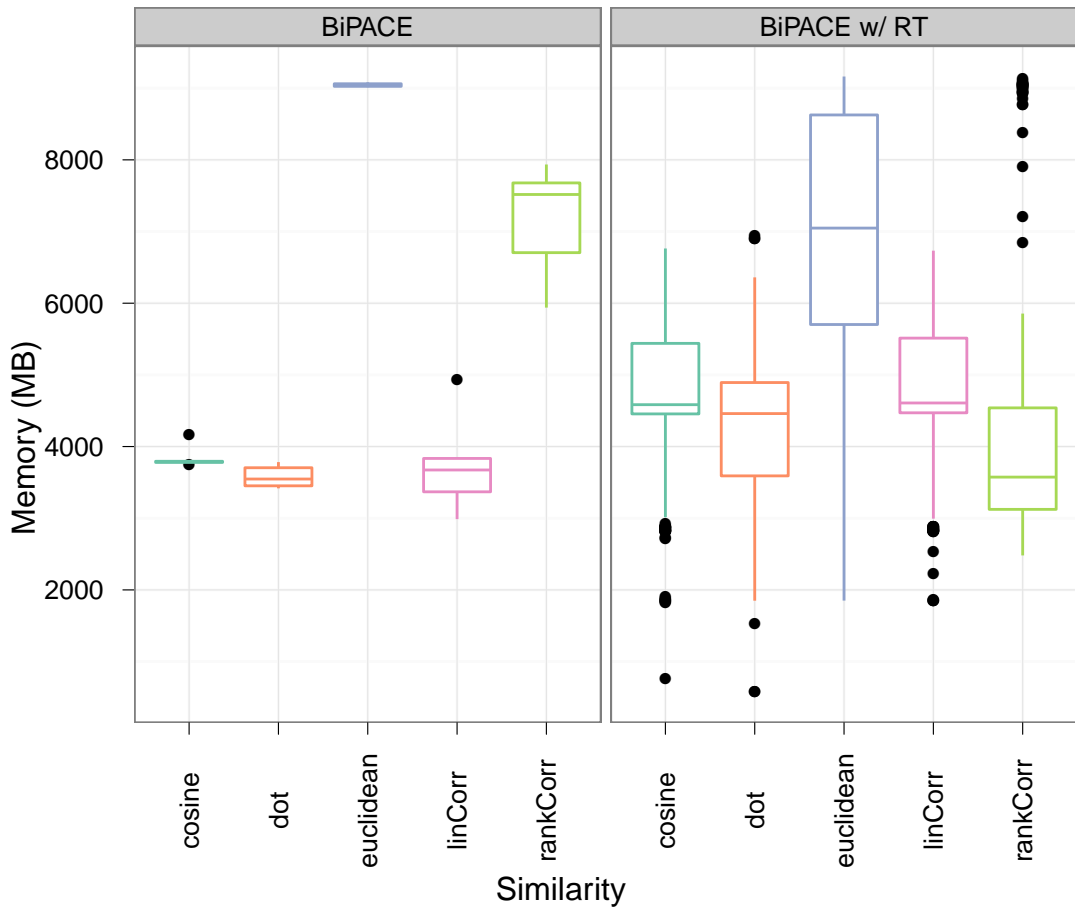

Supplement: Additional file 2 — Archive containing evaluation tables for the wheat dataset. The complete evaluation table giving the parameters and classification results for BIPACE and CeMAPP-DTW for the Wheat dataset is contained in a zip-archive along with the corresponding figures. Table S2 in the manuscript corresponds to the file ‘evaluation.csv’ in this archive. The corresponding raw dataset together with experimental parameters, peak lists and reference multiple peak alignment is available from the Metabolights database at http://www.ebi.ac.uk/metabolights/MTBLS21. [file 1471-2105-13-214-S2.zip › wheat/bipace-memory-histogram.pdf]

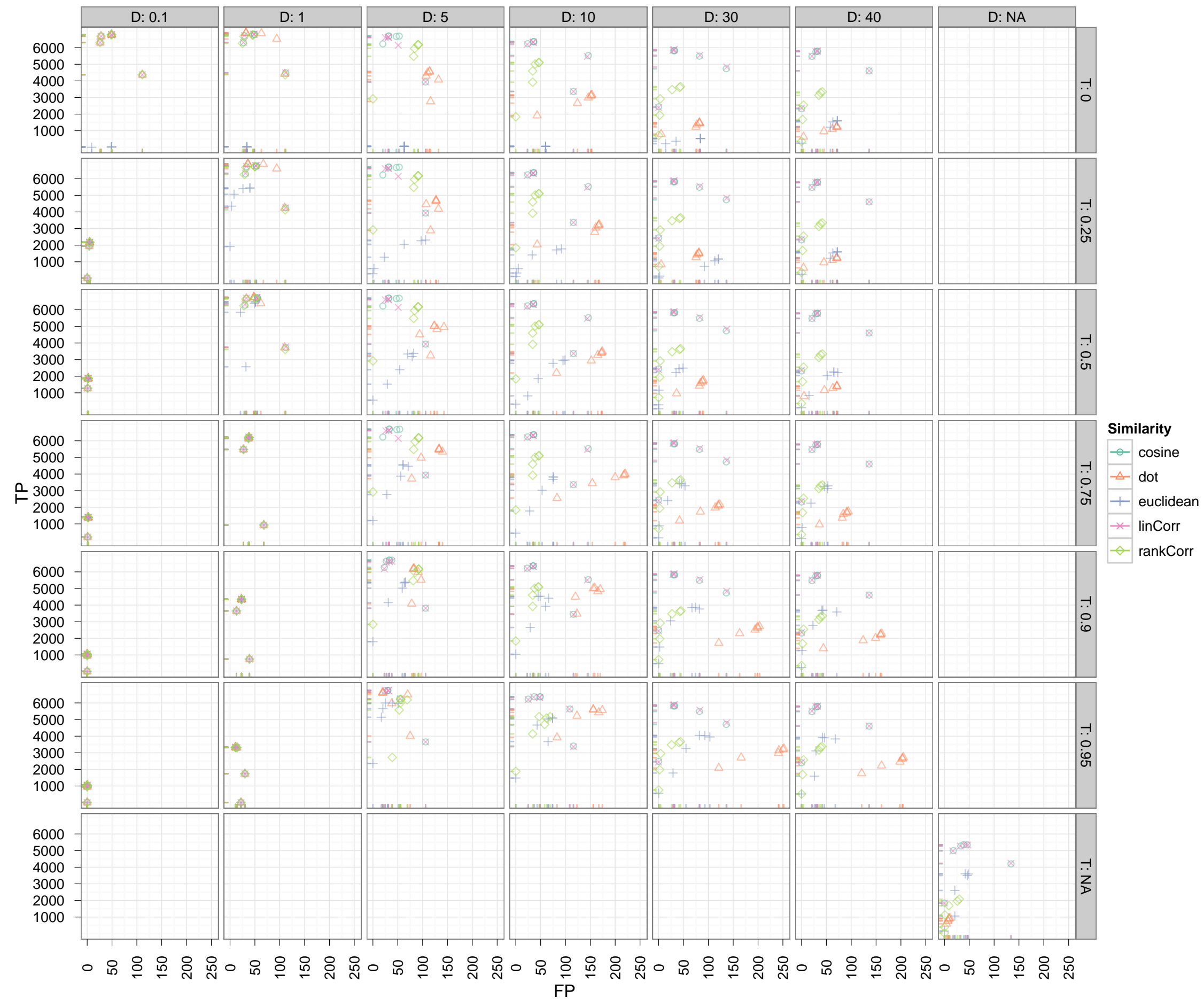

Supplement: Additional file 2 — Archive containing evaluation tables for the wheat dataset. The complete evaluation table giving the parameters and classification results for BIPACE and CeMAPP-DTW for the Wheat dataset is contained in a zip-archive along with the corresponding figures. Table S2 in the manuscript corresponds to the file ‘evaluation.csv’ in this archive. The corresponding raw dataset together with experimental parameters, peak lists and reference multiple peak alignment is available from the Metabolights database at http://www.ebi.ac.uk/metabolights/MTBLS21. [file 1471-2105-13-214-S2.zip › wheat/bipace-fp-vs-tp-rtThres-rtTol.pdf]

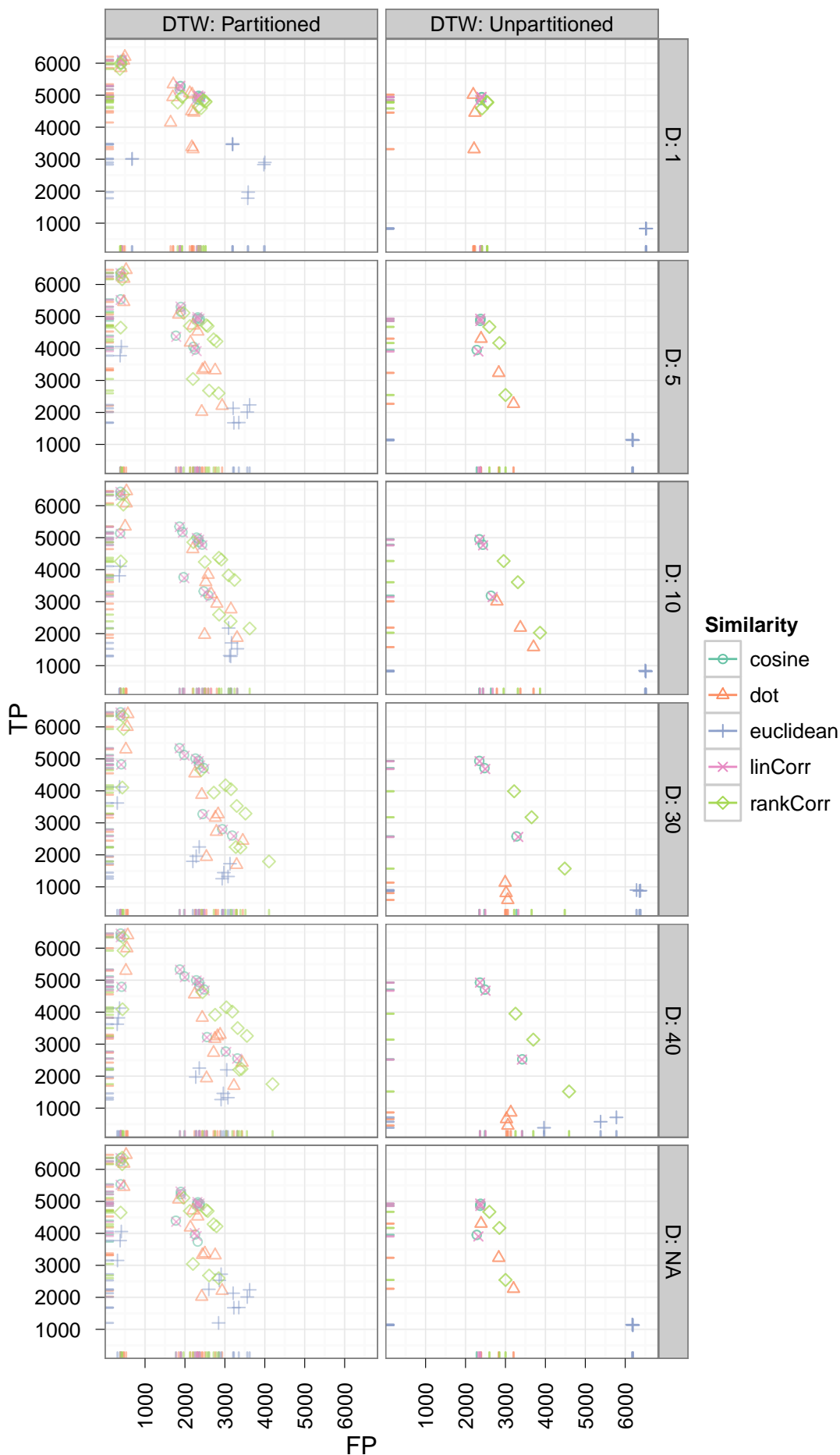

Supplement: Additional file 2 — Archive containing evaluation tables for the wheat dataset. The complete evaluation table giving the parameters and classification results for BIPACE and CeMAPP-DTW for the Wheat dataset is contained in a zip-archive along with the corresponding figures. Table S2 in the manuscript corresponds to the file ‘evaluation.csv’ in this archive. The corresponding raw dataset together with experimental parameters, peak lists and reference multiple peak alignment is available from the Metabolights database at http://www.ebi.ac.uk/metabolights/MTBLS21. [file 1471-2105-13-214-S2.zip › wheat/cemapp-fp-vs-tp-anchor-rtTol.pdf]

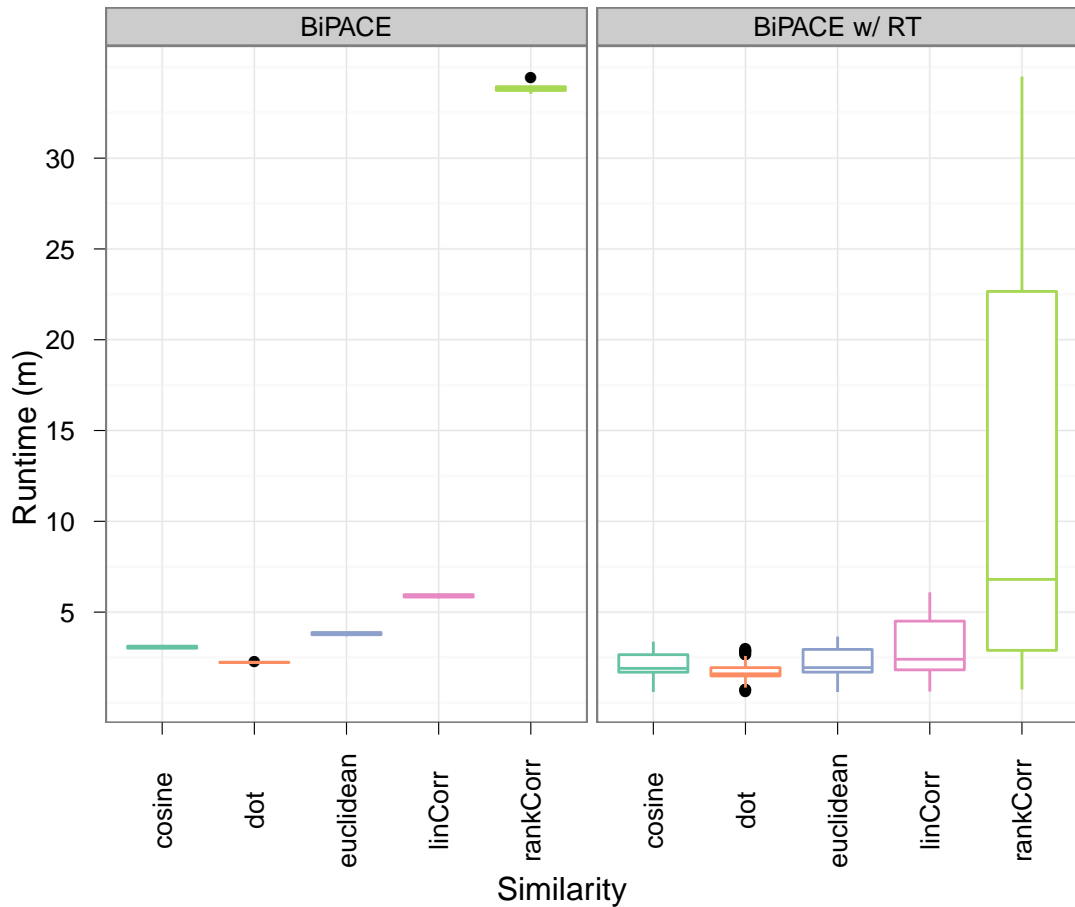

Supplement: Additional file 2 — Archive containing evaluation tables for the wheat dataset. The complete evaluation table giving the parameters and classification results for BIPACE and CeMAPP-DTW for the Wheat dataset is contained in a zip-archive along with the corresponding figures. Table S2 in the manuscript corresponds to the file ‘evaluation.csv’ in this archive. The corresponding raw dataset together with experimental parameters, peak lists and reference multiple peak alignment is available from the Metabolights database at http://www.ebi.ac.uk/metabolights/MTBLS21. [file 1471-2105-13-214-S2.zip › wheat/bipace-runtime-histogram.pdf]

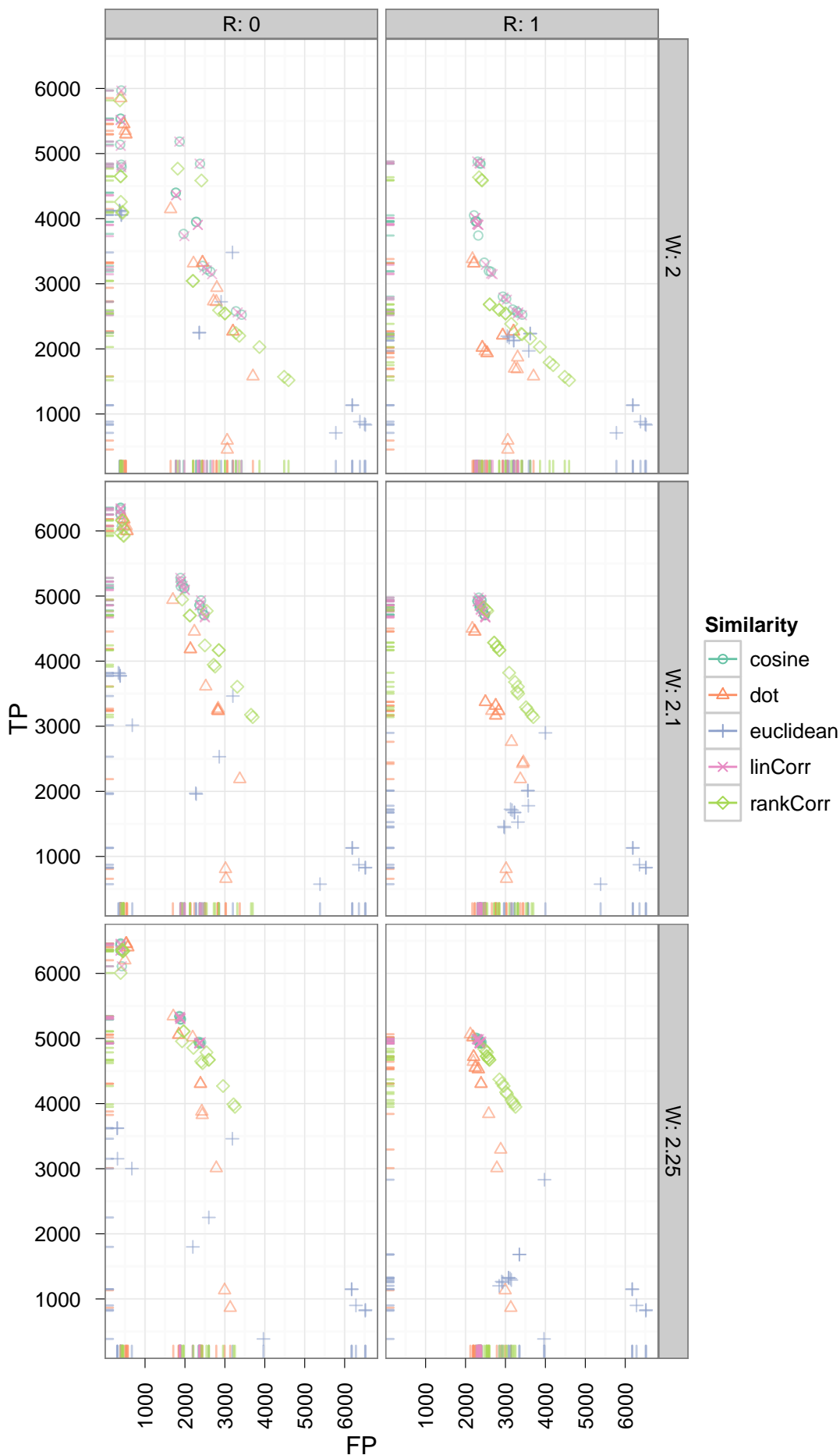

Supplement: Additional file 2 — Archive containing evaluation tables for the wheat dataset. The complete evaluation table giving the parameters and classification results for BIPACE and CeMAPP-DTW for the Wheat dataset is contained in a zip-archive along with the corresponding figures. Table S2 in the manuscript corresponds to the file ‘evaluation.csv’ in this archive. The corresponding raw dataset together with experimental parameters, peak lists and reference multiple peak alignment is available from the Metabolights database at http://www.ebi.ac.uk/metabolights/MTBLS21. [file 1471-2105-13-214-S2.zip › wheat/cemapp-fp-vs-tp-matchWeight-anchorRadius.pdf]

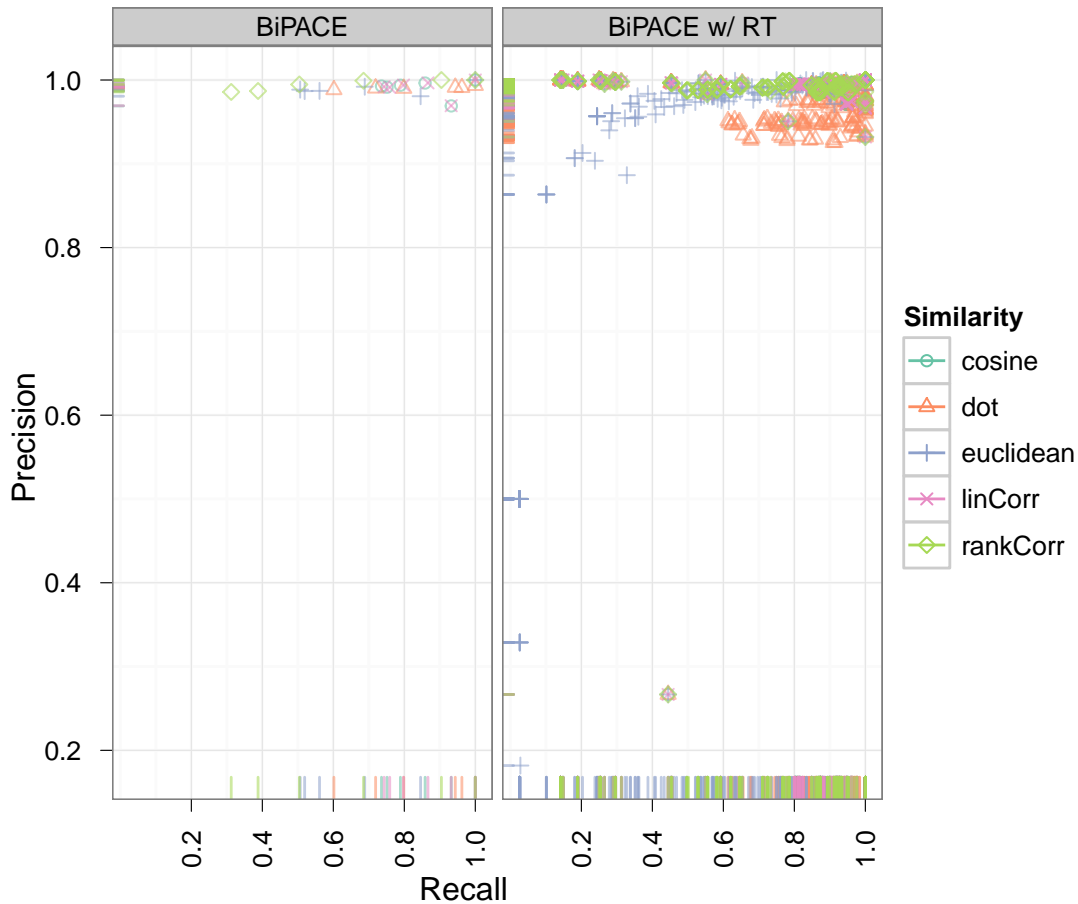

Supplement: Additional file 2 — Archive containing evaluation tables for the wheat dataset. The complete evaluation table giving the parameters and classification results for BIPACE and CeMAPP-DTW for the Wheat dataset is contained in a zip-archive along with the corresponding figures. Table S2 in the manuscript corresponds to the file ‘evaluation.csv’ in this archive. The corresponding raw dataset together with experimental parameters, peak lists and reference multiple peak alignment is available from the Metabolights database at http://www.ebi.ac.uk/metabolights/MTBLS21. [file 1471-2105-13-214-S2.zip › wheat/bipace-recall-vs-precision.pdf]

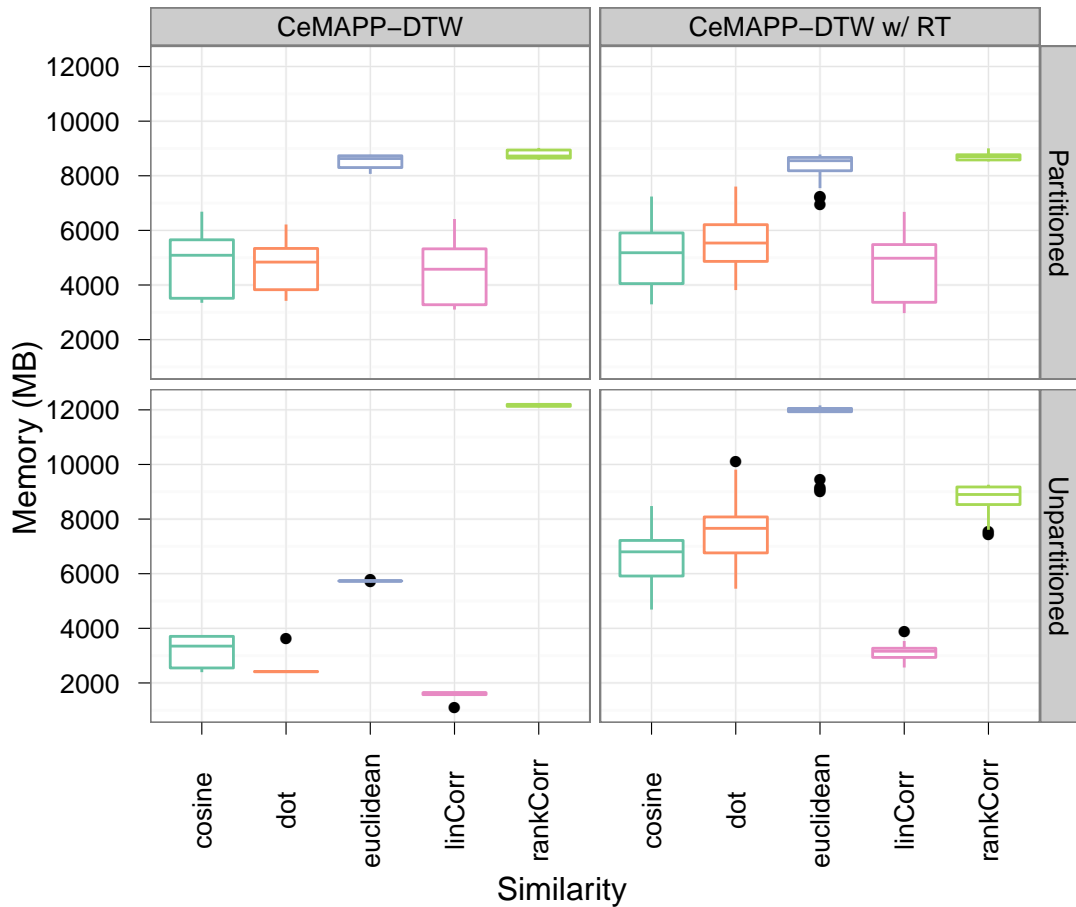

Supplement: Additional file 2 — Archive containing evaluation tables for the wheat dataset. The complete evaluation table giving the parameters and classification results for BIPACE and CeMAPP-DTW for the Wheat dataset is contained in a zip-archive along with the corresponding figures. Table S2 in the manuscript corresponds to the file ‘evaluation.csv’ in this archive. The corresponding raw dataset together with experimental parameters, peak lists and reference multiple peak alignment is available from the Metabolights database at http://www.ebi.ac.uk/metabolights/MTBLS21. [file 1471-2105-13-214-S2.zip › wheat/cemapp-memory-histogram.pdf]

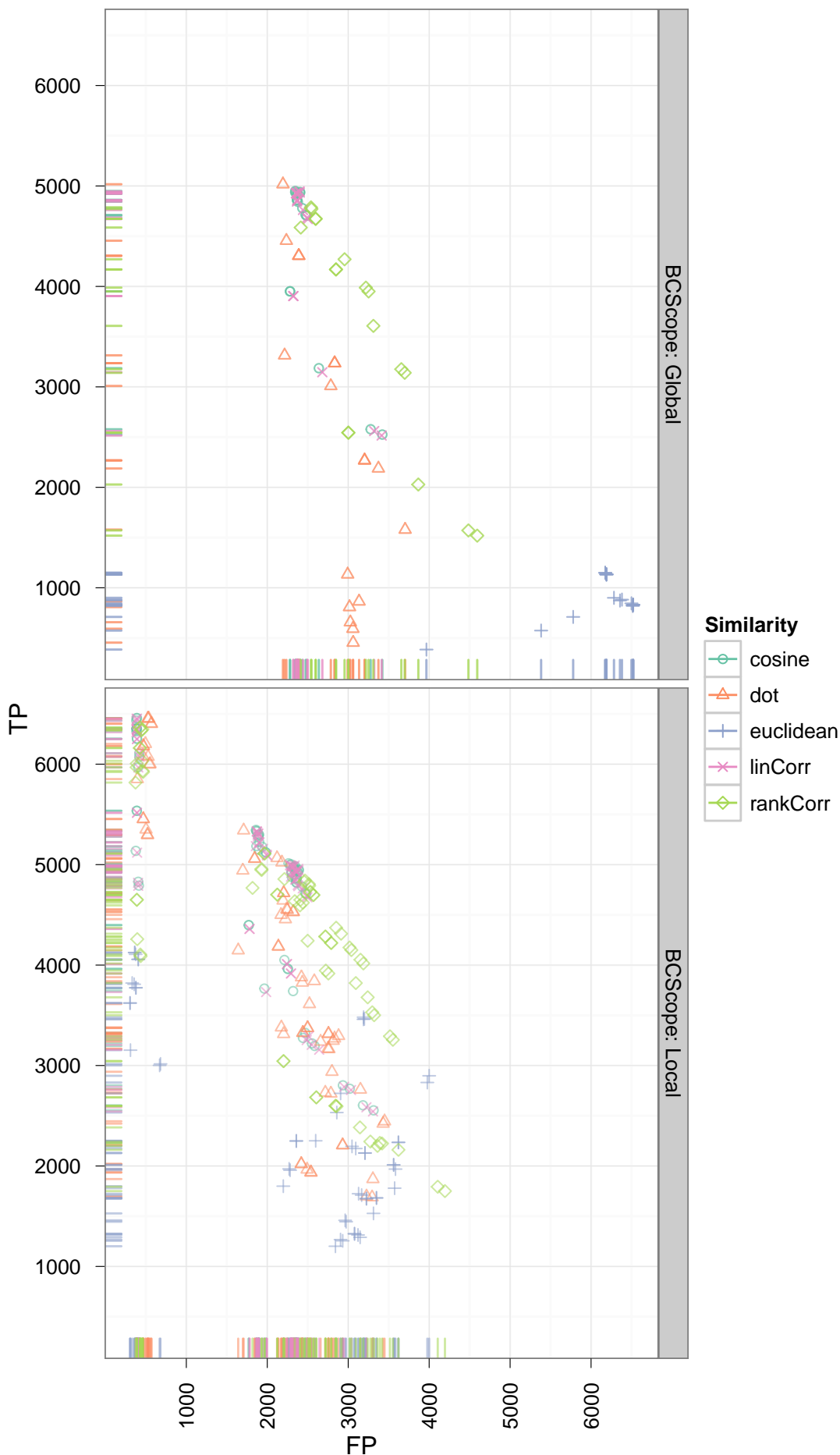

Supplement: Additional file 2 — Archive containing evaluation tables for the wheat dataset. The complete evaluation table giving the parameters and classification results for BIPACE and CeMAPP-DTW for the Wheat dataset is contained in a zip-archive along with the corresponding figures. Table S2 in the manuscript corresponds to the file ‘evaluation.csv’ in this archive. The corresponding raw dataset together with experimental parameters, peak lists and reference multiple peak alignment is available from the Metabolights database at http://www.ebi.ac.uk/metabolights/MTBLS21. [file 1471-2105-13-214-S2.zip › wheat/cemapp-fp-vs-tp-globalBand-BW.pdf]
